# Methylation of Structured RNA by the m^6^A Writer METTL16 Is Essential for Mouse Embryonic Development

**DOI:** 10.1016/j.molcel.2018.08.004

**Published:** 2018-09-20

**Authors:** Mateusz Mendel, Kuan-Ming Chen, David Homolka, Pascal Gos, Radha Raman Pandey, Andrew A. McCarthy, Ramesh S. Pillai

**Affiliations:** 1 Department of Molecular Biology, Science III, University of Geneva, 30 Quai Ernest-Ansermet, CH-1211 Geneva 4, Switzerland; 2 European Molecular Biology Laboratory, Grenoble Outstation, 71 Avenue des Martyrs, 38042 Grenoble, France

**Keywords:** METTL16, m^6^A, Mat2a, U6 snRNA, SAM synthetase, splicing, SAM availability, morula, blastocysts, crystal structure

## Abstract

Internal modification of RNAs with *N*^*6*^-methyladenosine (m^6^A) is a highly conserved means of gene expression control. While the METTL3/METTL14 heterodimer adds this mark on thousands of transcripts in a single-stranded context, the substrate requirements and physiological roles of the second m^6^A writer METTL16 remain unknown. Here we describe the crystal structure of human METTL16 to reveal a methyltransferase domain furnished with an extra N-terminal module, which together form a deep-cut groove that is essential for RNA binding. When presented with a random pool of RNAs, METTL16 selects for methylation-structured RNAs where the critical adenosine is present in a bulge. Mouse 16-cell embryos lacking *Mettl16* display reduced mRNA levels of its methylation target, the SAM synthetase *Mat2a*. The consequence is massive transcriptome dysregulation in ∼64-cell blastocysts that are unfit for further development. This highlights the role of an m^6^A RNA methyltransferase in facilitating early development via regulation of SAM availability.

## Highlights

• Structure of the METTL16 m^6^A writer domain with a unique N-terminal module
• N-terminal module of METTL16 is essential for charge-based binding to RNA
• METTL16 preferentially methylates adenosines within structured RNAs
• Regulation of *Mat2a* mRNA by *Mettl16* is essential for mouse embryonic development

## Graphical Abstract

**Figure:**
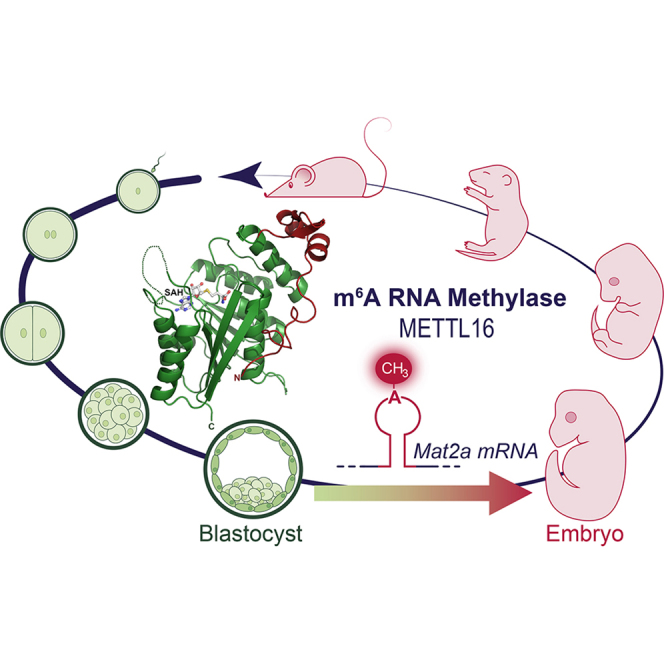

## Teaser

Mendel et al. reveal the structural basis for structured RNA recognition by the mammalian m^6^A writer METTL16 and demonstrate its essential role in mouse early embryonic development via regulation of the SAM synthetase *Mat2a* mRNA.

## Introduction

Methylation of adenosines at the *N*^*6*^ position (*N*^*6*^-methyladenosine or m^6^A) is a highly conserved internal RNA modification with a huge impact on gene regulation (Fu et al., 2014). The modification is added by methyltransferase “writers” and can be removed by RNA demethylase “erasers,” and a major part of its functions is mediated by YTH domain “reader” proteins that can recognize the m^6^A mark. Readers of the m^6^A modification are shown to modulate mRNA splicing, RNA export, RNA stability, and translation (Patil et al., 2018). Alterations in RNA structure are also a consequence of m^6^A methylation (Liu et al., 2015). The m^6^A pathway is physiologically important, as mutations in the writer protein METTL3 in mice lead to embryonic lethality (Batista et al., 2014, Geula et al., 2015), while in flies it affects sex determination (Haussmann et al., 2016, Lence et al., 2016). The only nuclear reader protein YTHDC1 is essential for early embryonic development, and its conditional deletion causes infertility in the germline where it acts via modulation of splicing and alternative polyadenylation site usage (Kasowitz et al., 2018). Loss of the cytoplasmic reader YTHDF2 in fish impairs embryonic development as a result of defective maternal RNA clearance during maternal-zygotic transition (Zhao et al., 2017), while in mice loss of YTHDF2 results in defective maternal RNA metabolism during oocyte maturation, leading to female-specific infertility (Ivanova et al., 2017). In contrast, mouse YTHDC2 is essential for proper progression through meiosis and fertility in both sexes (Bailey et al., 2017, Hsu et al., 2017, Jain et al., 2018, Wojtas et al., 2017). Thus, gene regulation by m^6^A plays a critical role in a variety of developmental processes.

The heterodimeric m^6^A writer complex METTL3/METTL14 co-transcriptionally (Knuckles et al., 2017, Slobodin et al., 2017) installs this mark on thousands of transcripts in the cell (Dominissini et al., 2012, Schwartz et al., 2013). While METTL3 is the active component, METTL14 facilitates substrate RNA binding (Śledź and Jinek, 2016, Wang et al., 2016a, Wang et al., 2016b). METTL16 is the second m^6^A methyltransferase identified, and its known substrates include U6 snRNA and the human *MAT2A* mRNA that encodes for *S*-adenosylmethionine (SAM) synthetase (Pendleton et al., 2017). SAM is a methyl donor for methylation reactions in the cell, including those of DNA, RNA, and protein. While METTL3 prefers to methylate single-stranded RNAs (ssRNAs) in a sequence context RR**A**CH (R = A or G; H = A,C or U), METTL16 uses structured RNAs carrying a specific nonamer sequence (UAC**A**GAGAA; methylated adenosine is underlined) (Pendleton et al., 2017). Methylation of *MAT2A* mRNA within specific hairpin structures in the 3ʹ UTR is proposed to be used by YTHDC1 to mediate downregulation of the mRNA under high-SAM conditions (Shima et al., 2017). Apart from this enzymatic role, METTL16 is also reported to act as a splicing enhancer during low-SAM conditions when it occupies its binding site on the six *MAT2A* hairpins (hp) to promote splicing of a 3ʹ terminal intron that is frequently retained. This results in increased mature *MAT2A* mRNA production and acts as a feedback loop ensuring optimal production of the SAM synthetase in response to low SAM levels (Pendleton et al., 2017). Unlike the METTL3/METTL14 complex which mainly methylates exonic sequences (Ke et al., 2017), METTL16 was shown to have binding sites on several intronic sequences in pre-mRNAs and structured noncoding RNAs, some of which carry m^6^A marks (Brown et al., 2016, Warda et al., 2017). How METTL16 recognizes its RNA substrates and the physiological importance of having a second m^6^A methyltransferase is currently not known.

METTL16 is a highly conserved enzyme with orthologs found in *E.coli* (Sergiev et al., 2008) to human (Figure S1A). Here, we examine the crystal structure of the methyltransferase (MTase) domain from human METTL16 and identify key features that are essential for RNA binding and methylation activity. We define the RNA substrate requirements *in vitro* using a randomized RNA library to find that structured RNAs with a bulged adenosine are preferred. Finally, we generate a knockout *Mettl16* mouse mutant to show that the protein is essential for early embryonic development. Our studies show that METTL16 is essential for embryonic development around implantation stage and acts via regulation of the *Mat2a* mRNA which encodes the SAM synthetase.

## Results

### Crystal Structures of the Human m^6^A Methyltransferase METTL16

**Figure 1:**
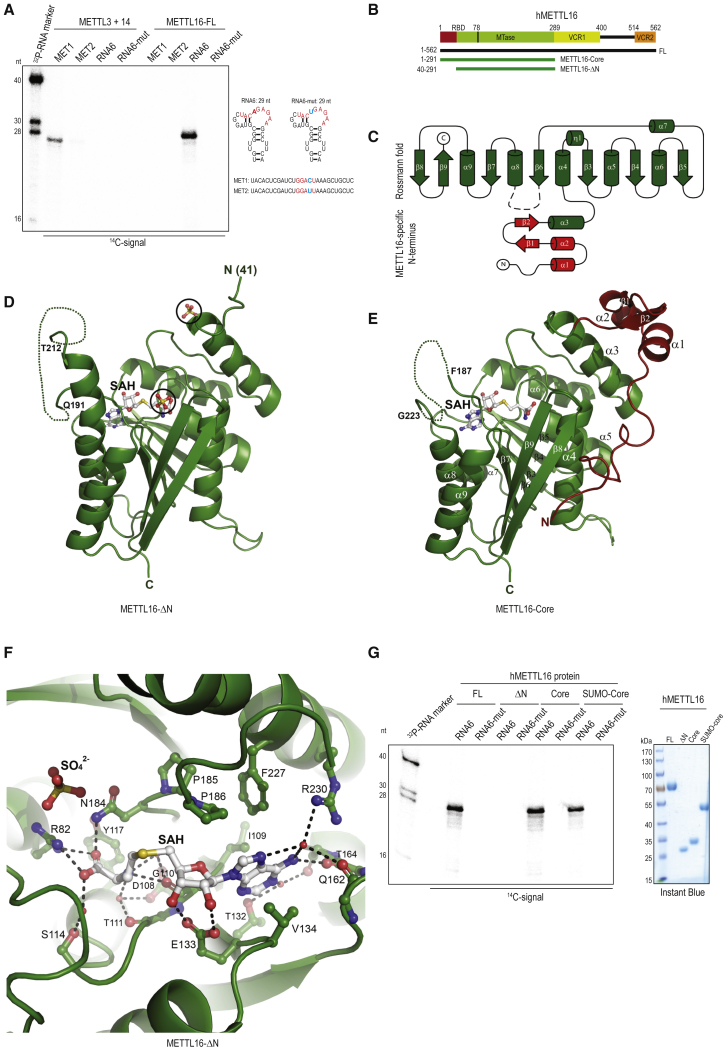
Structure of Human METTL16 Reveals an N-Terminal Module Essential for Activity (A) *In vitro* methylation assays of indicated full-length (FL) human m^6^A methyltransferases with ^14^C-SAM and different RNA substrates (right). Predicted structure of a short hairpin RNA (RNA6) derived from the longer *MAT2A* hairpin 1 (Pendleton et al., 2017) and its mutant (RNA6-mut) with A→U mutation of the methylated adenosine are shown. The MET1 RNA has the consensus site for methylation by the METTL3+METTL14 complex, while the MET2 RNA has a point mutation (C→U) of a conserved residue in the methylation consensus site (see Table S1). Single-stranded RNA markers (length in nucleotides, nt) are ^32^P-end-labeled. See also Figure S1D. (B) Domain architecture of human METTL16. RBD, RNA-binding domain (1–78 aa); MTase, methyltransferase domain; VCR, vertebrate conserved region. Boundaries of the two protein constructs crystallized in this study are indicated (in green). The ΔN version has an N-terminal deletion. (C) Schematic view of the MTase domain. Cylinders represent α helices, and arrows represent β strands. Regions shaded in red (α1-2 and β1-2) are seen only in the METTL16-core structure and together with α3 form a separate N-terminal module. (D) Model of the METTL16-ΔN construct (PDB 6GFK). Two-sulfate (SO_4_^2-^) ions visualized in the crystal structure are circled. A disordered loop between α8 and β6 is connected by a dotted line. SAH, S-adenosyl homocysteine. (E) Model of the METTL16-core construct (PDB 6GFN). The additional regions at the N terminus seen in this structure are shown in red. See also Figure S1G. (F) A zoom of the catalytic pocket in the METTL16-ΔN structure showing coordination of SAH. Catalytic residues N184, P185, P186, and F187 and position of a sulfate (SO_4_^2-^) ion are indicated. See also Figure S2A. (G) *In vitro* methylation assays showing that METTL16-ΔN protein is inactive. The METTL16-Core protein was used as untagged or tagged (SUMO) versions. See also Figure S2C. Quality of proteins used is shown on the right. Protein molecular weight markers (in kilo Daltons, kDa) are indicated.

We produced the recombinant full-length (FL) human METTL3/METTL14 heterodimeric complex and FL human METTL16 (1–562 aa) in a eukaryotic expression system (Figures S1B and S1C; Star Methods). Together with the methyl donor *S*-adenosylmethionine (SAM), the enzymes were presented with either a single-stranded RNA (ssRNA, MET1) carrying the RR**A**CH consensus site or a 29 nt hairpin RNA (RNA6) derived from the human *MAT2A* mRNA, carrying the nonamer methylation site for METTL16 (UAC**A**GAGAA) (Table S1) (Pendleton et al., 2017). While the METTL3/METTL14 complex efficiently methylated the ssRNA, it did not use the hairpin RNA as a substrate (Figure 1A). On the contrary, METTL16-FL methylated only the hairpin substrate, but not the ssRNA. Both enzymes also sensed the sequence context of their respective substrates, as single nucleotide mutations within the RNA consensus sites either reduced (for METTL3/14 complex) or abolished (for METTL16) the methylation activity (Figure 1A). The METTL16-FL protein was also capable of using U6 snRNA and the full-length *MAT2A* hairpin (hp) 1 as substrates for methylation (Figure S1D). Thus, the purified m^6^A methyltransferases are able to discriminate their respective RNA substrates *in vitro*.

**Figure 2:**
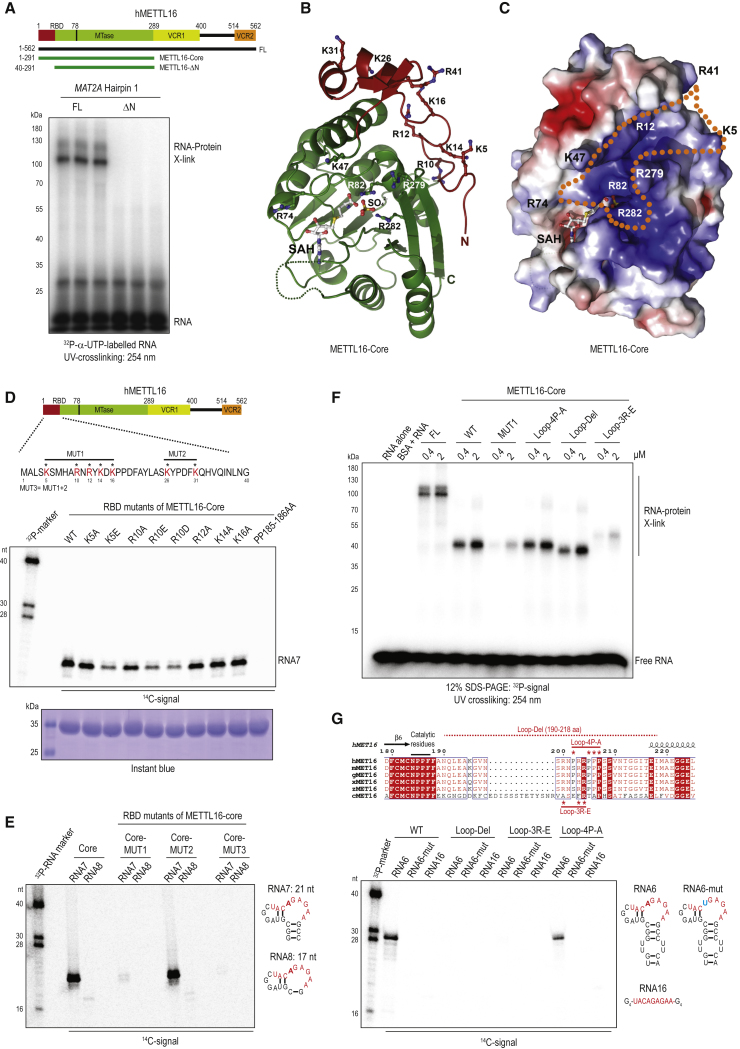
The N-Terminal Module of Human METTL16 Is Required for Substrate RNA Binding (A) Domain architecture of human METTL16. UV crosslinking assay (triplicate reactions) showing RNA-protein crosslinks (X-link) between human full-length (FL) METTL16 and *MAT2A* hairpin 1 RNA. See also Figure S2C for *in vitro* methylation with the same proteins and RNA. (B) Overview of the METTL16-core MTase domain. Key positively charged residues that create the RNA-binding groove are indicated. Note that residues K26 and K31 when mutated (MUT2) do not affect activity. SAH, S-adenosyl homocysteine; SO_4_^2-^, position of a sulfate ion as seen in METTL16-ΔN is shown. The disordered loop with catalytic residues is shown as a dotted line. (C) Surface charge representation of the METTL16-core MTase domain showing a positively charged (blue) groove (outlined) leading from the N terminus to the catalytic pocket. (D) Cartoon showing the N-terminal 40 amino acids of human METTL16, with the highlighted positively charged residues that were mutated (red, with asterisks). Gel shows the *in vitro* methylation assay with wild-type (WT) or indicated point mutant METTL16-core proteins. Quality of recombinant proteins used is shown below the gel. RNA7 was used as an RNA substrate (see Table S1). Single-stranded RNA markers (length in nucleotides, nt) are ^32^P-end-labeled. See also Figure S2D. (E) *In vitro* methylation assay with RNAs indicated and mutants carrying multiple point mutations on the N-terminal module (see D). See also Figure S2E for additional mutations within the RNA binding groove. (F) UV crosslinking assay with METTL16 proteins indicated and ^32^P-end-labeled RNA6. The positions of the free RNA and RNA-protein crosslinks (X-link) are shown. Control binding reactions are carried out without any protein (RNA alone) or with bovine serum albumin (BSA). (G) Sequence alignment of METTL16 orthologs showing the catalytic residues and disordered loop region. See also Figure S1A. Deletions and mutations introduced into the loop in the context of the METTL16-core construct are indicated. *In vitro* methylation assay with indicated proteins and RNAs is shown below. Quality of proteins used is shown in Figure S2F.

To obtain structural information on METTL16, we identified stable protein domains by limited proteolysis (Figure S1E). Two constructs (core, 1–291 aa; and ΔN, 40–291 aa) encompassing the methyltransferase domain (MTase) were expressed in *E. coli* and crystallized (Star Methods) (Figures 1B and S1B). Consistent with the SAM-dependent methyltransferase activity of METTL16, both structures reveal a Rossmann fold composed of a central seven-stranded β sheet (strands β3–β9) flanked by three α helices each (helices α4–α6 and α7–α9) (Figures 1C–1E). The complexed byproduct of the SAM-dependent methylation reaction, *S*-adenosyl homocysteine (SAH), is coordinated by a hydrogen bond interaction network with the highly conserved (Figure S1A) amino acid residues R82, D108, G110, T111, S114, E133, Q162, N184, and R230 (Figures 1F, S1G, and S2A). The methyl donor SAM is presumed to fit into the same surface-exposed pocket. The catalytic residues NPPF (184–187 aa) are present in a loop positioned in close proximity to the SAH molecule (Figures 1E, 1F, and S2A). Point mutations (PP185–186AA or F187G) of these residues abolish m^6^A RNA methyltransferase activity on a *MAT2A* hairpin substrate when tested *in vitro* (Figures 2D and S2D). All these key features define METTL16 as a SAM-dependent methyltransferase.

Three additional observations can be made from our structures. First, and most striking, is the presence of an N-terminal module (1–78 aa) in the core structure that is appended to the α4 of the Rossmann fold and that consists of three helices (α1–α3) and two short β strands (β1 and β2) (Figures 1C and 1E). This module is flexible, as shown by proteolysis, with only α3 remaining in the ΔN structure (Figures 1D and S1E). Second, the loop containing the catalytic residues NPPF (N184–F187) is part of a larger stretch of 35 amino acids that links β6 to α8, and it is disordered in both structures (dotted lines in Figures 1D and 1E). While 20 residues between Q191 and T212 are not visible in the ΔN structure, a much larger region (35 residues) between F187 and G223 lacks density in the core structure (Figures 1D and 1E). This loop in *C. elegans* is even longer (48 aa) and shows poor overall sequence conservation with its vertebrate orthologs (Figure S1A). Given its strategic location, it may be involved in contacting the bound RNA substrate during catalysis. Third, the ΔN structure reveals the presence of two sulfate ions (SO_4_^2-^) (Figure 1D), one of which is next to R82 (one of the residues coordinating SAH), likely mimicking how an RNA substrate might access the catalytic pocket (Figure 1F).

To test whether these structures are representative of catalytically active versions of the METTL16 MTase domain, we incubated the recombinant proteins with ^14^C-SAM and a 29 nt RNA (RNA6) derived from the *MAT2A* hp 1. The full-length METTL16 and the core domain versions were able to methylate this RNA (RNA6) at a specific adenosine residue (A17) within the nonamer UAC**A**GAGAA motif (Figure 1G). In contrast, a mutant hairpin (RNA6-mut) with A17U mutation was not methylated. To our surprise, even though the ΔN-truncated version (40–291 aa) has a similar conformation in terms of the Rossmann fold and catalytic residues, it was inactive (Figure 1G). A similar situation is seen even when the full-length *MAT2A* hairpin 1 is used (Figure S2C). Taken together, our two structures reveal an architecture where the MTase domain is attached to a METTL16-specific N-terminal module that is essential for activity.

### The N-Terminal Module of METTL16 Is Essential for RNA Binding, while a Disordered Loop Is Required for Catalysis

Given that the METTL16-ΔN domain is inactive as an MTase, we examined whether it can bind RNA substrates using UV crosslinking experiments. Incubation of METTL16-FL with a body-labeled *MAT2A* hp 1 RNA gave an RNA-protein crosslink consistent with RNA binding, while the ΔN version did not reveal such an interaction (Figure 2A). This aligns with the observed lack of methylation activity of the ΔN protein when the same RNA substrate was used (Figure S2C). Examination of the primary sequence of the N-terminal module revealed the presence of several highly conserved positively charged residues that can potentially be involved in mediating interaction with RNAs (Figure S1A). Indeed, when mapped onto the METTL16-core structure, these reveal a positively charged cluster (K5, R10, R12, K14, K16, and R41) that forms the entrance of a wide deep-cut groove (Figures 2B and 2C). With additional contributions from the N-terminal module (K47 and R74) and those from within the Rossmann fold (R82, R279, and R282), the groove runs all the way to the catalytic pocket containing the bound SAH (Figures 2B and 2C). The residues K47 and R279 serve to constrict the space within this groove, while R74 overlooks the ridge that surrounds the SAH-binding pocket. Crucially, R82 and R282, that are centrally located close to the SAH molecule itself, coordinate one of the negatively charged sulfate ions (SO_4_^2-^) that we found in the ΔN structure (Figures 1D and 2C), potentially mimicking how an RNA substrate might position itself on the enzyme.

To directly examine the role of these N-terminal residues in RNA-binding and hence catalytic activity, we individually converted positively charged residues to neutral alanine. Interestingly, mutant METTL16-core versions carrying the single point mutations K5A, R10A, R12A, K14A, and K16A did not affect RNA methylation activity (Figures 2D and S2D). However, individual mutations into a negatively charged residue (K5E, R10E, and R10D) had a more discernible impact by reducing RNA methylation activity (Figure 2D). These individual mutations did not completely abolish activity as seen in the catalytic-dead mutant PP185-186AA, indicating that these might merely reduce RNA binding. Strikingly, a combined mutant with all five residues converted to alanine (MUT1: K5A, R10A, R12A, K14A, and K16A) completely eliminated RNA methylation activity (Figure 2E). In contrast, a combined mutation of charged residues not lining the potential RNA-binding groove (MUT2: K26A and K31A) (Figure 2B) did not affect RNA methylation activity (Figure 2E). Consistently, a further mutant (MUT3) containing all the mutations made in MUT1 and MUT2 did not show any activity. To examine RNA binding, we performed UV crosslinking experiments (Figure 2F). As expected, the full-length METTL16 and the METTL16-core version bound the 5′ end-labeled RNA. In contrast, the METTL16-core version carrying the combined MUT1 mutations showed highly reduced binding. These results provide a structural rationale for absence of RNA binding and RNA methylation activity in the METTL16-ΔN protein.

We extended the mutational analyses to the other positively charged residues lining the putative RNA-binding groove. Mutation of the residues K47 and R279 that form a claw-like constriction of the groove either reduces (in the case of K47E) or abolishes (in R279E or double mutant K47E+R279E) methylation activity (Figure S2E). Mutation of other residues R82E, R282E, and R74E also abolishes activity, confirming their involvement in construction of the putative RNA-binding groove.

Next, we probed the importance of the disordered loop containing the catalytic residues (Figure 2G). Confirming its critical role, deletion of most of the loop (190–218 aa) abolishes *in vitro* methylation. In fact, loss of methylation can be reproduced by just three point mutations (Loop-3R-E: RRR-200-203-204-EEE) converting positive charges to negative residues, while mutation of four prolines (Loop-4P-A) within the loop did not affect methylation activity (Figure 2G). Interestingly, deletion of the disordered loop did not have an adverse effect on RNA binding, as measured by UV crosslinking (Figure 2F). However, the Loop3R-E mutant displayed highly reduced RNA binding, probably due to repulsion of RNA. These results indicate that the loop, per se, is not required for RNA binding, but has a potential catalytic role by directly contacting the RNA for proper positioning within the catalytic pocket.

In conclusion, our structure-informed mutagenesis study traces an RNA-binding groove lined by positively charged residues contributed by the N-terminal module and the MTase domain itself. This facilitates RNA binding, and thus promotes RNA methylation activity. In addition, we identify a disordered loop that is essential for catalysis.

### METTL16 Prefers Structured RNAs as Substrates for m^6^A Methylation *In Vitro*

**Figure 3:**
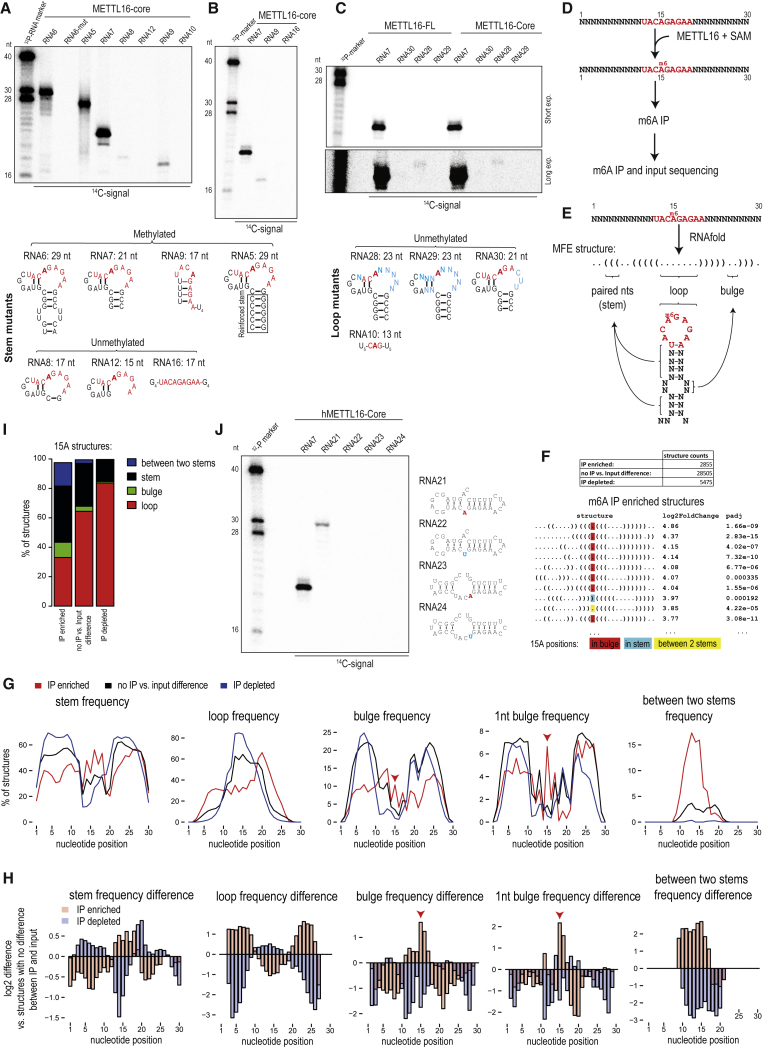
METTL16 Requires Structured RNA for m^6^A Methylation (A) *In vitro* methylation assay with RNAs carrying truncations of the stem region. See also Figure S3A. The predicted structures of RNAs used are shown below. (B) *In vitro* methylation assay. (C) *In vitro* methylation assay with RNAs carrying mutations in the nonamer consensus sequence (shown below). Short and long exposures of the gel are shown. (D) Scheme of an *in vitro* methylation experiment using a library of randomized (N = any of the four nucleotides) RNA oligos. (E) For each sequence, we predicted the minimum free energy (MFE) secondary structure using RNAfold (STAR Methods). A model structure is shown in dot bracket notation. (F) The representation of individual structures (corresponding to unique dot bracket notation) was compared between m^6^A-IP samples and input samples. Top ten IP-enriched structures are shown. The 15th position adenosine (A) that is in the consensus nonamer sequence is highlighted. (G) Frequency of structures forming stem, loop and other selected features at individual positions is shown. The IP-enriched structures have increased frequency of 15A (red arrowhead) in a bulge and surrounded by double-stranded regions (stems), pointing to specific structural requirements of RNA substrates for METTL16. (H) Structures enriched or depleted in m^6^A IP were compared to those that do not show such difference (between IP and input). While the IP-enriched structures have higher proportion of 15A (red arrowhead) forming a bulge or lying between two stems, the IP-depleted structures show the opposite trend, with lack of structures with 15A in a bulge or in between two stems. (I) The barplot shows the proportion of structural features in which the 15A was found. Note the high proportion of structures with 15A in the bulge and between two stems, among the m^6^A IP-enriched structures. See also Figure S3. (J) *In vitro* methylation assay with METTL16-core protein and RNAs (RNA21 and RNA23) selected from randomized library methylation experiment (D). This confirms the specific methylation of 15A which is in a 1 nt bulge (in RNA21, but not in mutant RNA22).

The two known methylation targets of METTL16 are structured RNAs: the U6 snRNA and *MAT2A* hp 1 (Pendleton et al., 2017, Warda et al., 2017). To identify the RNA features that can allow *in vitro* methylation by METTL16, we carried out truncations/mutations of the *MAT2A* hp1. The *MAT2A* hp RNAs with reductions in the stem region beyond three base pairs fail to get methylated (Figures 3A, S2B, S3A, and S3B) (Pendleton et al., 2017). Activity can be restored by an artificial six base pair C/G stem (RNA5), indicating the critical requirement for any stem region (Figures 3A and S1D). On the other hand, presence of the nonamer motif in a single-stranded context, when flanked by a run of Gs (RNA16), did not support methylation (Figure 3B). Interestingly, an RNA where the nonamer motif is flanked by a run of Us (RNA9) is a substrate (albeit weak) for METTL16, likely because it has the potential to form a structured feature (Figures 3A, 3B, and S3B). To test the importance of the nonamer itself, we introduced three mutations within this motif (RNA30), which abolished activity (Figure 3C). Additional mutations, in the form of randomizations of the motif sequences (RNA28 and RNA29), also abolished activity (Figure 3C). Lastly, an RNA with only the central C**A**G flanked by Us (RNA10) was inactive, reaffirming the importance of this nonamer motif for methylation (Figure 3A). Taken together, these studies indicate that the nonamer sequence in the context of a secondary structure feature is essential for METTL16 methylation activity *in vitro*.

To probe the secondary structure requirements in an unbiased manner, we carried out *in vitro* methylation reactions with recombinant full-length METTL16 and a library of randomized 30 nt single-stranded RNAs carrying a central nonamer motif (Figure 3D). Subsequently, a part was retained as input while the rest was used for immunoprecipitation (IP) of methylated RNAs with the anti-m^6^A antibody (Figure 3D). Sequences were identified by deep sequencing and preferred secondary structures of these sequences were examined using RNAfold (STAR Methods; Table S2) (Figure 3E). We identified ∼2,800 predicted structures (referring to unique dot bracket notations) to be enriched in the m^6^A-IP library (Figure 3F).

To find out whether specific structural features are important for methylation, we focused on selected secondary structural features (i.e., stems, loops, bulges, and nucleotides lying between two stems) and compared the frequencies of these features for individual nucleotide positions between the m^6^A-enriched, non-enriched, and m^6^A-depleted structures (Figures 3G and 3H), or directly between the sequenced oligos (Figures S3C and S3D). Analysis indicates that the nonamer sequence motif of m^6^A-enriched structures occurs with a higher frequency in a paired stem region. However, the 15th nt adenosine that is expected to be methylated within the motif is often not paired and more frequently present in a 1 nt bulge. There is also a higher frequency for the unpaired 15A to lie between two stems (Figures 3G–3I). m^6^A IP-enriched and IP-depleted sequences show an opposing trend affirming the importance of an unpaired adenosine surrounded by local double-strands for methylation (Figure 3H). Indeed, an RNA (RNA 21) representative of the structure enriched in the m^6^A-IP (15th position adenosine in a bulge between two stems) supports methylation by METTL16 (Figure 3J). This is specific as methylation is abolished when the 15th position A is mutated to U (RNA22) (Figure 3J). However, another RNA (RNA23) did not show any methylation activity (Figure 3J). These results show that the nonamer motif does not necessarily have to adopt a loop structure for activity but that the target adenosine must be unpaired and surrounded by stems. Taken together, we reveal that besides the sequence motif there is a structural requirement for a nonamer to serve as a substrate for METTL16 methylation.

### Mouse METTL16 Regulates Embryonic *Mat2a* mRNA Levels and This Is Essential for Embryonic Development

**Figure 4:**
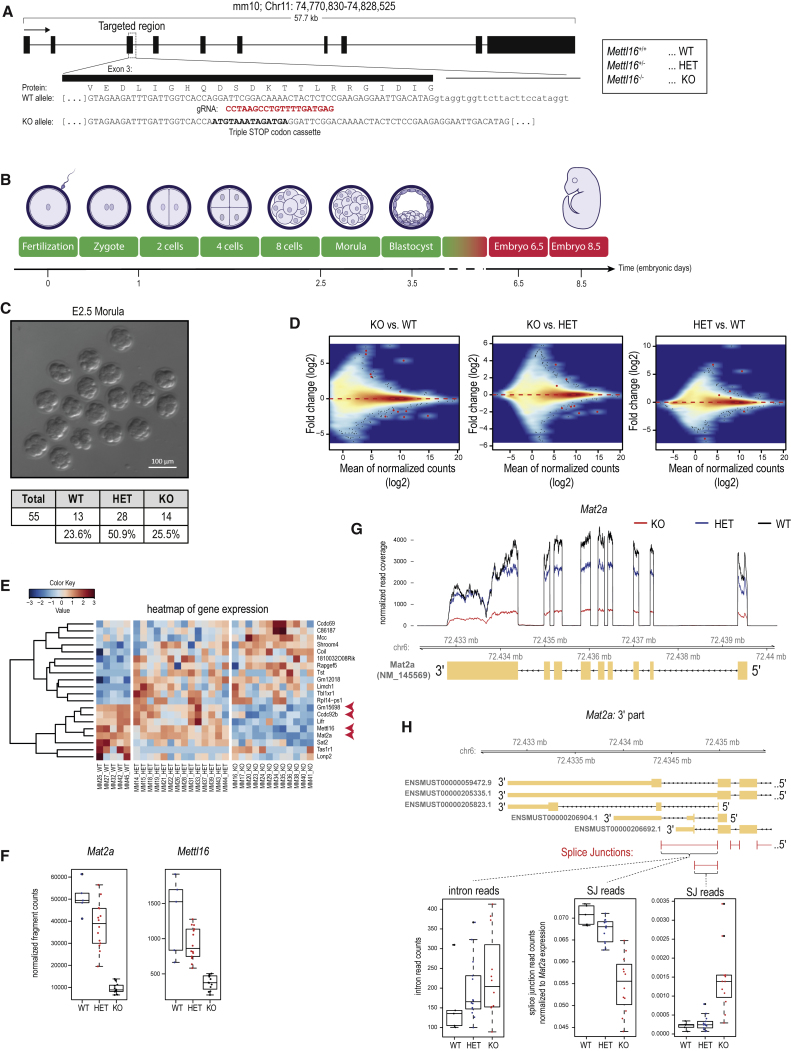
Reduced *Mat2a* mRNA Levels and Embryonic Lethality around Implantation Stage in the *Mettl16* Mutant Mice (A) Generation of a *Mettl16* knockout (KO) allele. See also Figure S4A and STAR Methods. (B) Timeline of mouse embryogenesis. Embryonic day 2.5 (E2.5) embryos referring to 16-cell morula stage, E3.5 blastocysts, and E6.5 and E8.5 embryos were collected for genotyping. KO embryos were detected in expected Mendelian ratios till E3.5 (colored in green), but at sub-Mendelian ratios at E6.5 or none beyond (colored in red). See also Figures S4C–S4F. (C) Genotyping of E2.5 embryos from *Mettl16*^+/−^ x *Mettl16*^+/−^ crosses confirmed the expected Mendelian ratios among the genotypes. Scale bar in μm is indicated. (D) Transcriptome of individual isolated E2.5 embryos of *Mettl16*^−/−^ (KO), *Mettl16*^+/−^ (HET), and *Mettl16*^+/+^ (WT) was sequenced and compared between the genotypes. The MA plots show a very limited number of differentially expressed genes (red dots, adjusted p ≤ 0.1). See also Figure S5. (E) Heatmap shows the expression of genes with significant differential expression between any two genotypes (adjusted p ≤ 0.1). Genes differentially expressed in *Mettl16*^−/−^(KO) when compared to both *Mettl16*^+/−^ (HET) and *Mettl16*^+/+^ (WT) are marked by red arrowhead. (F) The boxplots show the expected downregulation of the targeted gene (*Mettl16*) in KO samples, as well as the downregulation of *Mat2a*. Transcript levels of individual samples are shown as dots. See also Figure S5C. (G) Normalized read coverage along the *Mat2a* locus demonstrates the overall depletion in the KO. Note that the gene is on the Crick strand, so it goes from right to left. (H) Lack of METTL16 results in aberrant splicing of the last intron. The reads spanning the splice junction (SJ) of last *Mat2a* (ENSMUST00000059472.9) intron are significantly depleted in the KO even when normalized to overall *Mat2a* transcript levels. This is accompanied by slight increase for intron reads and increased usage of alternative 3ʹ splice-site characteristic for the ENSMUST00000206904.1 and ENSMUST00000206692.1 variants. See also Figure S5D.

To uncover the endogenous targets of METTL16, we created a knockout allele (*Mettl16*^*-*^) by inserting a triple-stop codon cassette into exon 3 of the mouse *Mettl16* genomic locus (Figures 4A, S4A, and S4B; STAR Methods). Heterozygous (HET) *Mettl16*^*+/−*^ mice of both sexes are viable and fertile. Intercrosses between them provided litters that were completely devoid of any homozygous (KO) *Mettl16*^*−/−*^ animals, indicating potential embryonic lethality (Figure S4C).

**Figure 5:**
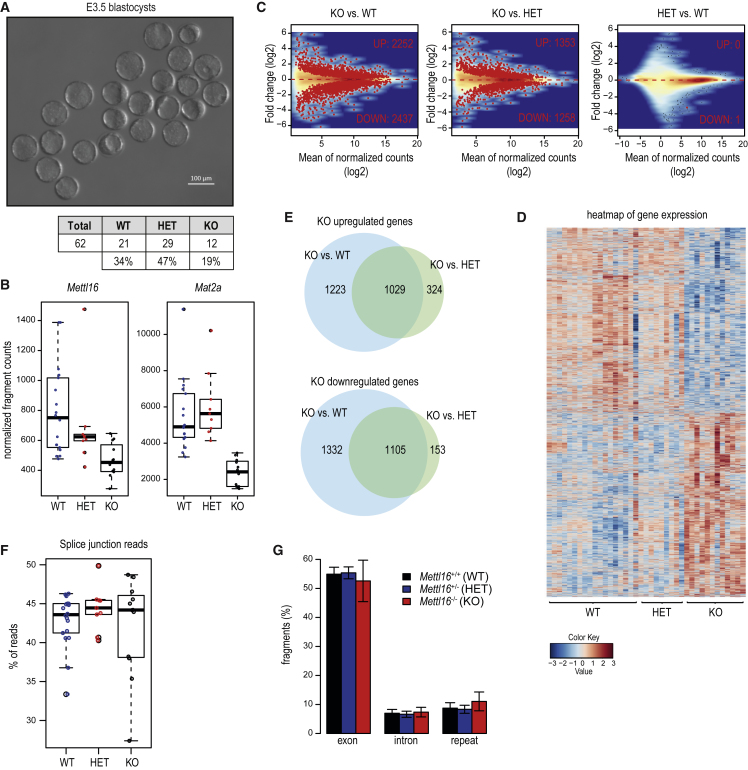
E3.5 *Mettl16*^−/−^ Blastocysts Display Normal Morphology but Vast Transcriptome Dysregulation (A) E3.5 *Mettl16*^−/−^ KO embryos display normal morphology and their counts from *Mettl16*^+/−^ x *Mettl16*^+/−^ crosses correspond to expected Mendelian ratios among the genotypes. Scale bar in μm is indicated. (B) The boxplots show the expected downregulation of the targeted gene (*Mettl16*) in KO samples, as well as the downregulation of *Mat2a*. Transcript levels of individual samples are shown as dots. See also Figure S6A. (C) MA plots comparing the expression between the genotypes reveal that the vast number of genes are dysregulated in the *Mettl16*^−/−^ KO embryos. The genes with significantly different expression are shown as red dots (adjusted p ≤ 0.1). (D) Heatmap shows the expression of 5,166 genes with significant differential expression between any two genotypes (adjusted p ≤ 0.1). Note the massive dysregulation in the KO embryos. See also Figures S6B–S6D. (E) Venn diagrams compare the lists of dysregulated genes when *Mettl16*^−/−^ expression is compared to *Mettl16*^+/−^ or to *Mettl16*^+/+^. (F) Comparison of proportion of reads encompassing splice junctions does not reveal a difference in splicing between individual genotypes. (G) Global transcription from exons, introns, and repeats is not affected in *Mettl16*^−/−^. Error bars refer to SD.

After fertilization of the oocyte by the sperm, the 1-cell zygote goes through mitotic divisions to take it through a totipotent 2-cell stage, followed later by the pluripotent 16-cell morula seen at embryonic day 2.5 (E2.5) and 32- to 64-cell blastocyst at E3.5. Subsequently, the embryo becomes implanted in the uterine wall and proceeds into post-implantation development (Figure 4B). To examine the embryonic arrest in the *Mettl16* mutant, we first isolated E2.5 morula from superovulated *Mettl16*^*+/−*^ females crossed with *Mettl16*^*+/−*^ males (Figure 4C). Visual examination revealed no apparent differences in the embryos at this stage. Examination of E3.5 blastocysts resulted in a similar conclusion (Figure 5A). Indeed, genotyping of individual embryos confirmed the presence of all genotypes in the expected Mendelian ratios at both E2.5 and E3.5 (Figures 4C and 5A). However, examination of post-implantation embryos at E8.5 and E12.5 indicated a total absence of the knockout genotype, but E6.5 KO embryos (1.9%) were detected at sub-Mendelian ratios (Figures S4D–S4F). These results indicate that the *Mettl16*^*−/−*^ knockout mutation allows embryonic development until blastocyst stage but causes developmental arrest around the time of implantation.

To evaluate the impact of loss of METTL16, we sequenced the transcriptomes of individual 16-cell morulas at E2.5 originating from heterozygous *Mettl16*^*+/−*^ crosses. Embryos were genotyped based on presence or absence of specific *Mettl16* reads (STAR Methods), and gene expression levels were compared between different genotypes (Figure 4D). We find 20 genes to be differentially expressed between the different genotypes (WT, HET, and KO) (Figure 4E; Table S3). However, only four genes are consistently different in the KO embryos when compared to both WT and HET (marked with red arrowheads in Figures 4E and S5B). Examination of transcript changes in the individual embryos reveals an expected and consistent downregulation of *Mettl16* in the KO embryos (Figure 4F). Strikingly, the most significantly dysregulated transcript was *Mat2a*, which displays a 5-fold downregulation in the KO embryos. Two additional transcripts *Ccdc92b* and *Gm15698* also display significant downregulation in the KO embryos (Figure S5C).

Identification of *Mat2a* as a downregulated transcript in the *Mettl16* knockout embryos is interesting, as it is already an established target of METTL16 in human cell lines (Pendleton et al., 2017). METTL16 was proposed to promote splicing of the terminal intron, failure of which leads to intron-retention and transcript degradation (Pendleton et al., 2017). Examination of the read count distribution over the exons and introns of *Mat2a* reveals that while the exonic reads are consistently decreased in the KO, we did not observe any dramatic change in intronic reads (Figures 4G and S5D). The same was true for the two other transcripts downregulated in the KO embryos (Figure S5E). Nevertheless, a closer examination around the terminal intron of the *Mat2a* indicates a differential usage of splice junctions in the KO accompanied by a slight increase in the terminal intronic reads (Figure 4H). In conclusion, we demonstrate that *Mettl16* is essential for viability of early mouse embryos where it regulates the levels of *Mat2a* mRNA.

### Loss of METTL16 Leads to Dramatic Alterations in the E3.5 Blastocyst Transcriptome

To examine whether the downregulation of very few transcripts in E2.5 embryos has further consequences in the E3.5 blastocysts, we collected such embryos from superovulated *Mettl16*^*+/−*^ females crossed with *Mettl16*^*+/−*^ males (Figure 5A). Sequencing of single embryos revealed the expected downregulation of *Mettl16* and *Mat2a* (Figure 5B). Strikingly, ∼5,000 other transcripts were either upregulated or downregulated in the KO, when compared to the WT embryos, while up to half that number was altered in the KO versus HET comparison (Figures 5C, 5D, and S6D). Examination of these altered-gene lists indicates that up to 1,000 genes are either commonly up- or downregulated in the KO when compared to both WT and HET embryos (Figure 5E). A previous study identified key transcription and chromatin factors that define specific developmental stage transcriptomes (Mohammed et al., 2017). Examination of these factors in our datasets did not reveal any altered expression profile between the genotypes (Figures S6B and S6C). A Gene Ontology (GO) term analysis of the altered transcripts revealed an upregulation in splicing-related factors (Table S3), but analysis of splice junction reads did not reveal any changes in the KO embryos (Figure 5F). We also did not observe any dramatic changes in the representation of exon, intron, and repeat reads in the different libraries (Figure 5G). Taken together, even though the molecular effect of the loss of METTL16 is already seen in E2.5 embryos in the form of reduced mRNA levels of its methylation target *Mat2a*, its consequences are amplified in the E3.5 KO embryos. Here, a massive dysregulation of gene expression is observed, such that mutant embryos undergoing implantation are doomed to fail in further development.

## Discussion

Crystal structures now reveal how two RNA methyltransferases are built to recognize distinct RNA targets and install the same m^6^A mark. The two methyltransferase (MTase) domains in the heterodimeric METTL3/METTL14 complex interact to create a narrow groove lined with conserved positively charged residues into which single-stranded RNAs can fit (Śledź and Jinek, 2016, Wang et al., 2016a, Wang et al., 2016b). This interaction facilitates stabilization of a large “interface loop” in METTL3 that contributes to the catalytic activity. Indeed, this ensures that METTL3, which binds SAM, is not active on its own, requiring at least the MTase domain of METTL14 to complete the creation of a functional catalytic complex (Śledź and Jinek, 2016, Wang et al., 2016a). In contrast, we show here that METTL16 is active as a monomer (Figures 1 and S1B), and it contains a large deep-cut groove that can accommodate structured RNAs (Figure 2). Interestingly, the METTL3/METTL14 crystal complex with the two MTase domains is inactive and requires the two N-terminal CCCH zinc finger motifs of METTL3 to recover methylation activity (Śledź and Jinek, 2016, Wang et al., 2016a), presumably because it aids in substrate RNA binding. Similarly, here we demonstrate that the N-terminal module attached to the MTase of METTL16 is essential for RNA-binding and catalysis (Figures 1 and 2). We note that the recently reported crystal structure of the human METTL16 core MTase domain (PDB 6B92) (Ruszkowska et al., 2018) shows a high degree of overlap with the one studied here (Figure S1F; Star Methods).

**Figure 6:**
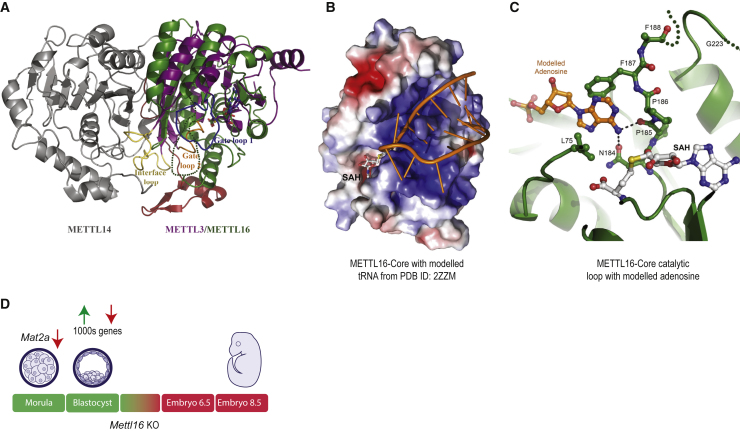
A Model for METTL16 Function during Early Embryonic Development (A) Structural comparison of METTL16 core and METTL3/METTL14 complex. METTL3 (PDB 5IL2), colored purple, was superimposed on the METTL16-core-SAH (PDB 6GFN), colored in green (core) and red (N-terminal). Gate loops 1 and 2, and the interface loop of METTL3, are colored in blue, orange, and yellow, respectively. The disordered loop in our METTL16-core is shown as a dotted line. (B) Surface charge representation of human METTL16-core domain with a modeled tRNA (from PDB: 2ZZM). See STAR Methods. The SAH bound in the catalytic pocket is shown. (C) A methyl-acceptor adenosine (orange) was modeled into the SAH binding site of METTL16 core (PDB 6GFN) by superimposition of an m^6^A DNA MTase, EcoP151 (PDB: 4ZCF, chain B). The sulfate binding site (as in Figure 1F) overlaps with the adenosine base moiety. (D) A model summarizing the physiological role of METTL16 during early mouse development. The downregulation of the SAM synthetase *Mat2a* mRNA in *Mettl16* KO E2.5 morula is potentially a trigger for subsequent massive alteration in gene expression in the E3.5 blastocysts. Such mutant embryos fail to proceed further in development (indicated in red).

A structural comparison of the human METTL3/METTL14 complex (PDB 5IL2) with that of our human METTL16-core (PDB 6GFN) reveals similarity to METTL3 in the overall Rossmann fold (Figure 6A). It also shows how the disordered loop in METTL16 (Figures 1E and 2G) is very similar to the “gate loop 1” in METTL3, as both harbor the catalytic residues and are likely involved in contacting the bound RNA during enzymatic reaction. Our mutational studies indicate that the disordered loop in METTL16 is not required for RNA binding (Loop-Del in Figure 2F) but is essential for catalytic activity (Figure 2G). Thus, its role might be to contact the substrate bound via the RNA-binding groove and orient it for catalysis. This is supported by our finding that mutation of positively charged arginine (R) residues in the loop to glutamic acid (E) abolishes RNA binding (Figure 2F), perhaps via charge repulsions. We modeled a structured RNA (tRNA from PDB 2ZZM) into this groove, and it shows how an unpaired adenosine in the loop region might reach into the catalytic pocket for methylation (Figure 6B). To get better insight into the catalytic mechanism, we modeled a methyl-acceptor adenosine (from PDB 4ZCF, chain B) (Gupta et al., 2015) into the SAH binding site of METTL16-core (PDB 6GFN) (Figure 6C). Superimposition of the METTL16-core structure with that of the m^6^A DNA MTase, EcoP1Gl (PDB 4ZCF, chain B), reveals how the adenosine is favorably positioned by coordination with catalytic residues N184 and P185 for the methyl transfer from SAM (represented by SAH in Figure 6C). However, our experiments do not reveal how METTL16 might be able to recognize an adenosine within a specific nonamer sequence for m^6^A methylation. This information will be forthcoming only when structures with bound RNA become available.

Regulation of gene expression by m^6^A is essential at multiple steps during mouse embryonic development. The writer *Mettl3* is essential for embryonic development, with *Mettl3*-deficient embryonic stem cells (ESCs) failing to exit pluripotency despite differentiation cues (Batista et al., 2014, Geula et al., 2015, Wang et al., 2014). Now we show that the writer *Mettl16* is also essential for embryonic development around implantation stage (Figures 4 and 5). Our biochemical studies and *in vivo* transcriptome profiling reveals severe sequence and structural constraints on potential RNA targets of METTL16 (Figures 3 and 4). Although a few hundred transcripts carrying the nonamer sequence motif exist in the mouse genome, we did not find any differences in their levels in *Mettl16* knockout E2.5 embryos (Figure 4). This reinforces our finding that a combination of sequence and structural features define the target set for METTL16. The fact that *Mat2a* is the sole main target of METTL16 in pre-implantation embryos is interesting, as it encodes for the SAM synthetase, which produces SAM, the main methyl donor required for many methylation reactions (including DNA, protein, and RNA methylation) with huge regulatory potential. Before implantation, the embryonic genome undergoes massive erasure of DNA methylation marks, while during post-implantation development, DNA methylation increases and is restored back to normal levels (Reik et al., 2001). Given our finding that the transcriptome in the E3.5 embryos is massively dysregulated (Figure 5), we propose that it is a snowballing effect of the initial downregulation of *Mat2a*. The low levels of *Mat2a* mRNA will mean that downstream epigenetic reprogramming events are also bound to fail. Such E3.5 mutant blastocysts are unfit for continuing in development (Figure 6D). In this context, it is interesting to point out that a homozygous *Mat2a* knockout mutation results in embryonic lethality in mice (International Mouse Phenotyping Consortium [IMPC]). Furthermore, chemical inhibition of bovine MAT2A enzyme in cultured bovine pre-implantation embryos also reduced blastocyst development (Ikeda et al., 2017).

How might METTL16 function to stabilize *Mat2a* mRNA in the mouse embryos? METTL16-mediated methylation of the hairpin structures in the 3ʹ UTR in *Mat2a* mRNA is used by YTHDC1 to promote its decay in high-SAM conditions (Shima et al., 2017). Thus in the absence of *Mettl16* we would have expected a stabilization of the transcript. Perhaps an explanation might come from the proposed non-canonical function of METTL16 as a splicing enhancer (Pendleton et al., 2017), where it promotes splicing to remove the 3ʹ terminal intron to create a stable mature *MAT2A* mRNA during low-SAM conditions. Based on this model, loss of *Mettl16* leads to reduced splicing of the terminal exon, resulting in intron-retained unstable transcripts and hence not detected in our sequencing experiments (Figures 4 and 5). Consistently, we detected higher levels of intronic reads in the *Mettl16* knockout embryos (Figure 4H). The C-terminal vertebrate conserved regions (VCRs) of METTL16 are proposed to mediate this activity (Pendleton et al., 2017). We speculate that some of the splicing factors we identified in endogenous METTL16 complexes from mouse tissues, and from transfected human cell lines, may participate in this role (Figures S5F and S5G). Future studies using a catalytic-dead METTL16 mutant mouse should help settle this issue of methylation-mediated decay versus splicing role. However, it is also possible that multiple pathways co-exist to control *Mat2a* levels.

HeLa cell extracts were originally shown to harbor an activity that adds an m^6^A mark at position A43 in the splicing machinery component U6 snRNA (Shimba et al., 1995). This activity was later identified to be METTL16 (Pendleton et al., 2017, Warda et al., 2017). Mutation of this methylation site in yeast U6 snRNA, which lies within a region that base pairs with the 5ʹ splice site of pre-mRNAs, causes lethality (Madhani et al., 1990). However, our analysis of the *Mettl16* knockout mouse embryos did not reveal any gross changes in splicing patterns across the transcriptome (Figures S5A and 5G). Thus, it is possible that methylation of U6 snRNA is not critical for splicing in higher organisms, perhaps due to the diversity of 5ʹ splice site sequences (Yang et al., 2013). Alternatively, complementation by another MTase activity in our mutant might account for lack of splicing defects. In conclusion, our studies place the m^6^A writer METTL16 in a dominant position to influence early developmental decisions in the mouse embryo via regulation of SAM synthetase expression.

## STAR★Methods

### Key Resources Table

**Table:** 

REAGENT or RESOURCE	SOURCE	IDENTIFIER
**Antibodies**		

Polyclonal rabbit anti-m^6^A	Synaptic Systems	Cat. no. 202003; RRID:AB_2279214
Polyclonal rabbit anti-METT10D	Abcam	Cat. no. ab186012
Mouse IgG control antibody	Santa Cruz	Cat. no. sc-2025; RRID:AB_737182

**Bacterial and Virus Strains**		

DH10EMBacY bacterial strain	(Bieniossek et al., 2012)	N/A

**Biological Samples**		

PMSG	MSD Animal Health	Folligon
HCG	MSD Animal Health	Chorulon

**Chemicals, Peptides, and Recombinant Proteins**		

Sodium deoxycholate	Sigma	30968
Complete EDTA-free protease inhibitor	Roche	11 873 580 001
^14^C-S-ADENOSYL-L-METHIONINE	Perkin Elmer	NEC363010UC
N^6^-methyl adenosine	Sigma-Aldrich	M2780
Anti-HA Affinity Matrix	Roche	Cat. no. 11815016001; RRID:AB_390914

**Critical Commercial Assays**		

NEBNext Multiplex Small RNA Library Prep Set for Illumina	NEB	E7300
MinElute Gel Extraction Kit	QIAGEN	28604
MEGAshortscript T7 Transcription Kit	Life technologies	Cat. no. AM1354
Dynabeads Protein A	Life Technologies	10002D

**Deposited Data**		

Deep sequencing datasets	This study	GEO accession: GSE116329
All raw gel data are deposited at Mendeley Data.	This study	https://doi.org/10.17632/ny82j2ngt5.1
Structure: METTL16-core, crystal form 1	This study	PDB ID: 6GFN
Diffraction images: METTL16-core, form 1	This study	DOI:10.15785/SBGRID/578
Structure: METTL16-core, crystal form 2	This study	PDB ID: 6GT5
Diffraction images: METTL16-core, form 2	This study	DOI:10.15785/SBGRID/579
Structure: METTL16-DN	This study	PDB ID: 6GFK
Diffraction images: METTL16-DN	This study	DOI:10.15785/SBGRID/577

**Experimental Models: Cell Lines**		

Sf21 insect cells for protein production	Eukaryotic Expression Facility, EMBL Grenoble, France	N/A
High Five insect cells for protein production	Eukaryotic Expression Facility, EMBL Grenoble, France	N/A

**Experimental Models: Organisms/Strains**		

Mouse: *Mettl16 knock-out*	This study	Available from Lead Contact

**Oligonucleotides**		

DNA and RNA oligos		See Table S1

**Recombinant DNA**		

pACEBac2	Bieniossek et al., 2012	N/A
Human *Mettl16* cDNA	This study	NP_076991; NM_024086

**Software and Algorithms**		

Cutadapt		http://journal.embnet.org/index.php/embnetjournal/article/view/200
ENRICHR	Chen et al., 2013, Kuleshov et al., 2016	http://amp.pharm.mssm.edu/Enrichr/
MEME - Motif discovery tool	Bailey and Elkan, 1994	
R	R Core Team, 2017	https://www.r-project.org
DESeq2	Love et al., 2014	N/A
Bioconductor	Huber et al., 2015	https://www.bioconductor.org/
Gviz	Hahne and Ivanek, 2016	N/A
STAR	Dobin et al., 2013	N/A
Salmon	Patro et al., 2017	N/A
tximport	Soneson et al., 2015	N/A
featureCounts	Liao et al., 2014	N/A
JunctionSeq	Hartley and Mullikin, 2016	N/A
RNAfold	Lorenz et al., 2011	N/A
Phaser	McCoy et al., 2007	http://www.phaser.cimr.cam.ac.uk/index.php/Phaser_Crystallographic_Software
XDS suite	Kabsch, 2010	http://xds.mpimf-heidelberg.mpg.de
autoPROC	Vonrhein et al., 2011	http://www.globalphasing.com/autoproc/
STARANISO	Tickle et al., 2017	http://staraniso.globalphasing.org/cgi-bin/staraniso.cgi
Coot	Emsley et al., 2010	http://www2.mrc-lmb.cam.ac.uk/personal/pemsley/coot
BUSTER	Bricogne et al., 2016	http://www.globalphasing.com/buster
MOLPROBITY	Chen et al., 2010	http://molprobity.biochem.duke.edu
PyMOL	Molecular Graphics System, Version 1.8.6 Schrodinger, LLC	https://pymol.org/2/
PDB2PQR	Dolinsky et al., 2004	http://nbcr-222.ucsd.edu/pdb2pqr_2.0.0/
SBgrid	Morin et al., 2013	https://sbgrid.org/

**Other**		

Chelating Sepharose Fast Flow beads	GE Healthcare	17-0575-01
StrepTrap HP	GE Healthcare	28-9075-46
Superdex S75 10/300 GL	GE Healthcare	17-5174-01
Superdex 200 10/300 GL	GE Healthcare	17-5175-01
MethaPhor agarose	Lonza	50180

### Contact for Reagent and Resource Sharing

Further information and requests for resources and reagents should be directed to and will be fulfilled by the Lead Contact, Ramesh S. Pillai (ramesh.pillai@unige.ch).

### Experimental Model and Subject Details

#### Animal Work

Mutant mice were generated at the Transgenic Mouse Facility of University of Geneva. The mice were bred in the Animal Facility of Sciences III, University of Geneva. The use of animals in research at the University of Geneva is regulated by the Animal Welfare Federal Law (LPA 2005), the Animal Welfare Ordinance (OPAn 2008) and the Animal Experimentation Ordinance (OEXA 2010). The Swiss legislation respects the Directive 2010/63/EU of the European Union. Any project involving animals has to be approved by the Direction Générale de la Santé and the official ethics committee of the Canton of Geneva, performing a harm-benefit analysis of the project. Animals are treated with respect based on the 3Rs principle in the animal care facility of the University of Geneva. We use the lowest number of animals needed to conduct our specific research project. Discomfort, distress, pain and injury is limited to what is indispensable and anesthesia and analgesia is provided when necessary. Daily care and maintenance are ensured by fully trained and certified staff. This particular work was approved by the Canton of Geneva (GE/6/18).

#### *Mettl16* knockout mice

The *Mettl16* gene locus is located on mouse chromosome 11 and consists of 10 exons (Figure S4A). We targeted the endogenous *Mettl16* locus in mouse embryos of the B6D2F1/J hybrid line (also called B6D2; The Jackson Laboratory, stock no. 100006). It is a cross between C57BL/6J (B6) and DBA/2J (D2), and heterozygous for all B6 and D2 alleles. Single-cell mouse embryos were injected with a guide RNA (gRNA) that directs the DNA endonuclease Cas9, and a 170 nt single-stranded DNA (ssDNA) repair template (IDT). The ssDNA carries a triple-stop codon flanked by a 81 nt 5ʹ homology arm and a 75 nt 3ʹ homology arm. Founder mice were identified by genotyping PCR (Figure S4B) and crossed with wild-type C57BL/6JRj (Janvier) partners to obtain germline transmission. We obtained two lines: line #2112 where the homology recombination template was inserted, resulting in a triple-stop codon cassette (sequence: ATGTAAATAGATGA) in exon 3, and line #2175 where a 7 bp deletion led to removal of a splicing donor site in intron 4. It is expected that creation of premature termination codons in both lines should result in removal of the transcripts via nonsense-mediated decay (NMD). Heterozygous *Mettl16*^*+/−*^ mice of both sexes were viable and fertile, while homozygous mutants were not recovered in born litters (Figure S4C). Indeed, our analysis indicates that homozygous *Mettl16*^*−/−*^ mutation results in embryonic lethality around implantation (Figures 4, 5, and S4D–S4F). Both the generated lines showed the embryonic lethality phenotype. We used the line #2112 (with the triple-stop codon cassette) for sequence analysis of early embryos.

*Preparation of gRNAs*: A cloning-free method was used to prepare DNA template for *in vitro* transcription of the chimeric crRNA-tracrRNA, termed single guide RNA (sgRNA or gRNA). Briefly, a common reverse primer (CRISPR sgR primer) and a gene specific forward primer (CRISPR F primer) with T7 promoter sequence was used to PCR amplify the single-stranded sgDNA template. Primer sequences are provided in Table S1.

Forward (F) primer design template:

5′-GAAATTAATACGACTCACTATAGGNNNNNNNNNNNNNNNNNNNNGTTTTAGAGCTAGAAATAGC-3′

N represent the gene-specific sequence.

The following components were mixed to prepare the PCR reaction: 20 μl 5X Phusion HF buffer, 67 μl ddH2O, 2 μl 10 mM dNTPs, 5 μl of 10 μM CRISPR F primer, 5 μl of 10 μM CRISPR sgR primer, and 1 μl Phusion DNA polymerase. The PCR reaction was set as follows: 98°C for 30 s, 35 cycles of [98°C for 10 s, 60°C for 30 s and 72°C for 15 s], 72°C for 10 min, and finally at 4°C to hold the reaction. The PCR product (∼110bp) was agarose gel-purified using mini-elute gel extraction kit (QIAGEN, cat. no. 28604). The purified DNA was used to produce gRNA by *in vitro* transcription via the T7 promoter. *In vitro* transcription was carried out with the MEGAshortscript T7 Transcription Kit (Life technologies; cat no. AM1354) for 4 hours at 37°C. Reactions were treated with DNase I to remove template DNA, phenol-chloroform extracted and precipitated with ethanol. Quality of the generated gRNA was verified by 1.2% agarose gel electrophoresis.

*Denaturing formaldehyde-agarose gel electrophoresis*: Quality of generated gRNAs were verified by 1.2% agarose-formaldehyde gel electrophoresis. Agarose gel was prepared by mixing 0.6 g agarose, 36.5 mL H_2_O, 5 mL of 10x MOPS buffer (0.2 M MOPS, 80 mM sodium acetate, 10 mM EDTA) and 8.5 mL of 37% formaldehyde. Approximately, 4 μg of RNA was dissolved in the 4xRNA loading buffer (50% formamide, 6.5% formaldehyde, MOPS buffer 1x, bromophenol blue 0.2%, ethidium bromide 50 μg/ml) and heated to 65°C for 10 min. RNA was loaded into the gel and run at 70V for approximately 90 minutes. Gel was imaged in the E-Box VX5 (Vilber Lourmat, France) imaging station.

*Preparation of injection mix*: We mixed 12.5 ng/μl of the gRNA with 12.5 ng/μl of the 170 nt ssDNA repair template (IDT), and 25 ng/μl of Cas9 mRNA (ThermoFischer Scientifique; A29378), in injection buffer (10 mM Tris pH 7.5, 1 mM EDTA, pH 8.0). Prepare aliquots of 20 μL and store at −80°C.

Sequence of ssDNA repair template used: The triple-stop codon sequence is highlighted (bold, italic).

ssDNA (negative-strand sequence)

AGTTGAGAATGCAAAACCTATGGAAGTAAGAACCACCTACCTATGTCAATTCCTCTTCGGAGAGTAGTTTTGTCCGAATCC***TCATCTATTTACAT***TGGTGACCAATCAAATCTTCTACCCAGTGAATATAGTTAAGTCTCAAGGGGACTGTGGGAATTAGTCTCTCCAAA

Injection of mouse embryos of the hybrid background B6D2F1/J (black coat color) was carried out at the Transgenic Mouse Core Facility, University Medical Centre (CMU), University of Geneva. The B6D2F1/J hybrid line (also called B6D2; The Jackson Laboratory, stock no. 100006) is a cross between C57BL/6J (B6) and DBA/2J (D2), and heterozygous for all B6 and D2 alleles. The NMRI (Naval Medical Research Institute) mice, which have a white coat color were used as foster mothers.

#### Genotyping

Ear punches of the weaned animals (21 days-old) were digested in 100 μl of buffer containing 10 mM NaOH, 0.1 mM EDTA for 120 min at 95°C. After centrifugation at 3000 rpm for 10 min, 50 μl of supernatant was transferred to a new tube containing 50 μl of TE buffer (20mM Tris-HCl, pH 8.0 and 0.1 mM EDTA). An aliquot of 2 μl of the digestion mix was used for PCR.

Primers to detect bands (Figure S4B) corresponding to the wild-type (344 bp, WT), the triple-stop codon knock-in (358 bp, 2112) and 7 bp deletion (337 bp, 2175) alleles were MMoligo109 and MMoligo110 (Table S1). Identity of the bands were confirmed by Sanger sequencing.

Reaction mix for 25 μl PCR reactions: 1 × Taq buffer (without MgCl_2_, ThermoFisher cat. no. B38), 2 mM MgCl_2_, 0.5 μl dNTPs mix (stock 10 mM), 0.5 μl primer mix (stock 10 nM each), 2.0 μl tail DNA (100-200 ng), 0.5 μl Taq Pol (EMBL Protein Expression Facility, Heidelberg), water to make 25 μl final volume. Reactions were run using the following conditions (94°C, 20 s; 60°C, 30 s; 72°C, 30 s) for 35 cycles. Reactions were examined by 2.5% agarose gel electrophoresis (Figure S4B).

#### Mouse embryos

Heterozygous *Mettl16*^*+/−*^ adult (8 weeks-old) females were superovulated by hormone injections for E2.5 and E3.5 embryo collections. Briefly, one intraperitoneal (IP) injection of five International Units (IU) per mouse (volume, 0.1ml) of pregnant mare serum gonadotropin (PMSG; Folligon, MSD Animal Health) was given two days before crossing with males (at day −2). A second IP injection of 5 IU/mouse (volume, 0.1ml) of human chorionic gonadotropin (HCG; Chorulon, MSD Animal Health) at day 0 was administered to the females. The females were mated with *Mettl16*^*+/−*^ males immediately after the injections and checked for plugs the day-after (E0.5). The females were sacrificed 2 or 3 days later (embryonic days E2.5 or E3.5) to collect embryos at 16-cell morula and ≤ 64-cell blastocyst stages, respectively.

For single-embryo transcriptome sequencing, the isolated E2.5 and E3.5 embryos were visually examined for viability and cell number, and transferred separately into single tubes of 0.2 mL thin-walled 8-tube PCR strips (Thermo, AB-0451). The tubes contained 2 μL of the following mix: 0.4% Triton X-100 (vol/vol) in H2O + 2U/μl SUPERase⋅ In RNase Inhibitor (20 U/μL; Thermo, AM2694). Embryos were stored at −80°C prior to processing for Smart-seq2 library preparation (Picelli et al., 2014).

For genotyping E2.5, E3.5 embryos, these were collected as above from superovulated heterozygous *Mettl16*^*+/−*^ females and placed individually into single tubes of 0.2 mL thin-walled 8-tube PCR strips with 10 μl of lysis buffer [GoTaq G2 DNA Polymerase buffer (Promega, M7841), 200 μg/ml Proteinase K]. Embryos were lysed for 1h at 55°C, and then Proteinase K was inactivated by heating to 96°C for 10 min. 5 μl of the mix was used for PCR. Reaction mix for 20 μl: 5 x GoTaq G2 DNA Polymerase Buffer, 200 μM dNTP mix, 250 μM primers, 0.25 μl GoTaq G2 DNA Polymerase, 5 μl DNA. Reactions for oligo pair MMoligo109 + MMoligo110 were run using the following conditions (94°C, 20 s; 60°C, 30 s; 72°C, 30 s) for 35 cycles. Reactions were examined by 2% agarose gel electrophoresis.

For genotyping E6.5, E8.5 and E12.5 embryos, these were collected from heterozygous *Mettl16*^*+/−*^ females without superovulation. *Mettl16*^*+/−*^ females were mated with *Mettl16*^*+/−*^ males and plugs were checked on the day-after (E0.5). Plugged animals were separated. The females were sacrificed 6, 8 or 12 days later, in the late afternoon (between 4 pm to 7 pm). After dissection, embryos were placed in 50 μl of RNA*later* Stabilization Solution (ThermoFisher, AM7020) and kept at −80°C until isolation. RNA and DNA were extracted simultaneously using DNeasy Blood and Tissue Kit (QIAGEN, 69504) and RNeasy Plus Micro Kit (QIAGEN, 74034). RNA was stored at −80°C. 2 μl of DNA was used for genotyping. Reaction mix for 20 μl: 5 x GoTaq G2 DNA Polymerase Buffer, 200 μM dNTP mix, 250 μM primers, 0.25 μl GoTaq G2 DNA Polymerase, 2 μl DNA. Reactions for oligo pair MMoligo109 + MMoligo110 were run using the following conditions (94°C, 20 s; 60°C, 30 s; 72°C, 30 s) for 35 cycles. Reactions were examined by 2% agarose gel electrophoresis. For embryos for which agarose gel electrophoresis was not conclusive, the PCR was repeated and reaction products were cloned into pCR 2.1 vector using The Original TA Cloning Kit (ThermoFisher, 45-0046). Positive clones were selected and sequenced by Sanger sequencing.

### Method Details

#### Clones and constructs

##### Constructs for mammalian cell expression

Coding sequence for full-length (FL) human METTL16 (hMETTL16; 562 aa; Accession number NP_076991) was amplified from human HeLa cell total RNA by reverse transcription-PCR (RT-PCR). A mammalian expression vector (pCI-neo vector backbone) allowing production of 3xFLAG-HA tagged proteins from a cytomegalovirus (CMV) promoter was used. Sequence of the tag: ATGGACTACAAAGACCATGACGGTGATTATAAAGATCATGATATCGATTACAAGGATGACGATGACAAGggcggcagcggcTACCCATATGATGTTCCAGATTACGCT.

##### Constructs for insect cell expression

For the production of FL proteins, we used Baculovirus-mediated expression in insect cells. The full-length (1-562 aa) human METTL16 (hMETTL16) was cloned into the pACEBac2-SUMO acceptor vector (Bieniossek et al., 2012) for expression as an N-terminal 6xHis-Strep-SUMO-TEV fusion in the insect cells. For co-expression of human METTL3 and METTL4, full-length coding sequence for human METTL3 (1-580 aa) was cloned into the NheI and SphI restriction sites of the modified acceptor vector pACEBac2 to express the recombinant proteins with N-terminal 6xHis-SUMO-StrepIII-TEV fusions. The full-length coding sequence for untagged hMETTL14 (1-456) was cloned into the donor vector pIDK between KpnI/XhoI restriction sites. The proteins were co-expressed by taking advantage of MultiBac system (Bieniossek et al., 2012) which allows the generation of multi-gene constructs via Cre-lox recombination. The acceptor and donor vectors were combined in Cre-mediated reaction in total volume of 20 μl where 2 μg of each vector was mixed with 2 μl of 10x Cre buffer and 1 μl of Cre recombinase (NEB, cat no. M0298S). The reaction was incubated at 37°C for 1 h. After that, 5 μl of Cre reaction was transformed to 100 μl of competent TOP10 cells and plated on LB agar with appropriate antibiotics. The clones were verified by restriction digestion of the isolated plasmid, as well as by PCR.

##### Constructs for bacterial expression

Constructs covering only the core methyltransferase domain of hMETTL16 (1-291 aa) or its point mutant/deletion versions were cloned into the bacterial expression vector (pETM-11-SUMO vector; EMBL Protein Expression and Purification Core Facility) as 6xHis-Strep-SUMO-TEV fusions. The following constructs were prepared:

METTL16-ΔN: 40-291 aa, N-terminal deletion version similar to that used in PDB ID: 2H00 [Structural Genomics Consortium (SGC)].

METTL16-core: 1-291 aa.

##### METTL16-core mutants

1. Single amino acid changes: K5A, R10A, R12A, K14A, K16A, K5E, K10E, K10D, K47E, R74E, R82E, F187G, R279E, R282E.

2. MUT1: five residues (K5, R10, R12, K14, and K16) mutated to As.

3. MUT2: two residues (K26 and K31) mutated to As.

4. MUT3: combination of MUT1 and MUT2 sites mutated to As.

5. PP185-186AA: two residues (P185 and P186) mutated to As.

6. Loop-4P-A: four residues (P202, P205, P206, P207) mutated to As.

7. Loop-3R-E: three residues (R200, R203, R204) mutated to Es.

8. Loop-del: deletion of disordered loop 190-218 aa and replaced with a linker GGGSGGGS.

9. Double mutations in the binding groove: two residues (K47 and R279) mutated to Es.

#### Antibodies

The polyclonal rabbit anti-m^6^A (Synaptic Systems; 202003), antibody for detecting mouse METTL16- polyclonal rabbit anti-METT10D (abcam, ab186012) and normal mouse IgG (Santa Cruz, sc-2025) antibodies were purchased. Anti-HA affinity matrix (Roche; cat. no. 11815016001) and Pierce HA Epitope Tag Antibody (ThermoFisher, cat.no. #26181) were used for immunoprecipitations.

#### Recombinant protein production

Production of full-length recombinant proteins was carried out in insect cell lines using the baculovirus expression system. The ovary-derived cell lines used are: High Five (Hi5) insect cell line originating from the cabbage looper (*Trichoplusia ni*) and the Sf9 cells derived from the fall army worm *Spodoptera frugiperda*. Briefly, recombinant full-length hMETTL16 coding sequence was cloned into pACEBac2-Sumo acceptor vector (His-Strep-Sumo tag) (Bieniossek et al., 2012). Plasmids were transformed into DH10EMBacY competent cells for recombination with the baculovirus genomic DNA (bacmid). The bacmid DNA was extracted and transfected with FuGENE HD (Promega, cat. no. E231A) into the *Sf*9 insect cells for virus production. The supernatant (V_0_) containing the recombinant baculovirus was collected after 72 to 96 hours post-transfection. To expand the virus pool, 6.0 mL of the V_0_ virus stock was added into 25 mL of *Sf*9 (0.5 × 10^6^/mL) cells. The resulting cell culture supernatant (V_1_) was collected 24 h post-proliferation arrest. For large-scale expression of the protein, Hi5 cells were infected with virus (V_1_) and cells were harvested 72 h post-proliferation arrest.

For bacterial expression, plasmids were transformed into the *E. coli* BL21(DE3) strain and expression was initiated by addition of 0.7 mM Isopropyl β-D-1-thiogalactopyranoside (IPTG) when the culture density reached 0.6 (OD_600_). The proteins were then expressed overnight at 20°C following induction.

#### Purification of METTL3-METTL14 complex

Insect cells co-expressing hMETTL3 and hMETTL14 were resuspended in the lysis buffer (50 mM Tris-HCl pH 8.0, 300 mM NaCl, 40 mM Imidazole, 5% glycerol, 0.1% Triton X-100, 5 mM 2-mercaptoethanol, proteinase inhibitor (Roche, Complete EDTA-free) and Benzonase (Millipore), sonicated with MISONIX Sonicator S-4000 and the lysate was centrifuged at 20,000 rpm for 30 min at 4°C. The clarified supernatant was incubated at 4°C for 2h with the Ni^2+^ chelating Sepharose FF beads (GE Health; cat. no. 17057501). The beads were washed with buffer W300 (50 mM Tris-HCl pH 8.0, 500 mM NaCl, 50 mM Imidazole, 0.1% Triton X-100, 5 mM 2-mercaptoethanol) and W500 (50 mM Tris-HCl pH 8.0, 500 mM NaCl, 40 mM Imidazole, 0.2% Triton X-100, 5 mM 2-mercaptoethanol). Finally, His-tag proteins bound to the beads were eluted with the elution buffer (50 mM Tris-HCl pH 8.0, 300 mM NaCl, 300 mM Imidazole, 0.1% Triton X-100, 5 mM 2-mercaptoethanol). The tag (His-Strep-Sumo) was cleaved overnight with TEV in the dialysis buffer (50 mM Tris-HCl pH 8.0, 250 mM NaCl, 5 mM 2-mercaptoethanol). After cleavage, second Ni-column purification was performed and supernatant containing the cleaved protein was collected. Proteins were further purified over the ion exchange column (HiTrap™ Q Sepharose HP, 1ml, GE healthcare, cat. no. 17-1153-01). Fractions containing the recombinant proteins were further purified by gel filtration chromatography using Superdex S200 10/300GL equilibrated with gel-filtration buffer containing: 50 mM Tris-HCl pH 8.0, 200 mM NaCl, 5 mM 2-mercaptoethanol (GE Healthcare, cat. no. 17-5175-01). The fractions eluting at 11 mL of elution volume were checked by SDS-PAGE analysis (Figure S1C) and pure hMETTL3-hMETTL14 protein complexes were concentrated and flash frozen in liquid nitrogen after addition of 10% glycerol.

#### Purification of METTL16

The insect cells or bacterial cells were collected by centrifugation and lysed by sonication [25 mM Tris-HCl, pH 8.0, 400 mM NaCl, 5% Glycerol, 0.5% Tween-20, 5 mM 2-mercaptoethanol, 20 mM Imidazole and protease inhibitor (Roche complete EDTA-free)]. After incubation for two hours with Ni-NTA beads, the fusion protein was eluted with Imidazole (250 mM), and the His-SUMO tag was cut by the TEV protease (10 μg of protease per 1 mg of fusion protein; EMBL Protein expression and purification facility). The cleaved tag was removed by a second purification on Ni-NTA beads. The protein was further purified by gel filtration chromatography (Superdex S75 or Superdex 200, GE Healthcare) in the buffer (25 mM HEPES, pH 7.2, 150 mM NaCl, 2 mM DTT). The elution volumes of both full-length METTL16 and METTL16-core and METTL16-ΔN during gel-filtration chromatography are consistent with the proteins being a monomer (Figure S1B). The pure fractions were verified by SDS-PAGE (Figure S1E), and used for crystallization and biochemical assays. One of the METTL16-core mutants (Loop-3R-E) showed aberrant migration in the denaturing gel, but its identity was confirmed by mass spectrometry and shows normal elution profiles during gel-filtration chromatography (Figure S2F).

#### Limited proteolysis of hMETTL16-FL

For limited proteolysis, we used a 1:1000 ratio of protease:protein (if the protease is freshly prepared, use 1:500 ratio). Take 100 μL of METTL16-FL (concentration 1 μg/μl) protein solution and mix with 2 μL the protease Trypsin (concentration is 50 ng/μl). This makes a total of 102 μL reaction mix. Incubate at 25°C and remove aliquots of 25 μL at time-points 0, 5, 30 and 60 minutes. Aliquots are immediately mixed with gel loading dye, boiled at 95°C, and stored at −20°C. Reactions are then resolved via SDS-PAGE (Figure S1E). Peptide boundaries of proteolysis fragments were identified by mass spectrometry at the Proteomics Core Facility, EMBL, Heidelberg.

#### Crystallization and data collection

Optimal crystallization conditions for full-length human METTL16 (1-562 aa) and the human METTL16-core (1-291 aa) proteins were sought by robot screening at the High Throughput Crystallization Facility at EMBL Grenoble, France. Only the METTL16-core gave crystals in this screen. Once conditions were identified, crystals were manually produced: 2 μL protein solution at 13 mg/ml was manually mixed with 2 μL reservoir solution using the hanging drop method at room temperature. The reservoir conditions used were either 0.2 M di-sodium tartrate, 20% (w/v) PEG 3350 or 0.1 M Bis-Tris propane, pH 6.5, 0.2 M potassium-sodium tartrate, 20% (w/v) PEG 3350. We additionally crystallized the human METTL16-ΔN (40-291 aa) version using conditions previously described in PDB ID: 2H00 [Structural Genomics Consortium (SGC)]. The crystals were then flash-frozen at 100K after transferring them to identical crystallization conditions containing 20% glycerol. Diffraction data were collected on ID23-2 (Flot et al., 2010) and ID30B (McCarthy et al., 2018) at the European Synchrotron Radiation Facility (Grenoble, France), and integrated using the XDS suite (Kabsch, 2010). The diffraction data from hMETTL16-core (1-291 aa) crystals were highly anisotropic, with diffraction limits of ∼2.8 Å and 2.4 Å along the best direction for crystal form 1 and 2 respectively, but only ∼3.6 Å in the weakly diffracting directions. Therefore, data were processed using STARANISO (Tickle et al., 2017), as implemented in autoPROC (Vonrhein et al., 2011), which applies non-elliptical anisotropic limits based on a locally averaged mean *I*/σ(*I*) cut-off, performs a Bayesian estimation of structure amplitudes, and applies an anisotropic correction to the data. Detailed crystallographic statistics are provided in Table 1.

**Table 1:** Data Collection and Refinement Statistics

Protein PDB Code	ΔN MTase 6GFK	MTase (form 1, SAH) 6GFN	MTase (form 2, apo) 6GT5
Wavelength (Å)	0.9763	0.8731	0.8731
Resolution range (Å)	46–2.3 (2.38–2.3)	82–2.86 (3.2–2.86)	80–2.5 (2.8–2.5)
Space group	P3_1_21	I4_1_22	P4_1_2_1_2
Unit cell (Å)	133.8, 133.8, 78.7 90, 90, 120	93.4, 93.4, 180.7 90, 90, 90	89.6, 89.6, 179.1 90, 90, 90
Unique reflections	36,074 (3,538)	5,724 (286)	12,391 (619)

**Completeness (%)**			

Spherical	99.4 (99.6)	60.9 (10.5)	45.9 (6.8)
Ellipsoidal	N/A	93.1 (78.7)	93.3 (79.9)
Mean < I/σI >	10.3 (1.4)	8.0 (1.8)	4.5 (1.6)
R_pim_ (%)	3.7 (55.0)	7.3 (50.3)	,12.4 (50.2)
CC^∗^	0.994 (0.996)	0.996 (0.59)	0.967 (0.647)
R_work_ (%)	19.4 (21.2)	18.1 (22.1)	18.2 (24.0)
R_free_ (%)	22.8 (23.5)	21.6 (30.5)	23.4 (30.5)

**Number of non-H Atoms**			

Macromolecules	5,307	2,024	4,011
Water	100	11	25
SAH/ion	103	26	–
R_msd_ (bonds, Å)	0.009	0.01	0.009
R_msd_ (angles, ^o^)	1.06	1.11	1.10

**Ramachandran Plot (%)**			

Favored	97.3	94.4	93.2
Allowed	2.7	4.4	6.0

Statistics for the highest resolution shell are shown in parentheses.

#### Structure determination and refinement

The hMETTL16-core (1-291 aa) structure was solved by molecular replacement using the METTL16-ΔN, N-terminal deletion structure (PDB ID: 2h00) as a search model with Phaser (McCoy et al., 2007). Several rounds of manual building with Coot (Emsley et al., 2010), and structure refinement with BUSTER (Bricogne et al., 2016) were carried out for all structures. MOLPROBITY (Chen et al., 2010) was used for model validation and all the crystallographic information is summarized in Table 1. The atomic coordinates and structure factors have been deposited in the Protein Data Bank with the accession codes: 6GFN (METTL16-core, crystal form 1), 6GT5 (METTL16-core, crystal form 2) and 6GFK (METTL16-ΔN). For modeling a bound RNA into the METTL16-core structure we used a tRNA from PDB ID: 2ZZM (Goto-Ito et al., 2009). Structural figures were prepared with PyMOL (Schrödinger, LLC). The electrostatic potential was calculated using PDB2PQR (Dolinsky et al., 2004) and displayed in PyMOL using the APBS plugin. For modeling of adenosine into the METTL16-core structure we used a 20-mer DNA from the complex structure of the MTase EcoP15I (PDB: 4ZCF, chain B)(Gupta et al., 2015).

While this study was in preparation, Ruszkowska et al. reported the crystal structure (PDB ID: 6B92) of METTL16 core MTase domain (Ruszkowska et al., 2018). A comparison with our structure (PBD ID: 6GFN) reveals a very high degree of overlap (rmsd = ∼0.38 Å for superimposition of 187 C_α_ atoms) (Figure S1F). Nevertheless, there are some differences. First, the N terminus in our structure is longer by four amino acids. Second, there are differences in labile loops between α4-β3 (96-99 disordered in ours); α7-β6 (poor density in ours); and β6-α8 (the long catalytic loop). The catalytic NPPF residues (in both our METTL16-core structures, Table 1) are more similar to their apo form (PDB ID: 6B91) than their SAH-bound form (PDB ID: 6B92) (Ruszkowska et al., 2018). They also have eight additional residues on α8, but these are not helical as in our METTL16-ΔN structure (PBDB ID: 6GFK) (Figure 1D).

#### *In vitro* transcription of RNA substrates for methylation assay

Templates for *in vitro* transcription (of full-length human *MAT2A* mRNA hairpin 1 and human U6 snRNA RNA) (Pendleton et al., 2017) were amplified in a PCR reaction to prepare a single-stranded DNA template with T7 promoter sequence. The T7 promoter sequence 5ʹ- TAATACGACTCACTATAGGG −3ʹ was introduced at the 5ʹ end of forward primer followed by a specific sequence. The reverse primer had a 20 nt overlap with the forward primer allowing for efficient base pairing. The primers used for template preparation are given in Table S1. The following components were mixed to prepare the PCR reaction: 20 μl 5X Phusion HF buffer, 67 μl ddH2O, 2 μl 10 mM dNTPs, 5 μl of 10 μM Forward primer, 5 μl of 10 μM Reverse primer, and 1 μl Phusion DNA polymerase. The PCR reaction conditions were set as follows: 98°C for 30 s, 35 cycles of [98°C for 10 s, 60°C for 30 s and 72°C for 15 s], 72°C for 10 min, and finally at 4°C to hold the reaction. The PCR product (∼110bp) was agarose gel-purified using mini-elute gel extraction kit (QIAGEN, cat. no. 28604). The purified DNA was used to produce RNA by *in vitro* transcription reaction via the T7 promoter. *In vitro* transcription was carried out with the MEGAshortscript T7 Transcription Kit (Life technologies; cat no. AM1354) for 4 hours at 37°C. Reactions were treated with DNase I to remove template DNA, phenol-chloroform extracted and precipitated with ethanol. *MAT2A* hairpin RNA was 82 nt long, while the U6 snRNA was 83 nt long. Quality of the generated RNA was verified by 1.2% agarose gel electrophoresis.

#### *In vitro* RNA methylation assay with METTL16

Some methylation assays were carried out with *in vitro* transcribed RNAs (*MAT2A* mRNA hairpin 1 or U6 snRNA), while the majority were with chemically synthesized RNA oligos (Microsynth, Switzerland) (Table S1). Recombinant human METTL16 proteins (FL, core, ΔN and mutant versions) or a heterodimer of human METTL3/METTL14 were used.

Prior to the experiment, the RNAs were refolded by heating 100 μM RNA solution in 10 mM NaCl in a thermoblock to 70°C for 5 min. and slowly cooling down to room temperature, while keeping the tubes in a heat block. All methylation reactions were performed in a 50 mM Tris-HCl, pH 7.5, 100 mM KCl, 5 mM MgCl_2_, 2 mM DTT buffer with 10 μM of refolded single-stranded RNA, 5 μg of recombinant protein, 1 μl of RiboLock RNase Inhibitor (ThermoFisher, cat. no. EO0381) and 0.1 μCi of ^14^C-SAM (Perkin Elmer, NEC363010UC) in a total volume of 50 μl. Unless otherwise indicated, all reactions were performed overnight at 37°C. For reactions with RNA oligos designed based on m^6^A-IP-RNaseq experiment (Figure 3J), these were performed overnight at 22°C. RNA was subsequently isolated using phenol/chloroform extraction protocol. RNA pellets were resuspended in 2x RNA loading buffer (90% formamide, 0.02% SDS, 1 mM EDTA, 0.02% bromophenol blue, 0.02% xylene cyanol), heated for 5 min. at 70°C, cooled down to the room temperature and resolved in a 15% Urea-PAGE gel.

The 15% Urea-PAGE gel was prepared by mixing 12.6 g of urea, 3 mL of 10x TBE (1 M Tris base, 1 M boric acid, 0.02 M EDTA), 11.25 mL of 40% acrylamide (19:1) and 6.75 mL of H_2_O. To catalyze gel polymerization, 240 μl of APS and 24 μl of TEMED were added. Gel was left for 40 min. at room temperature to polymerize. Wells were flushed with 1XTBE to remove urea deposits and gel was pre-run in 1X TBE at 20 W for 25 min to warm the gel. After the pre-run, ssRNA marker labeled with ^32^P-γ-ATP and composed of four single-stranded RNA oligos (RP_RNA_19: 40 nt, RP_RNA_1: 30 nt, RP_RNA_3: 28 nt, RP_RNA_18: 16 nt; Table S1) was loaded into the gel, together with RNA samples from the *in vitro* methylation assay. Gel was run at 12 W for 1 h 30 min. Then, dried in a gel dryer (Bio-Rad, model 583) with a gradual heating and cooling program, 80°C for 3 h. Dried gel was exposed to a phosphor screen BAS (GE Healthcare) for 24 h. The phosphor screen was scanned in a Typhoon FLA 9500 laser scanner (GE Healthcare) at 700V and 100 μm pixel size using control software (1.1 version) for Typhoon FLA 9500. Scans were analyzed using ImageQuant TL 8.1 software (GE Healthcare).

The quality of RNAs used for methylation assays were verified by Methylene Blue staining. In some experiments, after the methylation reaction products were resolved by urea-PAGE, the gel was stained with Methylene Blue, imaged to verify integrity of RNAs present in the reaction (Figure S3A) and then dried for exposure to the phosphor storage screen to detect radioactivity signals (Figure 3A).

#### UV crosslinking assay

*Preparation of labeled RNA*: RNA6 (100 pmol) was 5ʹ-end labeled with [γ-^32^P]ATP and T4 Polynucleotide Kinase (NEB, M0201) for 1 h at 37°C. Labeled RNA was resolved on 15% Urea-PAGE gel and exposed with phosphor screen BAS (GE Healthcare) for 5 minutes. RNA band corresponding to the size of 29 nt was cut from the gel. The RNA was eluted from gel by overnight incubation in 300 mM NaCl at room temperature and with shaking (750 rpm). RNA was extracted by phenol-chloroform and resuspended in 20 μl of H_2_O. See Table S1 for RNA sequence.

METTL16 proteins (final concentration 0.4 μM and 2 μM) were mixed with 1 μl of labeled RNA6 in RNA binding buffer (10 mM Tris-HCl, pH 8.0, 50 mM NaCl, 1 mM DTT, 1 mM SAH) in a final volume of 20 μl and incubated for 2 h on ice. After incubation, reaction mix was deposited inside the cap of the Eppendorf tube, and placed on ice such that the cap touches the ice. Tubes were placed around 4 cm from UV lamp (254 nm) and irradiated for 5 min. (UV Stratalinker 2400, Stratagene). After UV irradiation, samples were boiled for 5 min in SDS loading buffer and resolved by 12% SDS-polyacrylamide gel electrophoresis.

Gel was dried in a gel dryer (Bio-Rad, model 583) with a gradual heating and cooling program, 80°C for 3 h. Dried gel was transferred to the cassette and exposed with a phosphor screen BAS (GE Healthcare) for 24 h. After exposure, phosphor screen was scanned in a Typhoon FLA 9500 laser scanner (GE Healthcare) at 700V and 50 μm pixel size. Control software for Typhoon FLA 9500 was at 1.1 version. Scans were analyzed using ImageQuant TL 8.1 software (GE Healthcare).

#### Cell culture and transfections

Human embryonic kidney cell line 293 (HEK293) transformed with the SV40 large T antigen (HEK293T) were grown in Dulbecco’s modified Eagle Medium (DMEM; Invitrogen, cat. No. 21969-035) supplemented with 10% fetal bovine serum (ThermoFisher; cat. no. 10270106), 1% Penicilline/Streptomycin (ThermoFisher; cat. No. 15140122), 1% 200 mM Glutamine (ThermoFisher; cat. no. 15140122), later referred to as DMEM complete medium (DMEM CM), and maintained in an environment with 5% CO_2_ at 37°C. For transfection, cells growing in a 75 cm^2^ flask were washed with warm (37°C) 1X PBS and incubated with 1 mL of Trypsin-EDTA 0.05% (ThermoFisher; cat. no. 25300-054) for 1-2 min to promote removal of cells from the growth surface. Subsequently, 10 mL warm DMEM media was added and cells were resuspended by pipetting. Cells were counted using Bürker-Türk and appropriate cell numbers were seeded in cell culture vessels.

Approximately, 4 mL of HEK293T cells were seeded in the 10 cm dish (Falcon, cat. no. 353003) and cultured as described above. When 40 – 50% confluence was reached, cells were transfected with FLAG-HA-METTL16 plasmid: 10 μg of plasmids was diluted in 500 μL of 150 mM NaCl. Simultaneously, 26 μg of linear polyethylenimine, MW 25000 (PEI, Polysciences Inc., cat. no. 23966) was diluted in 500 μL of 150 mM NaCl. Solutions were mixed together, vortexed vigorously for 15 s and incubated for 15 min. at room temperature. Then mix was added to the HEK293T cells in the DMEM CM. After 24 h, medium was changed for the fresh DMEM CM. Cells were grown for 72h in total.

#### Isolation of human METTL16 complexes for mass spectrometry

Cells in 10 cm dishes were washed 3x with ice cold PBS and 1 mL of lysis buffer [20 mM Tris pH 7.4, 150 mM NaCl, 0.5% Triton X-100, 0.1% sodium deoxycholate, 1 mM EDTA, 0.5 mM DTT, protease inhibitor (Complete Protease Inhibitor Cocktail Tablet, Roche, cat. no. 5056489001)] was added to the cells. Cells were removed from their growth surface using a cell scraper (Costar; cat. no. 3010) and transferred to 1.5 mL eppendorf tubes. Cell lysate was passed 5-times through a 26 G needle (B. Braun Medical Inc., #466-5457) and kept on ice for 15 min. The total cell lysate was spun at 12,000 x g for 10 min at 4°C. After centrifugation, supernatant was transferred to a fresh tube and spun again (12,000 x g, 10 min., 4°C). The cleared lysate was transferred to a fresh tube. While 50 μl of lysate was transferred to a fresh tube and flash-frozen in liquid nitrogen to use as an input, 950 μl was incubated for 4 h at 4°C with 20 μL of Anti-HA Affinity Matrix (Roche, cat. no. 11815016001). After, beads were collected by gentle centrifugation (500 x g for 1 min at 4°C) and eluate was discarded. Beads were washed 5 times with wash buffer (50 mM Tris pH 7.4, 150 mM NaCl, 0.1% Triton X-100, 1 mM EDTA) Then, beads were transferred to fresh 1.5 mL eppendorf tubes and 40 μL of 2x Laemmli buffer (4% SDS, 20% glycerol, 120 mM Tris-HCl pH 6.8, 10% β-mercaptoethanol, 0.02% bromophenol blue) was added. Beads were boiled at 95°C for 5 minutes and stored at −20°C. Proteins were identified by mass spectrometry at the Functional Genomics Center Zurich (ETH Zurich) (Figure S5F). Database searches were performed using the Mascot (SwissProt, human) search program. Applied settings: 1% protein false detection rate (FDR), min. 2 peptides per protein, 0.1% peptide FDR.

#### Isolation of METTL16 complexes from mouse testes and spleen

An aliquot of 80 μl of Dynabeads Protein A slurry (ThermoFisher, 10001D) was transferred to a fresh Eppendorf tube and washed three times with 1 mL of 20 mM sodium phosphate with 0.02% Tween20. Then, 20 μg of METTL16 antibody (abcam, ab186012) or 20 μg of mouse IgG control antibody (Santa Cruz, cat.no. sc-2025) in 500 μl of 20 mM sodium phosphate with 0.02% Tween20 was added to the beads and incubated overnight at 4°C with rotation.

Next day, two adult (P60) mouse testes and one spleen were cut into pieces using scalpel blade and placed into separate 1.5 mL eppendorf tubes. 500 μL of ice cold lysis buffer [20 mM Tris pH 7.4, 150 mM NaCl, 0.5% Triton X-100, 0.5% sodium deoxycholate, 1 mM DTT, 1 mM EDTA and protease inhibitor (Roche) was added to the tubes. Organs were dounced 15-times using a plastic pestle and left on ice for 10 min. Then, tubes were spun at 12000 x g at 4°C for 10 minutes. Supernatant was transferred to a fresh tube and centrifugation was repeated to clarify the lysate further. Supernatants were transferred to a fresh tube and diluted 2x with dilution buffer dilution buffer [20 mM Tris pH 7.4, 150 mM NaCl, 1 mM EDTA, protease inhibitor] to decrease sodium deoxycholate and Triton X-100 concentration to 0.25%. An aliquot of 50 μl of lysate was transferred to a fresh tube and flash-frozen in liquid nitrogen to use as an input, while rest was transferred to antibody-bound Dynabeads prepared above, and incubated at 4°C for 4 h with rotation.

After 4 h, the supernatant was removed and beads were washed with wash buffer (20 mM Tris pH 7.4, 150 mM NaCl, 0.2% Triton X-100, 1 mM EDTA, 0.5 mM DTT). Washing was repeated four more times, after which beads were transferred to the fresh 1.5 mL eppendorf tubes and 40 μL of 2x Laemmli buffer (4% SDS, 20% glycerol, 120 mM Tris-HCl pH 6.8, 10% β-mercaptoethanol, 0.02% bromophenol blue) was added. Beads were boiled at 95°C for 5 minutes and stored at −20°C. Proteins in the samples were identified at the Functional Genomics Center Zurich (ETH Zurich) with the shotgun mass spectrometry analysis (Figure S5G). Database searches were performed using the Mascot (SwissProt, human) search program. Applied settings: 1% protein false detection rate (FDR), min. 1 peptides per protein, 0.1% peptide FDR.

#### Mass spectrometry

Mass spectrometry to confirm purified recombinant proteins were carried out at the Proteomics Core Facility, EMBL, Heidelberg. Identification of components within an immunoprecipitated complex was carried out at the Functional Genomics Center Zurich (ETH Zurich) using the shotgun mass spectrometry analysis. Database searches were performed using the Mascot (SwissProt, all species) search program. Applied settings if not stated differently are 1% protein false detection rate (FDR), min. 2 peptides per protein, 0.1% peptide FDR.

#### Preparation of RNA libraries

##### *In vitro* methylation with METTL16 and m^6^A-IP-RNaseq

Libraries of randomized 30 nt RNA sequences were chemically synthesized (Microsynth, CH). The sequences had a constant central 9-mer sequence flanked by randomized (represented by N) sequences (MM-RNA-14: N_11_-UAC**A**GAGAA-N_10_). The 9-mer sequence originates from the hairpin 1 of the human *MAT2A* mRNA and carries the m^6^A methylation site for METTL16 (Pendleton et al., 2017). RNA solutions (100 μM) with 50 mM NaCl were denatured at 80°C for 1 min, and refolded by allowing to cool to room-temperature. *In vitro* methylation reactions containing 15 μL (100 μM) of the above RNA library were carried out in 100 mL reactions (50 mM Tris-HCl, pH 7.5, 100 mM KCl, 5 mM MgCl_2_, 20U of Riboblock RNase inhibitor, 0.64 mM SAM) with 20 μg of hMETTL16. Reactions were carried out in duplicates and incubated at 37°C, overnight. Reactions were then removed and frozen at −20°C prior to further processing.

A small portion (10%) was left aside to be used as input sample, while the remainder was subjected to immunoprecipitation. The m^6^A immunoprecipitation was performed as described (Ke et al., 2015). Briefly, 100 μL of Dynabeads Protein A (Life Technologies; 10002D) were washed once in PXL buffer (1 × PBS, 0.1% SDS, 0.5% sodium deoxycholate, 0.5% NP-40) followed by pre-treatment with BSA (final concentration 1μg/ μL) in 200 μL PXL buffer for 45 minutes at room-temperature (RT). BSA pre-treated beads was then conjugated with m^6^A rabbit polyclonal antibody (5 μg; Synaptic Systems, catalog no. 202003) in 200 μL PXL buffer supplemented with 4 μL of RNasin RNase inhibitor (Promega; N2611) for one hour at RT on a rotating wheel. Dynabeads were further washed twice with PXL buffer and finally beads were resuspended in 400 μL of PXL buffer and 5 μL of RNasin. The *in vitro* methylation reaction prepared above was added to the beads and incubated at 4°C for 2 hours on a rotating wheel. After two hours incubation, the beads were washed twice by ice-cold Nelson low-salt buffer (15 mM Tris at pH 7.5, 5 mM EDTA), once by ice-cold Nelson high-salt buffer (15 mM Tris at pH 7.5, 5 mM EDTA, 2.5 mM EGTA, 1% Triton X-100, 1% sodium deoxycholate, 0.1% SDS, 1 M NaCl), once by ice-cold Nelson stringent wash buffer (15 mM Tris at pH 7.5, 5 mM EDTA, 2.5 mM EGTA, 1% Triton X-100, 1% sodium deoxycholate, 0.1% SDS, 120 mM NaCl, 25 mM KCl), and last by ice-cold NT-2 buffer (50 mM Tris at pH 7.4, 150 mM NaCl, 1 mM MgCl2, 0.05% NP-40). Antibody-bound RNAs were eluted by incubating the beads with 0.5 mg/mL *N*^*6*^-methyl adenosine (Sigma-Aldrich; M2780) in NT2 buffer for one hour at 4°C. The eluted RNAs were precipitated with ethanol and glycogen and dissolved in RNase-free water.

The input and IP RNAs were first 3ʹ end dephosphorylated with T4 PNK (NEB; M0201S, 10 U/μL) in the absence of ATP at 37°C for 45 minutes (40 μL reaction: 35.5 μL RNA, 4 μL 10X T4 PNK buffer, 0.5 μL of T4 PNK) followed by phosphorylation of 5ʹ end (50 μL reaction: 40 μL dephosphoryated RNA, 6.5 μL water, 1 μL RNasin, 0.5 μL 100 mM ATP, 1 μL 10X T4 PNK buffer 1 μL T4 PNK) at 37°C for 45 minutes. RNAs were phenol chloroform-extracted, ethanol precipitated and resupended in 6 μL of RNase-free water. The input RNA fragments and the immunopurified RNAs after the phosphorylation step were directly used for library preparation (barcoded at 3′ end) using NEBNext® Multiplex Small RNA Library Prep Set for Illumina® (NEB; catalog No. E7560S) following manufacturer’s instructions. The synthesized cDNA libraries were resolved on 3% high-resolution MethaPhor agarose (Lonza; cat. No. 50180) gels in 1X TAE buffer at 70 V. Fragments in the size-range of ∼150-250 bp were gel-extracted with the use of MinElute Gel Extraction Kit (QIAGEN; cat No. 28604). Multiple libraries with different barcodes (at 3′ end) were mixed in equimolar ratios and sequenced with the HiSeq Illumina Platform (EMBL GeneCore facility, Heidelberg). The maximum sequencing length was 50 nt. The list of sequencing libraries generated are provided in Table S2.

##### Mouse single-embryo library preparation

Polyadenylated transcripts in single embryos (E2.5 morula or E3.5 blastocysts) were amplified using the Smart-seq2 protocol (Picelli et al., 2014). The protocol generates libraries that lack strand specificity. Multiple libraries with different barcodes (at the 3′ end) were mixed in equimolar ratios and paired-end sequencing reads were obtained with the HiSeq Illumina Platform (EMBL GeneCore facility, Heidelberg). The maximum sequencing length was 80 nt. The list of sequencing libraries generated are provided in Table S2.

### Quantification and Statistical Analysis

#### *In vitro* methylation with METTL16 and m^6^A-IP-RNaseq

Reads were sorted into individual libraries based on the barcodes and the 3′ adaptor sequences were removed using cutadapt 1.9.1 (http://journal.embnet.org/index.php/embnetjournal/article/view/200). Only reads of final length of 30 nucleotides with correctly sequenced TACAGAGAA consensus motif at position 12-20 and without any Ns were kept for further analysis using R 3.4.3 (R Core Team, 2017) and Bioconductor (Huber et al., 2015). To search for possible preference of human METTL16 for specific structured RNA features, we analyzed the predicted secondary structures of the sequenced oligos and compare their representation in between the m^6^A-IP and input libraries. For each sequence we obtained the minimum free energy (MFE) secondary structure using RNAfold (Lorenz et al., 2011). We used DESeq2 1.18.1 bioconductor package (Love et al., 2014) to obtain the lists of structures significantly enriched or depleted in IP (immunoprecipitation) libraries when compared to input libraries (adjusted p value < 0.1). Top enriched structures were plotted in dot bracket notation (DBN) (Figures 3E and 3F). To search for preferred features in IP-enriched structures, for every structure and each position based on DBN, we checked whether it is part of the stem, is in a loop, is in a bulge or if it is in between two stems. Then we compared the proportion of the structures having nucleotide at specific position in a stem, loop, etc, in between IP-enriched structures, IP-depleted structures and structures with no difference in their abundance between m^6^A-IP and input (Figure 3G). In IP-enriched structures we observed a clear preference of A at position 15 (in the motif UAC**A**GAGAA), which is methylated by METTL16, to be in a single nucleotide bulge or to lie in a region surrounded by stem structures (Figure 3G). To see the differences in IP-enriched and depleted structures, we also plotted the log2 difference of the frequencies for IP-enriched (or depleted) structures when related to the structures not differentially represented between m^6^A IP and input (Figure 3H). While the IP-enriched structures had higher proportion of 15A in a single nucleotide bulge or lying between two stems, the IP-depleted structures showed the opposite trend, with less proportion of structures with 15A in a bulge or in between two stems. For calculations of these log2 ratios of the frequencies, the frequencies lower than 0.5% were considered to be 0.5. Proportion of individual structures in which 15A can be found is summarized in a barplot (Figure 3I). We also directly compared the frequencies of oligos with individual positions in a stem, loop etc. in between m^6^A-IP libraries and input libraries (Figure S3C) and their log2 ratios, separately for both replicas (Figure S3D). In m^6^A-IP libraries we observed increased proportion of oligos where 15A is in single nucleotide bulge or in between two stems.

To check whether there is specific sequence preference outside the TAC**A**GAGAA consensus motif, we compared the nucleotide frequencies at individual oligo positions between IP and input samples and plotted their log2 ratios (Figure S3E). In the IP-oligos we observed general higher frequencies of G and C. We used MEME - Motif discovery tool 4.11.2 (Bailey and Elkan, 1994) to search for any sequence motif in the IP enriched left 11-mers and right 10-mers surrounding the TAC**A**GAGAA (Figure S3F).

#### Transcriptome analysis of Mettl16 mutant mouse embryos

Paired-end reads were sorted into individual libraries based on the barcodes and aligned to NCBI RefSeq transcripts (build mm10) using Salmon v0.7.2 (Patro et al., 2017). The genotype of the mouse embryos giving rise to the individual samples was assessed based on the presence of the reads derived from WT *Mettl16* allele (containing CACCAGGATTCGGACAAAACTA or TAGTTTTGTCCGAATCCTGGTG sequence, since libraries are non-strand-specific) and from *Mettl16* KO allele (containing TCACCAATGTAAATAGATGAGG or CCTCATCTATTTACATTGGTGA sequence). For E2.5 there were 5 *Mettl16*^+/+^ (WT), 14 *Mettl16*^+/−^ (HET) and 12 *Mettl16*^−/−^ (KO) samples. For E3.5 we got 18 *Mettl16*^+/+^ (WT), 9 *Mettl16*^+/−^ (HET) and 12 *Mettl16*^−/−^ (KO) samples

The transcript estimates were imported into DESeq2 1.18.1 (Love et al., 2014) and summarized to gene levels using tximport 1.2.0 (Soneson et al., 2015). The DESeq2 was used to obtain lists of differentially expressed genes with statistical significance (adjusted p value ≤ 0.1). The MA plots were plotted using graphics::smoothScatter function and the individual genes with significantly different expression were highlighted (Figures 4D and 5C).

For the E2.5 dataset, twenty genes were found to be differentially expressed between some of the genotypes (Figure 4E; Table S3) and their expression was visualized by heatmap using the made4::heatplot function (Figure 4E). Only four of the genes had significantly different expression in *Mettl16*^−/−^ versus *Mettl16*^+/−^ and also in *Mettl16*^−/−^ versus *Mettl16*^+/+^ comparison (Figure S5B). Boxplots of normalized counts were plotted for those genes, with individual samples plotted as dots using graphics:stripchart function (Figures 4F and S5C). To visualize the coverage of individual genomic loci, the sequenced reads were aligned to reference mm10 genome using STAR (Dobin et al., 2013) and the normalized coverage was calculated using GenomicRanges::coverage function. Mean coverages were plotted for individual genotypes using Gviz 1.22.3 (Hahne and Ivanek, 2016) together with the transcript annotation obtained either from NCBI RefSeq track from UCSC or from GENCODE M17 (Figures 4G and S5E). Gviz was also used to plot the coverage of individual exons or introns. To compare the amount of reads coming from individual introns of *Mat2a*, featureCounts (Liao et al., 2014) was used to obtain the counts for individual genomic exons and introns which were then normalized by DESeq2. Boxplots of the counts were plotted for individual introns of *Mat2a* normalized to library sizes or to overall *Mat2a* counts (Figure S5D). The intron coordinates used were shortened by 10 nucleotides from both sides so that the intron counts were not affected by exonic reads partially protruding into the introns. The JunctionSeq 1.8.0 (Hartley and Mullikin, 2016) was used to search for differential usage of splice junctions among the genotypes. Only few splice junctions were significantly (adjusted p value ≤ 0.01) differentially used between *Mettl16*^−/−^ versus *Mettl16*^+/−^ and also in *Mettl16*^−/−^ versus *Mettl16*^+/+^ comparison (Table S3). Whereas the reads spanning the splice junction of last *Mat2a* (NM_145569 = ENSMUST00000059472.9) intron were depleted in *Mettl16*^−/−^ when normalized to overall *Mat2a* transcript levels, alternative splice junction (common for ENSMUST00000206904.1 and ENSMUST00000206692.1) was elevated (Figure 4H) as shown by boxplots. This was accompanied by overall increase of last *Mat2a* intron counts in *Mettl16*^−/−^ which is however not significant.

For the E3.5 dataset, 5166 genes were found to be differentially expressed between some of the genotypes (Figure 5D; Table S3) and their expression was visualized by heatmap using the made4::heatplot function (Figure 5D). Most of the genes which were found to be dysreguled in *Mettl16*^−/−^ versus *Mettl16*^+/−^ were also differentially expressed between *Mettl16*^−/−^ and *Mettl16*^+/+^ (Figure 5E). Enriched gene ontology biological processes for upregulated and downregulated genes were identified by ENRICHR (Chen et al., 2013, Kuleshov et al., 2016) and are summarized in Table S3. Boxplots of normalized counts were plotted for *Mettl16*, *Mat2a* and top dysregulated genes with individual samples plotted as dots using graphics:stripchart function (Figures 5B and S6D). FeatureCounts was used to obtain summarized counts for introns, exons and repeats which did not show any differences between the genotypes neither in E2.5 nor in E3.5 (Figures S5A and 5G) and also to obtain the counts for individual genomic exons and introns. Boxplot was used to compare number of reads arising from last intron of *Mat2a* and splice junction reads crossing the last intron (Figure S6A) whose counts were obtained from JunctionSeq analysis. All the splice junctions which were significantly (adjusted p value ≤ 0.01) differentially used between *Mettl16*^−/−^ versus *Mettl16*^+/−^ and also in *Mettl16*^−/−^ versus *Mettl16*^+/+^ comparison are summarized in Table S3. Overall counts of uniquely mapping reads crossing the splice junctions were obtained from SJ.out.tab files generated by STAR and their proportion was compared between individual samples (Figure 5F). To find out whether genes specific to any developmental stage are misregulated in the mutant, we checked the expression of the key transcription and chromatin factors characteristic for different stages (Table S1 of Mohammed et al., 2017). Heatmap of log2 (normalized counts +1) expression was plotted using gplots::heatmap.2 from individual samples (Figure S6B). Boxplots were used to compare the average expression change between *Mettl16*^−/−^ versus *Mettl16*^+/−^ and *Mettl16*^−/−^ versus *Mettl16*^+/+^, with individual genes plotted as dots (Figure S6C).

### Data and Software Availability

Deep sequencing data generated in this study are deposited with Gene Expression Omnibus under the accession number GSE116329. Crystallographic data are deposited with Protein Data Bank under PDB accessions: 6GFN, 6GT5 and 6GFK. Other raw data associated with this study are deposited with Mendeley Data under the accession https://doi.org/10.17632/ny82j2ngt5.1. The *Mettl16* knockout mutant mouse generated in this study will be available from the Lead Contact.

## Supplemental Information

Document S1. Figures S1–S6 and Tables S1 and S2

Table S3. List of Genes and Splice Junctions with Differential Expression in *Mettl16* Mutant E2.5 and E3.5 Embryos; Related to Figures 4 and 5

Document S2. Article plus Supplemental Information
