# Supplementary material for: Methylation of Structured RNA by the m6A Writer METTL16 Is Essential for Mouse Embryonic Development
Source: Mol Cell. 2018 Sep 20;71(6):986–1000.e11. doi: 10.1016/j.molcel.2018.08.004 (PMC6162343; doi:10.1016/j.molcel.2018.08.004)
Supplement: Document S1. Figures S1–S6 and Tables S1 and S2 [file mmc1.pdf]

Molecular Cell, Volume *71*

Supplemental Information

Methylation of Structured RNA by the m^6A Writer METTL16 Is Essential for Mouse Embryonic Development

Mateusz Mendel, Kuan-Ming Chen, David Homolka, Pascal Gos, Radha Raman Pandey, Andrew A. McCarthy, and Ramesh S. Pillai

INVENTORY OF SUPPLEMENTAL INFORMATION

One PDF with

1) Supplemental Figures (S1-6)

2) Supplemental figure legends (S1-6)

3) Supplemental Tables (S1-2) and legends

Supplemental Figures

Figure S1. Full-length human METTL16 exists as monomers. Related to Figure 1.

Figure S2. Mutational analysis of the human METTL16-core methyltransferase domain to define the RNA-binding groove. Related to Figures 1 and 2.

Figure S3. In vitro methylation with human METTL16-FL and a randomized RNA library reveals structural and sequence requirements for m^6A RNA methylation. Related to Figure 3.

Figure S4. Embryonic lethality in *Mettl16* knockout mice around implantation stage. Related to Figure 4.

Figure S5. Lack of METTL16 has very specific and limited effect on the transcriptome of E2.5 embryos. Related to Figure 4.

Figure S6. Dramatically altered transcriptome of E3.5 *Mettl16* knockout embryos. Related to Figure 5.

Supplemental Tables

Table S1. DNA primers and RNA oligonucleotides used in this study. Related to STAR Methods and Figure 1,2 and 3.

Table S2. List of all deep-sequencing libraries created in this study. Related to STAR Methods and Figure 4 and 5.

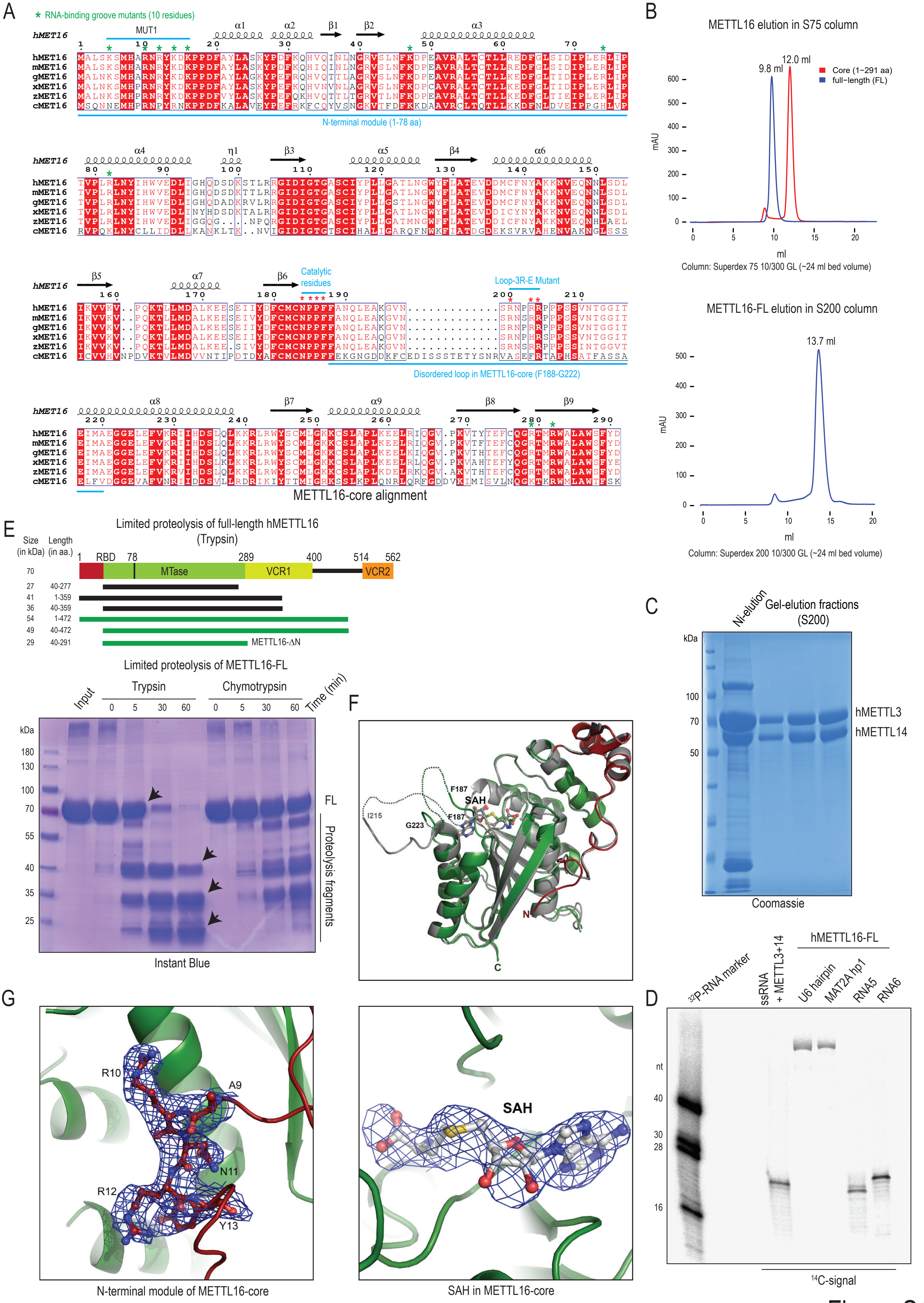

A
* RNA-binding groove mutants (10 residues)
MUT1
N-terminal module (1-78 aa)
Catalytic residues
Loop-3R-E Mutant
Disordered loop in METTL16-core (F188-G222)
METTL16-core alignment
hMET16
mMET16
gMET16
xMET16
zMET16
cMET16
B
METTL16 elution in S75 column
Core (1−291 aa)
full−length (FL)
9.8 ml
12.0 ml
mAU
ml
Column: Superdex 75 10/300 GL (~24 ml bed volume)
METTL16-FL elution in S200 column
13.7 ml
Column: Superdex 200 10/300 GL (~24 ml bed volume)
C
Ni-elution
Gel-elution fractions (S200)
kDa
hMETTL3
hMETTL14
Coomassie
D
32P-RNA marker
ssRNA + METTL3+14
hMETTL16-FL
U6 hairpin
MAT2A hp1
RNA5
RNA6
nt
14C-signal
E
Limited proteolysis of full-length hMETTL16 (Trypsin)
Size (in kDa)
Length (in aa.)
RBD
MTase
VCR1
VCR2
METTL16-ΔN
Limited proteolysis of METTL16-FL
Input
Trypsin
Chymotrypsin
Time (min)
FL
Proteolysis fragments
Instant Blue
F
F187
SAH
I215
G223
N
C
G
R10
A9
N11
R12
Y13
N-terminal module of METTL16-core
SAH in METTL16-core

Figure-S1

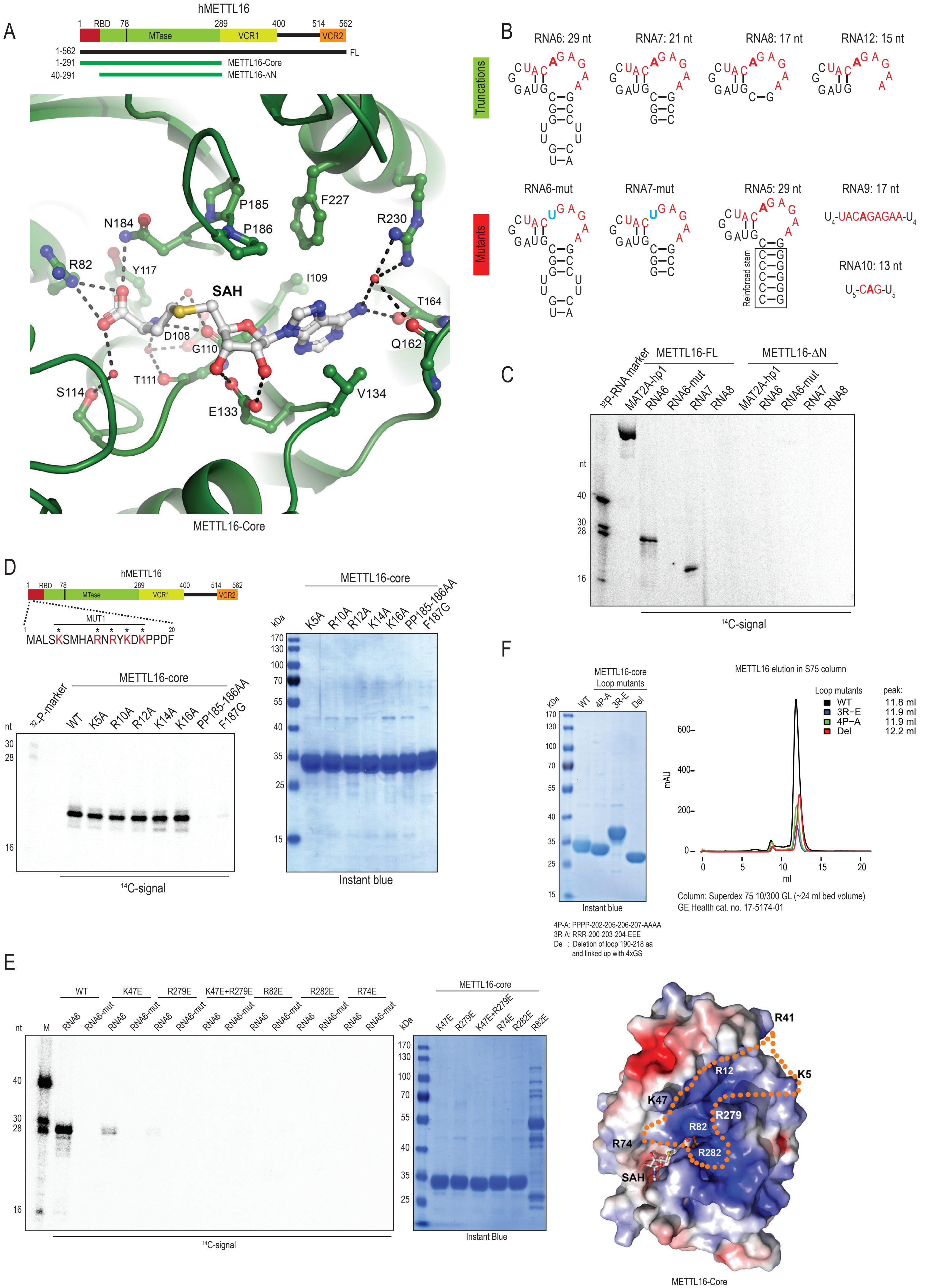
A
hMETTL16
1 RBD 78 289 400 514 562
MTase
VCR1
VCR2
1-562
FL
1-291
METTL16-Core
40-291
METTL16-ΔN
P185
P186
F227
N184
R230
R82
Y117
SAH
I109
T164
D108
G110
Q162
T111
S114
V134
E133
METTL16-Core
B
Truncations
Mutants
RNA6: 29 nt
RNA7: 21 nt
RNA8: 17 nt
RNA12: 15 nt
RNA6-mut
RNA7-mut
RNA5: 29 nt
Reinforced stem
RNA9: 17 nt
U4-UACAGAGAA-U4
RNA10: 13 nt
U5-CAG-U5
C
METTL16-FL
METTL16-ΔN
32P-RNA marker
MAT2A-hp1
RNA6
RNA6-mut
RNA7
RNA8
nt
40
30
28
16
14C-signal
D
hMETTL16
MUT1
MALSKSMHARNRYKDKPPDF
METTL16-core
32P-marker
WT
K5A
R10A
R12A
K14A
K16A
PP185-186AA
F187G
14C-signal
kDa
170
130
100
70
55
40
35
25
15
Instant blue
E
WT
K47E
R279E
K47E+R279E
R82E
R282E
R74E
M
RNA6
RNA6-mut
14C-signal
Instant Blue
R41
R12
K5
K47
R279
R82
R74
R282
SAH
METTL16-Core
F
METTL16-core
Loop mutants
WT
4P-A
3R-E
Del
Instant blue
4P-A: PPPP-202-205-206-207-AAAA
3R-A: RRR-200-203-204-EEE
Del : Deletion of loop 190-218 aa and linked up with 4xGS
METTL16 elution in S75 column
Loop mutants
peak:
WT 11.8 ml
3R-E 11.9 ml
4P-A 11.9 ml
Del 12.2 ml
mAU
ml
Column: Superdex 75 10/300 GL (~24 ml bed volume)
GE Health cat. no. 17-5174-01

Figure-S2

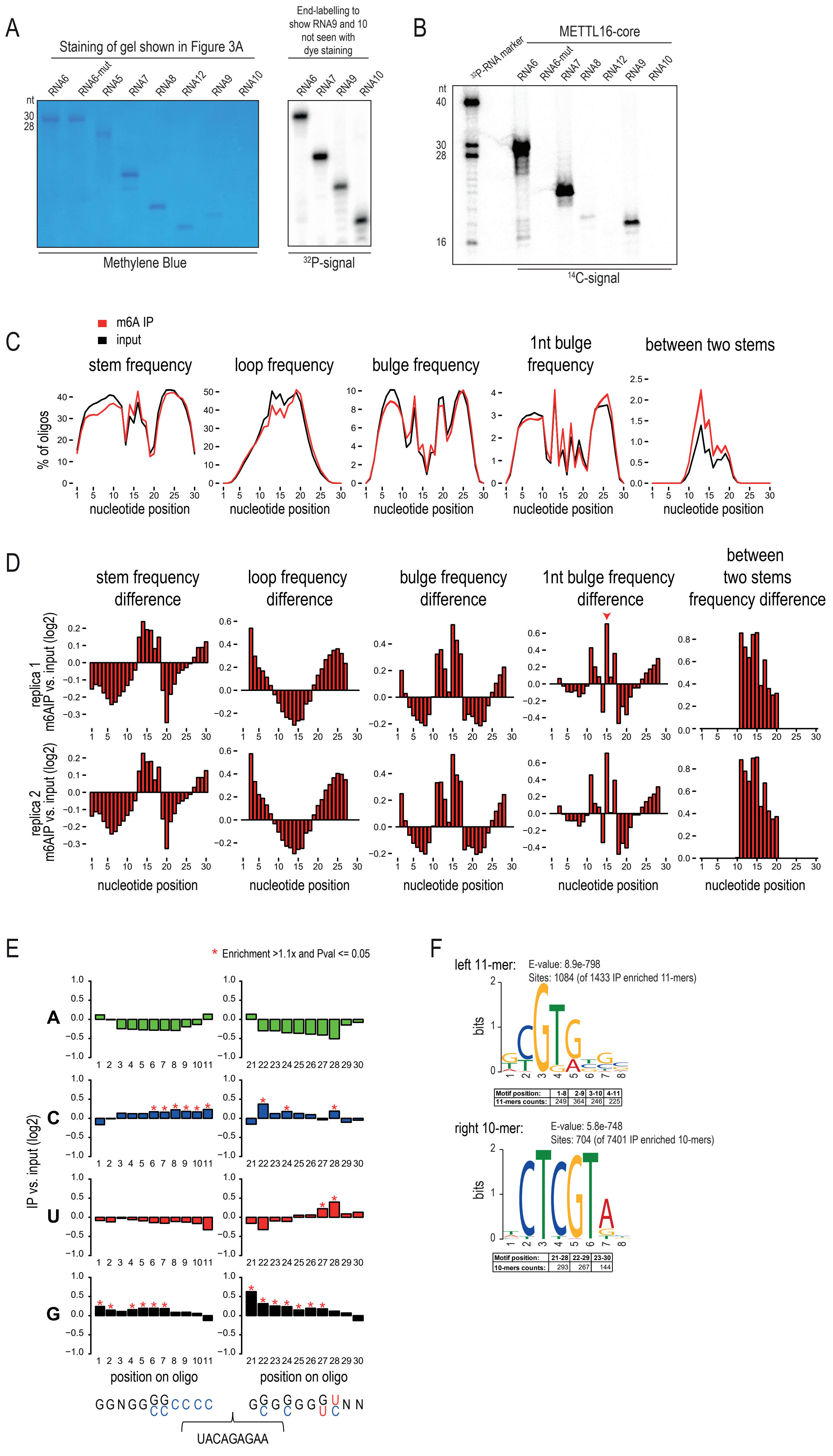
A
Staining of gel shown in Figure 3A
End-labelling to show RNA9 and 10 not seen with dye staining
RNA6 RNA6-mut RNA5 RNA7 RNA8 RNA12 RNA9 RNA10
RNA6 RNA7 RNA9 RNA10
nt
30
28
Methylene Blue
32P-signal
B
METTL16-core
32P-RNA marker
RNA6 RNA6-mut RNA7 RNA8 RNA12 RNA9 RNA10
nt
40
30
28
16
14C-signal
C
m6A IP
input
stem frequency
loop frequency
bulge frequency
1nt bulge frequency
between two stems
% of oligos
nucleotide position
D
stem frequency difference
loop frequency difference
bulge frequency difference
1nt bulge frequency difference
between two stems frequency difference
replica 1 m6AIP vs. input (log2)
replica 2 m6AIP vs. input (log2)
nucleotide position
E
* Enrichment >1.1x and Pval <= 0.05
A
C
U
G
IP vs. input (log2)
position on oligo
GGNGGGGCCCCC
GGGGGGGUNN
UACAGAGAA
F
left 11-mer:
E-value: 8.9e-798
Sites: 1084 (of 1433 IP enriched 11-mers)
bits
Motif position: 1-8 2-9 3-10 4-11
11-mers counts: 249 364 246 225
right 10-mer:
E-value: 5.8e-748
Sites: 704 (of 7401 IP enriched 10-mers)
bits
Motif position: 21-28 22-29 23-30
10-mers counts: 293 267 144

Figure-S3

A

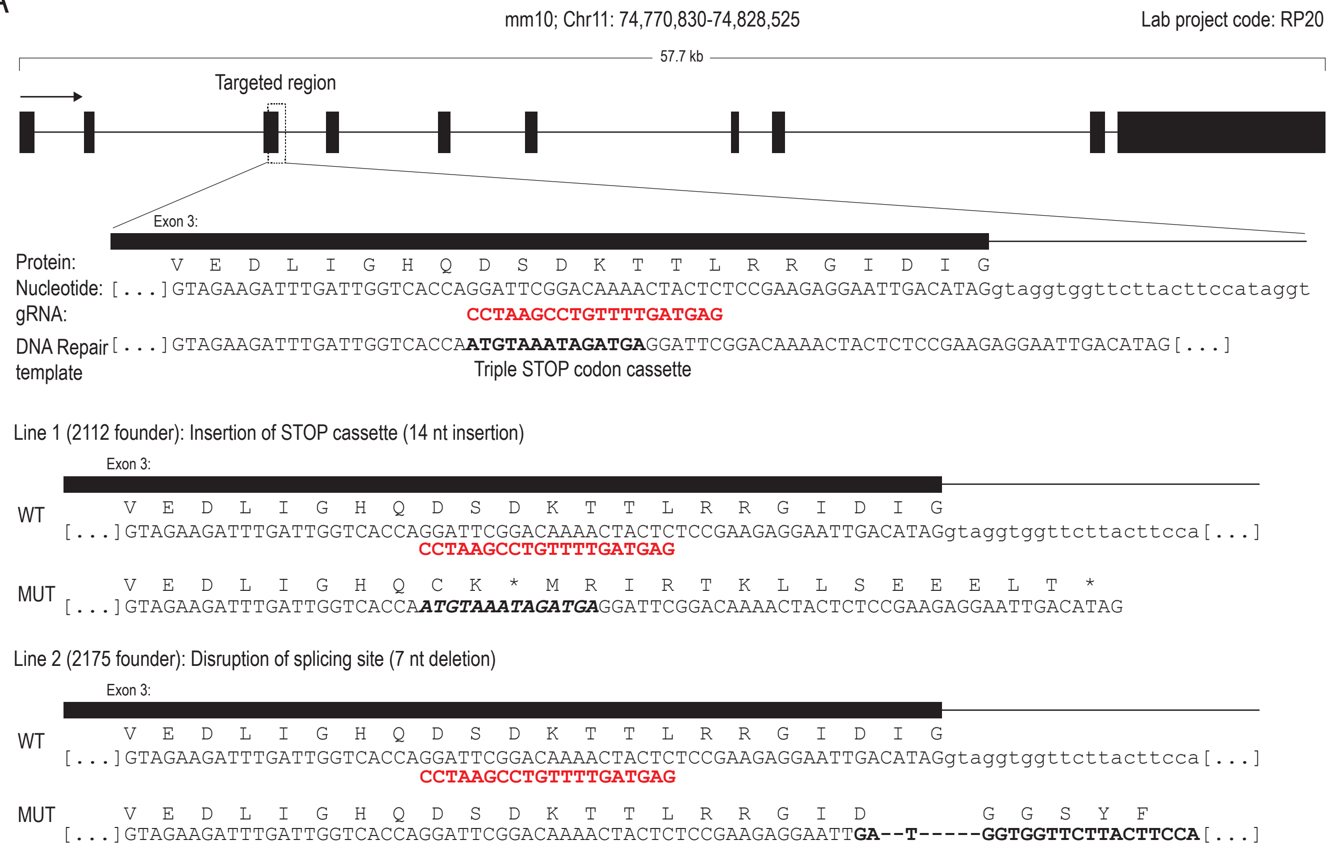

B

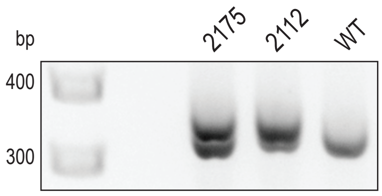

C

Genotype of animals in litters at weaning age (P21) from *Mettl16+/-* x *Mettl16+/-* crosses

	Total	KO	HET	WT	Male	Female
**Number**	**156**	0	114	42	73	83
**Percentage**	**-**	0.0%	73.1%	26.9%	46.8%	53.2%

D

Genotype of E6.5 embryos from *Mettl16+/-* x *Mettl16+/-* crosses

	Placentas	Embryos	WT	HET	KO	Unknown
**Number**	68	52	16	32	1	3
**Percentage**	-	76.5%	30.8%	61.5%	1.9%	5.8%

E

Genotype of E8.5 embryos from *Mettl16+/-* x *Mettl16+/-* crosses

	Placentas	Embryos	WT	HET	KO
**Number**	24	14	1	13	0
**Percentage**	-	58%	7%	93%	

F

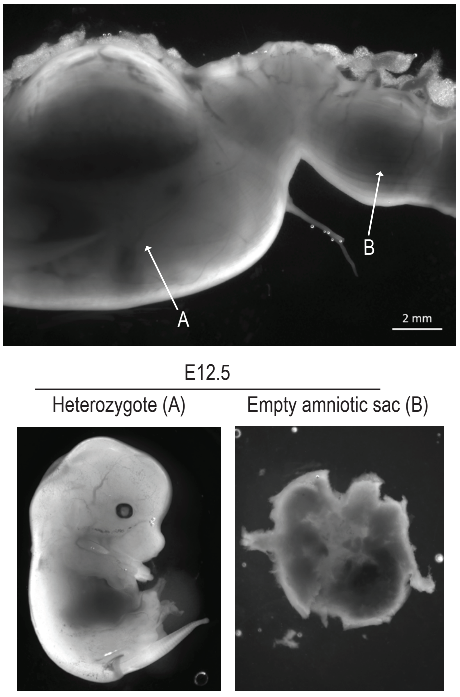

Figure-S4

A

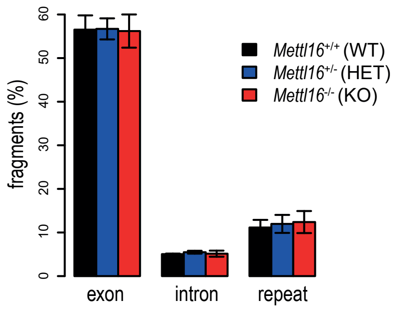

B

	log2 fold change			adjusted p-value		
gene	KO vs WT	KO vs HET	HET vs WT	KO vs WT	KO vs HET	HET vs WT
Ccdc92b	-1.63	-1.39	-0.24	4.79E-02	6.43E-03	1.00E+00
Mettl16	-1.83	-1.32	-0.52	4.31E-11	3.13E-10	1.00E+00
Mat2a	-2.41	-2.00	-0.41	2.69E-31	5.03E-40	1.00E+00
Gm15698	-2.58	-2.16	-0.41	6.40E-07	1.65E-09	1.00E+00

C

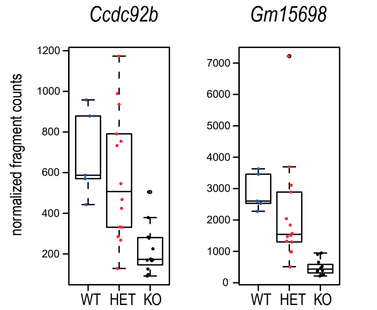

D

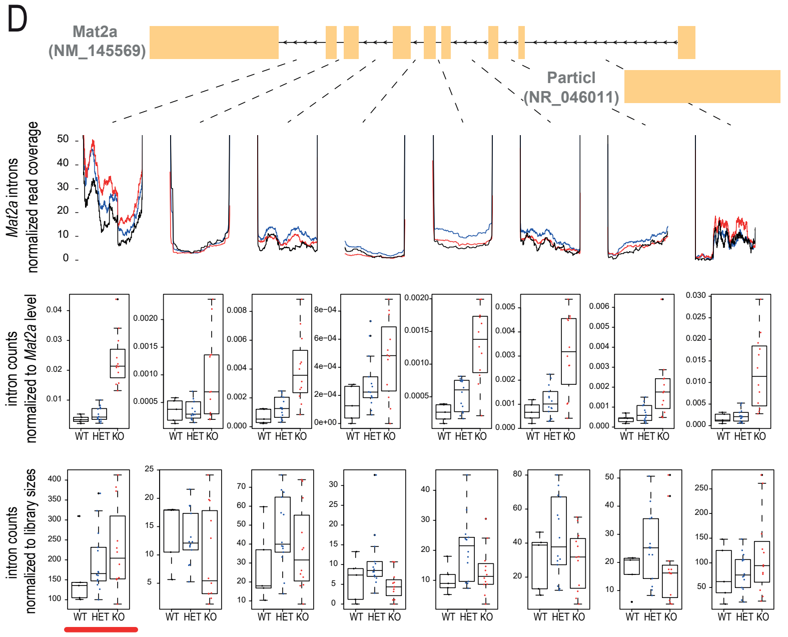

E

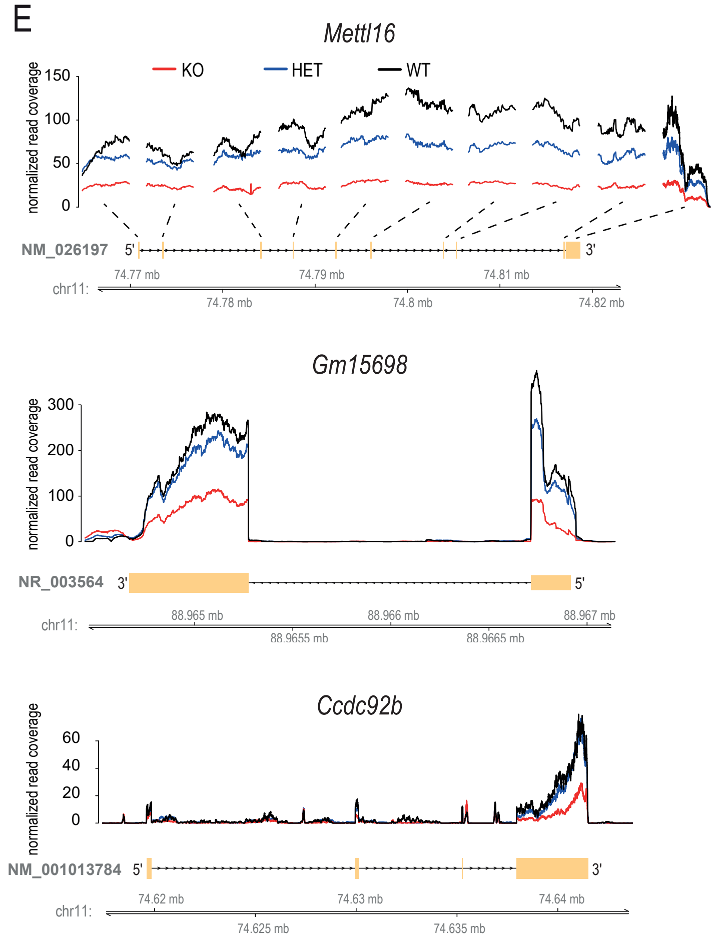

F

METTL16 Complex from HEK293 cells

No.	Name	ID	MW [kDa]	METTL16 Rep. 1	METTL16 Rep. 2	METTL16 Rep. 3	GFP Rep. 1	GFP Rep. 2
				Exclusive Unique Peptide Count				
1	Kanadaptin	SLC4A1AP	89	26	24	18	4	0
2	U6 small nuclear RNA (adenine-(43)-N(6))-methyltransferase	METTL16	64	74	84	77	13	5
3	Lupus La protein	SSB	47	9	7	4	0	2
4	DNA repair protein RAD50	RAD50	154	5	3	6	0	2
5	Splicing factor 3B subunit 2	SF3B2	100	19	17	15	5	2
6	Deoxynucleoside triphosphate triphosphohydrolase SAMHD1	SAMHD1	72	4	9	7	0	3
7	Splicing factor 3B subunit 1	SF3B1	146	34	21	33	9	6
8	Kinesin-like protein KIF18B	KIF18B	94	4	6	6	1	2
9	Structural maintenance of chromosomes protein 4	SMC4	147	4	3	4	0	0
10	Zinc finger CCCH domain-containing protein 8	ZC3H8	34	2	5	4	0	2
11	WD repeat-containing protein 82	WDR82	35	4	2	5	2	0
12	Splicing factor 3B subunit 3	SF3B3	136	23	8	22	6	6
13	Furin	FURIN	87	3	5	5	0	3
14	Probable 28S rRNA (cytosine(4447)-C(5))-methyltransferase	NOP2	89	2	7	3	2	0
15	Galectin-1	LGALS1	15	2	3	6	2	0

G

METTL16 Complex from mouse tissues

No.	Name	ID	MW [kDa]	Testis	Testis	Spleen	IgG CTRL	IgG CTRL
				Exclusive Unique Peptide Count				
1	Splicing factor 3B subunit 1	SF3B1	146	28	33	51	0	0
2	Splicing factor 3B subunit 3	SF3B3	136	21	25	33	0	0
3	Protein PRRC2A	PRRC2A	229	24	32	13	0	0
4	Kinesin-like protein KIF13B	KIF13B	203	24	23	7	0	0
5	Probable ATP-dependent RNA helicase DDX5	DDX5	69	22	20	33	0	0
6	Cleavage and polyadenylation specificity factor subunit 1	CPSF1	161	28	19	22	0	0
7	pre-mRNA 3' end processing protein WDR33	WDR33	145	24	19	22	0	0
8	Splicing factor 3B subunit 2	SF3B2	100	20	17	28	0	0
9	Splicing factor 3A subunit 1	SF3A1	89	10	19	30	0	0
10	Protein scribble homolog	SCRIB	174	19	21	5	0	0
11	Gem-associated protein 5	GEMIN5	167	21	13	18	0	0
12	U6 small nuclear RNA (adenine-(43)-N(6))-methyltransferas	METTL16	62	17	17	14	0	0
13	Serine/arginine repetitive matrix protein 2	SRRM2	295	12	28	16	0	0
14	Cleavage and polyadenylation specificity factor subunit 2	CPSF2	88	21	14	16	0	0
15	Heterogeneous nuclear ribonucleoprotein M	HNRNPM	78	19	14	15	0	0

Figure-S5

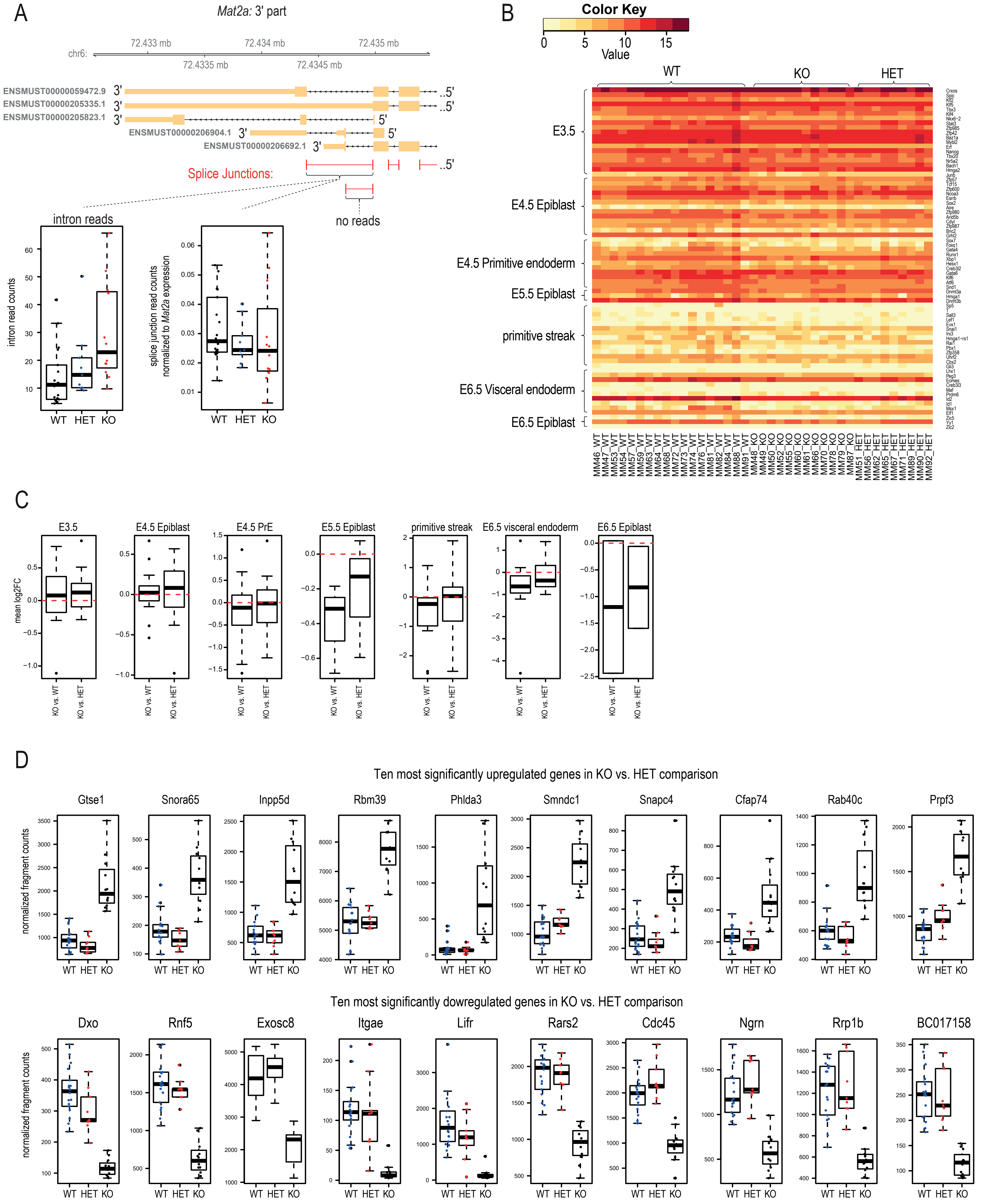
A
Mat2a: 3' part
chr6:
72.433 mb
72.434 mb
72.435 mb
72.4335 mb
72.4345 mb
ENSMUST00000059472.9
ENSMUST00000205335.1
ENSMUST00000205823.1
ENSMUST00000206904.1
ENSMUST00000206692.1
Splice Junctions:
no reads
intron reads
intron read counts
splice junction read counts normalized to Mat2a expression
WT HET KO
B
Color Key
Value
WT
KO
HET
E3.5
E4.5 Epiblast
E4.5 Primitive endoderm
E5.5 Epiblast
primitive streak
E6.5 Visceral endoderm
E6.5 Epiblast
C
E3.5
E4.5 Epiblast
E4.5 PrE
E5.5 Epiblast
primitive streak
E6.5 visceral endoderm
E6.5 Epiblast
mean log2FC
KO vs. WT
KO vs. HET
D
Ten most significantly upregulated genes in KO vs. HET comparison
Gtse1
Snora65
Inpp5d
Rbm39
Phlda3
Smndc1
Snapc4
Cfap74
Rab40c
Prpf3
normalized fragment counts
Ten most significantly dowregulated genes in KO vs. HET comparison
Dxo
Rnf5
Exosc8
Itgae
Lifr
Rars2
Cdc45
Ngrn
Rrp1b
BC017158

Figure-S6

Table S1. DNA and RNA oligonucleotides used in this study.
Related to STAR Methods and Figure 1,2 and 3.

**DNA oligos**		
**Name**	**Sequence**	**Comments**
CRISPR F primer	GAAATTAATACGACTCACTATAGGGAGTAGTTTTGTCCGAATCCGTTTTAGAGCTAGAAATAGC	RRoligo770
CRISPR sgR primer	CAAAATCTCGATCTTTATCGTTCAATTTTATTCCGATCAGGCAATAGTTGAACTTTTTCACCGTGGCTCAGCCACGAAAA	
MMoligo109	ATTCCCACAGTCCCCTTGAG	Genotyping PCR
MMoligo110	CAATGCCCAACCAACACAGA	Genotyping PCR
XXXXX	AGTTGAGAATGCAAAACCTATGGAAGTAAGAACCACCTACCTATGTCAATTCCTCTTCGGAGAGTAGTTTTGTCCGAATCC***TCATCTATTTACAT***TGGTGACCAATCAAATCTTCTACCCAGTGAATATAGTTAAGTCTCAAGGGGACTGTGGGAATTAGTCTCTCCAAA	ssDNA repair template
MMoligo81	TAATACGACTCACTATAGGGTGTTAGCCTTTTTTCCCCAGACTTGTTGGCGTAGGCTACA	MAT2A hp1 template forward
MMoligo82	GGAAGGAGGGCCCTTTCCCTCAGAGCTTGAAGGCTTCTCTGTAGCCTACGCCAACAAGTC	MAT2A hp1 template reverse
MMoligo83	TAATACGACTCACTATAGGGAAAATTGGAACGATACAGAGAAGATTAGCATGGCCCCTGC	U6 snRNA template forward
MMoligo84	AAAATATGGAACGCTTCACGAATTTGCGTGTCATCCTTGCGCAGGGGCCATGCTAATCTT	U6 snRNA template reverse
**RNA oligos**		
**Lab Name**	**Sequence**	**Name in this study**
MMRNA6	UGUUGGCGUAGGCUACAGAGAAGCCUUCA	RNA6 ; 29nt
MMRNA11	UGUUGGCGUAGGCUACUGAGAAGCCUUCA	RNA6-mut ; 29nt
MMRNA7	GGCGUAGGCUACAGAGAAGCC	RNA7 ; 21nt
MMRNA8	CGUAGGCUACAGAGAAG	RNA8 ; 17nt
MMRNA5	CCCCCCCGUAGGCUACAGAGAAGGGGGGG	RNA5 ; 29nt
MMRNA12	GUAGGCUACAGAGAA	RNA12 ; 15nt
MMRNA9	UUUUUACAGAGAAUUUU	RNA9 ; 17nt
MMRNA10	UUUUUCAGUUUUU	RNA10 ;13nt
MMRNA16	GGGGUACAGAGAAGGGG	RNA16 ; 17nt
MMRNA14	N11-UACAGAGAA-N10	Library;30nt; N= randomized
MMRNA21	CAGUAGCGACGUACAGAGAAACAUCUUCUC	RNA21 ; 30nt
MMRNA22	CAGUAGCGACGUACUGAGAAACAUCUUCUC	RNA22 ; 30nt
MMRNA23	CCGGCUUAGCCUACAGAGAACCUUUCUCGU	RNA23 ; 30nt
MMRNA24	CCGGCUUAGCCUACUGAGAACCUUUCUCGU	RNA24 ; 30nt
MMRNA28	GGGCGUAGGCNACANNNNNGCCC	RNA28 ; 23 nt ; N= randomized
MMRNA29	GGGCNNAGGCNNNANNNNNGCCC	RNA29 ; 23 nt ; N= randomized
MMRNA30	GGCGUAGGCUACAGACUUGCC	RNA30 ; 21 nt
MET1 RNA	UACACUCGAUCUGGACUAAAGCUGCUC	METTL3/14 substrate
MET2 RNA	UACACUCGAUCUGGAUUAAAGCUGCUC	METTL3/14 substrate(mut)
RP_RNA_1	UGACAUGAACACAGGUGCUCAGAUAGCUUU	RNA marker- 30nt
RP_RNA_3	UGACAUGAACACAGGUGCUCAGAUAGCU	RNA marker-28nt
RP_RNA_18	AGCACCGUAAAGACGC	RNA marker-16nt
RP_RNA_19	GCGUCUUUACGGUGCUUAAAACAAAACAAAACAAAACAAA	RNA marker-40nt

Supplemental Table S2. List of all deep-sequencing libraries created in this study. Related to STAR Methods

experiment	sample	*Mettl16* genotype	reads
RNASeq of E2.5 embryo	MM14	HET	34679836
RNASeq of E2.5 embryo	MM15	HET	28734242
RNASeq of E2.5 embryo	MM16	KO	34160424
RNASeq of E2.5 embryo	MM17	KO	36085991
RNASeq of E2.5 embryo	MM18	HET	33601058
RNASeq of E2.5 embryo	MM19	HET	32744958
RNASeq of E2.5 embryo	MM20	KO	36987647
RNASeq of E2.5 embryo	MM21	HET	33169466
RNASeq of E2.5 embryo	MM22	HET	32156604
RNASeq of E2.5 embryo	MM23	KO	32000383
RNASeq of E2.5 embryo	MM24	KO	30556292
RNASeq of E2.5 embryo	MM25	WT	23023349
RNASeq of E2.5 embryo	MM26	HET	33038807
RNASeq of E2.5 embryo	MM27	WT	31126991
RNASeq of E2.5 embryo	MM28	HET	32684662
RNASeq of E2.5 embryo	MM29	KO	29378767
RNASeq of E2.5 embryo	MM31	HET	35120157
RNASeq of E2.5 embryo	MM32	WT	32219943
RNASeq of E2.5 embryo	MM33	HET	32009135
RNASeq of E2.5 embryo	MM34	KO	32613795
RNASeq of E2.5 embryo	MM35	KO	29911795
RNASeq of E2.5 embryo	MM36	KO	35371390
RNASeq of E2.5 embryo	MM37	HET	34461243
RNASeq of E2.5 embryo	MM38	KO	34035525
RNASeq of E2.5 embryo	MM39	HET	33553354
RNASeq of E2.5 embryo	MM40	KO	31275672
RNASeq of E2.5 embryo	MM41	KO	32321617
RNASeq of E2.5 embryo	MM42	WT	34131480
RNASeq of E2.5 embryo	MM43	HET	33347148
RNASeq of E2.5 embryo	MM44	HET	35295970
RNASeq of E2.5 embryo	MM45	WT	25935853

experiment	sample	*Mettl16* genotype	reads
RNASeq of E3.5 embryo	MM46	WT	27418677
RNASeq of E3.5 embryo	MM47	WT	27497901
RNASeq of E3.5 embryo	MM48	KO	30804681
RNASeq of E3.5 embryo	MM49	KO	31884501
RNASeq of E3.5 embryo	MM50	KO	34965952
RNASeq of E3.5 embryo	MM51	HET	33312807
RNASeq of E3.5 embryo	MM52	KO	28476667
RNASeq of E3.5 embryo	MM53	WT	31001004
RNASeq of E3.5 embryo	MM54	WT	26071380
RNASeq of E3.5 embryo	MM55	KO	31392715
RNASeq of E3.5 embryo	MM56	HET	26518367
RNASeq of E3.5 embryo	MM57	WT	36455849
RNASeq of E3.5 embryo	MM59	WT	35482923
RNASeq of E3.5 embryo	MM60	KO	31141823
RNASeq of E3.5 embryo	MM61	KO	30971406
RNASeq of E3.5 embryo	MM62	HET	31630583
RNASeq of E3.5 embryo	MM63	WT	42925667
RNASeq of E3.5 embryo	MM64	WT	46540640
RNASeq of E3.5 embryo	MM65	HET	47186347
RNASeq of E3.5 embryo	MM66	KO	46106111
RNASeq of E3.5 embryo	MM67	HET	42360160
RNASeq of E3.5 embryo	MM68	WT	44228364
RNASeq of E3.5 embryo	MM70	KO	34314476
RNASeq of E3.5 embryo	MM71	HET	38949449
RNASeq of E3.5 embryo	MM72	WT	38861981
RNASeq of E3.5 embryo	MM73	WT	51611166
RNASeq of E3.5 embryo	MM74	WT	50932521
RNASeq of E3.5 embryo	MM76	WT	45598521
RNASeq of E3.5 embryo	MM78	KO	27609275
RNASeq of E3.5 embryo	MM79	KO	38122632
RNASeq of E3.5 embryo	MM81	WT	43648518
RNASeq of E3.5 embryo	MM82	WT	37246976
RNASeq of E3.5 embryo	MM83	NA	39136258
RNASeq of E3.5 embryo	MM84	WT	36941697
RNASeq of E3.5 embryo	MM87	KO	24036687
RNASeq of E3.5 embryo	MM88	WT	103815156
RNASeq of E3.5 embryo	MM89	HET	34466973
RNASeq of E3.5 embryo	MM90	HET	62009799
RNASeq of E3.5 embryo	MM91	WT	39750111
RNASeq of E3.5 embryo	MM92	HET	39921773

experiment	sample	description	reads	filtered reads
METTL16 in vitro IP and RNASeq	RR582	Input sample 11	42755991	21312366
METTL16 in vitro IP and RNASeq	RR583	Input sample 12	37993827	18869613
METTL16 in vitro IP and RNASeq	RR586	m6A IP sample 11	42519953	23104554
METTL16 in vitro IP and RNASeq	RR587	m6A IP sample 12	36825565	1203361

SUPPLEMENTAL FIGURE LEGENDS

Figure S1. Full-length human METTL16 exists as monomers. Related to Figure 1.

(A) Protein sequence alignment of the methyltransferase domain of METTL16 proteins. MET16, METTL16; h, human (mammal); m, mouse (mammal); g, *Gallus* (bird); x, *Xenopus* (amphibian); z, zebrafish (fish); c, *C.elegans* (nematode). The secondary structure features present in the human METTL16-core (PDB ID: 6GFN) are indicated above: α helices, β-strands and η-3_{10} helix. Residues (marked with green asterisks) in the putative RNA-binding groove where mutated and shown to affect methylation activity and/or RNA-binding (tested for MUT1 only, in Figure 2F). Deletion of the disordered loop or other mutated residues (red asterisks) also abolish activity. (B) Gel-filtration chromatography profile for indicated proteins on two different Superdex columns (S75 and S200). The elution profile of the proteins are consistent with them being a monomer. The full-length (FL) METTL16 was produced in insect cells, while the METTL16-core was expressed in *E.coli*. (C) Purification of full-length human METTL3/METTL14 complex. See STAR Methods. (D) In vitro methylation assay with ^{14}C-SAM, METTL16-FL or the METTL3+METTL14 complex, and indicated RNAs. The single-stranded RNA (ssRNA) used for METTL3+14 reaction is MET1RNA, while full-length human U6 snRNA and full-length human *MAT2A* hairpin (hp) 1 were in vitro transcribed (STAR methods), and others were purchased (Table S1). Single-stranded RNA markers (size in nucleotides, nt) are ^{32}P-end-labelled. (E) Limited proteolysis of METTL16-FL with indicated proteases. An Instant Blue-stained SDS-PAGE with aliquots of the time-course reaction, with incubation times in minutes (min) and protein markers (in kDa) is shown. Protein boundaries of the fragments were identified by mass spectrometry from bands (indicated by arrows) in the gel. (F) Comparison of our crystal structure of the human METTL16 core (Green/Magenta; PDB ID: 6GFN) with the one recently published by Ruszkowska et al. (Grey; PDB ID: 6B92). A comparison reveals a very high degree of overlap between two structures with only minor differences (see STAR Methods). (G) Electron density for key regions identified in the METTL16-core structure. Shown on left, a 2Fo-Fc map contoured at 1.2 σ and coloured in blue for the METTL16 N-terminal region, A9 to Y13 (PDB: 6GTS). Shown on right, a 2Fo-Fc omit map contoured at 2 σ and coloured in blue for the METTL16 core SAH-binding site (PDB: 6GFN).

Figure S2. Mutational analysis of the human METTL16-core methyltransferase domain to define the RNA-binding groove. Related to Figures 1 and 2.

(A) Cartoon indicating protein domains of human METTL16. Boundaries of the two constructs crystallized in this study are shown (in green). A zoom of the catalytic domain in human METTL16-core domain showing the bound S-adenosyl-homocysteine (SAH) (PDB ID: 6GFK). Residues coordinating the SAH and the catalytic residues N184, P185, P186 are highlighted. (B) Predicted structures of various synthetic RNAs used in in vitro methylation assays. They are all derived from the human *MAT2A* hairpin (hp) 1 and the consensus methylation motif of METTL16 is highlighted (red). The adenosine (A) that is methylated is indicated in bold and this is mutated to a uridine (U) in some of the RNAs. RNA5 has a reinforced stem with artificial G:C pairs. (C) In vitro methylation assay with ^{14}C-SAM, METTL16 proteins and indicated RNAs. *MAT2A* hp1 was in vitro transcribed, while the others were purchased (Table S1). Single-stranded RNA markers (size in nucleotides, nt) are ^{32}P-end-labelled. Note that the METTL16-ΔN protein is inactive. (D) Cartoon showing the N-terminal 20 amino acids of human METTL16, with the positively charged residues that were individually mutated being highlighted (red with asterisks). In vitro methylation assay with wildtype (WT) or mutant METTL16-core versions indicated. Individual mutations of N-terminal positively charged residues to neutral alanine do not affect activity. See also Figure 2D. Catalytic-dead

mutations PP185-186AA and F187G result in absence of any activity. Quality of recombinant proteins used is shown (on the right). (E) In vitro methylation assay with human METTL16-core MTase proteins carrying mutations within the putative RNA-binding groove. The groove is outlined on the surface charge representation of the METTL16-core, on the right (this is same as shown in Figure 2C). Quality of the proteins used is shown. Note that almost all mutations abolish methylation activity. The RNA used are indicated (Table S1). (F) SDS-PAGE gel of the METTL16-core with mutations in the disordered loop. One of the proteins (Loop-3R-E) displays a retarded migration. Gel-filtration profile of the protein shows that its elution profile is same as the one seen for the wildtype protein or other mutants. See also Figure 2G.

Figure S3. In vitro methylation with human METTL16-FL and a randomized RNA library reveals structural and sequence requirements for m^6A RNA methylation. Related to Figure 3.

(A) Methylene blue dye staining of the same gel as shown in Figure 3A to reveal RNAs present in the reactions after in vitro methylation assays. After staining with the dye, gels were dried and exposed for detection of radioactivity, and is presented in Figure 3A. This shows the presence of RNAs of the expected sizes in all the lanes. RNA9 and 10 (uridine-rich sequences) are poorly stained with the dye. On the right, a few of the RNAs used were ^{32}P-end-labelled to reveal the integrity of the RNAs used (including RNA9 and RNA10). (B) A repetition of similar experiment as shown in Figure 3A, showing that dramatic truncations to the stem region of the *MAT2A* hairpin RNA abolishes activity. Note that RNA5 was not included in this experiment. (C) A randomized RNA library carrying the nonamer consensus sequence was incubated with METTL16-FL and m^6A-containing RNAs were enriched by immunoprecipitation (IP) and sequenced. See Figure 3D. Frequency of RNA oligos forming stem, loop and other selected features at individual positions is compared between m^6A-IP and input oligos. Methylated oligos have higher frequency of 15A (which is the adenosine predicted to be methylated in the nonamer consensus motif) in a bulge and surrounded by stems. See also Figure 3G. (D) Log_2 differences of oligo frequencies between m^6A-IP and input oligos indicate the 15A bulge and its positioning in between stem structures is important for being recognized and methylated by METTL16. Two replicate experiments show the same pattern. (E) Comparison of the nucleotide frequencies at individual oligo positions surrounding the consensus motif reveals higher G and C occurrence in methylated oligos. (F) Sequence motifs that were identified in the m^6A-IP-enriched 11-mers or 10-mers surrounding the consensus motif.

Figure S4. Embryonic lethality in *Mettl16* knockout mice around implantation stage. Related to Figure 4.

(A) Strategy for insertion of a triple-stop codon cassette into exon 3 of mouse *Mettl16* genomic locus using a guide RNA (gRNA) that targets the Cas9 DNA endonuclease. Homologous recombination (HR) introduces the DNA repair template with the triple-stop codon cassette into exon 3, disrupting the coding sequence, creating a knockout (KO) allele. Two independent lines were obtained and both showed identical embryonic lethality phenotype. Line#1 was used in this study for sequence analysis of embryos. (B) Genotyping PCR with mouse tail DNA from indicated mutant lines and wildtype. See STAR methods for PCR conditions. (C) Genotype of animals recovered in litters at the weaning stage (P21, post-natal day 21) from a cross of *Mettl16*$^{+/-}$ heterozygous (HET) parents. No homozygous *Mettl16*$^{-/-}$ knockout (KO) animals were present, indicating early lethality. (D) Genotyping of embryos collected at E6.5 from *Mettl16*$^{+/-}$ females crossed with *Mettl16*$^{+/-}$ males. Note that only one KO embryo was recovered. (E) Genotyping of embryos collected at E8.5 from *Mettl16*$^{+/-}$ females crossed with *Mettl16*$^{+/-}$ males. These studies reveal that loss of mouse *Mettl16* results in embryonic lethality around implantation stage. See also Figure 4B. (F) Examination of E12.5 embryos by genotyping. No KO embryos were detected, indicating early lethality. Note that only one litter was examined at E12.5. Scale bar in millimetre (mm) is shown.

Figure S5. Lack of METTL16 has very specific and limited effect on the transcriptome of E2.5 embryos. Related to Figure 4.

(A) Lack of METTL16 does not affect the global transcription from exons, introns and repeats. Error bars refer to standard deviation. (B) Only the transcripts of four genes have significantly differential abundance between in *Mettl16*$^{-/-}$ (KO) and *Mettl16*$^{+/-}$ (HET) and also between *Mettl16*$^{-/-}$ (KO) and *Mettl16*$^{+/+}$ (WT). (C) Boxplot of two genes significantly downregulated in *Mettl16*$^{-/-}$ (KO). Both of the genes lie on chromosome 11, same as the targeted *Mettl16*. (D) Normalized read coverage of *Mat2a* introns is shown together with the overall read counts for each intron. The intron read counts were normalized either just to library sizes (bottom row) or to *Mat2a* levels (middle row). One of the boxplots (marked with a red line) is reproduced in Figure 4H. (E) Normalized read coverage of the loci with three of the genes (except *Mat2a*) found to be significantly differentially expressed in *Mettl16*$^{-/-}$(KO). Only exon coverage is shown for *Mettl16*. Note that both *Gm15698* and *Ccdc92b* lie on chromosome 11 as well as *Mettl16* and their differential expression might be just the consequence of different chromosome 11 DNA sequence resulting from the mixed genetic background of parental strain which was crossed to pure B6 mouse (see STAR Methods). (F) Mass spectrometry analysis of 3xFLAG-HA-hMETTL16 immunopurified from transfected HEK293T cells. 3xFLAG-HA-GFP was used as a negative control. Presented protein hits are the top 15 most enriched proteins in the hMETTL16 IP when compared to the negative control (3xFLAG-HA-GFP). HA-tag purification was performed (STAR METHODS). Exclusive Unique Peptide Count shown. Protein threshold: 1% FDR; min. peptides: 2; Peptide threshold: 0.1% FDR. (G) Mass spectrometry analysis of endogenous mMETTL16 immunopurified from adult mouse testes and spleen. Presented protein hits are the top 15 most enriched proteins in the mMETTL16 IP when compared to the negative control. Multiple splicing factor 3B and 3A subunits are among the top candidates. Purification was done using anti-METTL16 antibody (abcam, ab186012) without RNase-treatment, negative control was beads bound with normal mouse IgG (Santa Cruz, sc-2025). Exclusive Unique Peptide Count shown. Protein threshold: 1% FDR; min. peptides: 2; Peptide threshold: 0.1% FDR.

Figure S6. Dramatically altered transcriptome of E3.5 *Mettl16* knockout embryos. Related to Figure 5.

(A) Counts of the reads spanning the splice junction (SJ) of last *Mat2a* (ENSMUST00000059472.9) intron are plotted together with read counts originating in the intron. Boxplots are shown where counts of individual samples are plotted as dots. (B) The heatmap shows the expression of transcription factors and chromatin modifiers enriched for distinct embryonic lineages [as defined by (Mohammed et al., 2017)] in individual samples. Expression of these key factors is unaltered in the *Mettl16* KO embryos. (C) Boxplots display the average log2 fold-changes (FC) between *Mettl16* mutant (KO) and control (WT or HET) mice for the groups of lineage-specific genes. Dots depict the individual genes. None of the group of lineage-specific genes seems to be differentially expressed between *Mettl16*$^{-/-}$ and *Mettl16*$^{+/-}$ or *Mettl16*$^{-/-}$ and *Mettl16*$^{+/+}$. (D) Boxplots compare the expression of the top 10 downregulated or upregulated genes in the *Mettl16*$^{-/-}$ KO blastocysts. Transcript levels of individual samples are shown as dots.

SUPPLEMENTAL TABLE LEGENDS

Table S1. DNA primers and RNA oligonucleotides used in this study. Related to STAR Methods and Figures 1-5.

Table S2. List of all deep-sequencing libraries created in this study. Related to STAR Methods and Figures 3-5.

Data is available from GEO under accession no. GSE116329.
