# Supplementary material for: Methylation of Structured RNA by the m6A Writer METTL16 Is Essential for Mouse Embryonic Development
Source: Mol Cell. 2018 Sep 20;71(6):986–1000.e11. doi: 10.1016/j.molcel.2018.08.004 (PMC6162343; doi:10.1016/j.molcel.2018.08.004)
Supplement: Document S2. Article plus Supplemental Information [file mmc3.pdf]

Molecular Cell

Article

Methylation of Structured RNA by the m6A Writer METTL16 Is Essential for Mouse Embryonic Development

Graphical Abstract

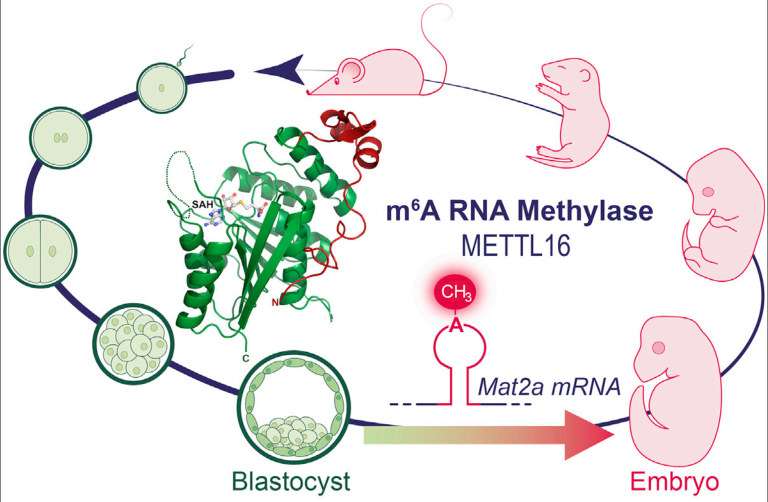

Authors

Mateusz Mendel, Kuan-Ming Chen, David Homolka, Pascal Gos, Radha Raman Pandey, Andrew A. McCarthy, Ramesh S. Pillai

Correspondence

ramesh.pillai@unige.ch

In Brief

Mendel et al. reveal the structural basis for structured RNA recognition by the mammalian m6A writer METTL16 and demonstrate its essential role in mouse early embryonic development via regulation of the SAM synthetase *Mat2a* mRNA.

Highlights

- Structure of the METTL16 m6A writer domain with a unique N-terminal module
- N-terminal module of METTL16 is essential for charge-based binding to RNA
- METTL16 preferentially methylates adenosines within structured RNAs
- Regulation of *Mat2a* mRNA by *Mettl16* is essential for mouse embryonic development

Mendel et al., 2018, Molecular Cell *71*, 986–1000
September 20, 2018 © 2018 The Authors. Published by Elsevier Inc.
https://doi.org/10.1016/j.molcel.2018.08.004

CellPress
Molecular Cell
Article

Methylation of Structured RNA by the m^{6}A Writer METTL16 Is Essential for Mouse Embryonic Development

Mateusz Mendel,[1,3] Kuan-Ming Chen,[1,3] David Homolka,[1] Pascal Gos,[1] Radha Raman Pandey,[1] Andrew A. McCarthy,[2] and Ramesh S. Pillai[1,4,*]
[1]Department of Molecular Biology, Science III, University of Geneva, 30 Quai Ernest-Ansermet, CH-1211 Geneva 4, Switzerland
[2]European Molecular Biology Laboratory, Grenoble Outstation, 71 Avenue des Martyrs, 38042 Grenoble, France
[3]These authors contributed equally
[4]Lead Contact
*Correspondence: ramesh.pillai@unige.ch
https://doi.org/10.1016/j.molcel.2018.08.004

SUMMARY

Internal modification of RNAs with *N*^{6}-methyladenosine (m^{6}A) is a highly conserved means of gene expression control. While the METTL3/METTL14 heterodimer adds this mark on thousands of transcripts in a single-stranded context, the substrate requirements and physiological roles of the second m^{6}A writer METTL16 remain unknown. Here we describe the crystal structure of human METTL16 to reveal a methyltransferase domain furnished with an extra N-terminal module, which together form a deep-cut groove that is essential for RNA binding. When presented with a random pool of RNAs, METTL16 selects for methylation-structured RNAs where the critical adenosine is present in a bulge. Mouse 16-cell embryos lacking *Mettl16* display reduced mRNA levels of its methylation target, the SAM synthetase *Mat2a*. The consequence is massive transcriptome dysregulation in ~64-cell blastocysts that are unfit for further development. This highlights the role of an m^{6}A RNA methyltransferase in facilitating early development via regulation of SAM availability.

INTRODUCTION

Methylation of adenosines at the *N*^{6} position (*N*^{6}-methyladenosine or m^{6}A) is a highly conserved internal RNA modification with a huge impact on gene regulation (Fu et al., 2014). The modification is added by methyltransferase "writers" and can be removed by RNA demethylase "erasers," and a major part of its functions is mediated by YTH domain "reader" proteins that can recognize the m^{6}A mark. Readers of the m^{6}A modification are shown to modulate mRNA splicing, RNA export, RNA stability, and translation (Patil et al., 2018). Alterations in RNA structure are also a consequence of m^{6}A methylation (Liu et al., 2015). The m^{6}A pathway is physiologically important, as mutations in the writer protein METTL3 in mice lead to embryonic lethality (Batista et al., 2014; Geula et al., 2015), while in flies it affects sex determination (Haussmann et al., 2016; Lence et al., 2016). The only nuclear reader protein YTHDC1 is essential for early embryonic development, and its conditional deletion causes infertility in the germline where it acts via modulation of splicing and alternative polyadenylation site usage (Kasowitz et al., 2018). Loss of the cytoplasmic reader YTHDF2 in fish impairs embryonic development as a result of defective maternal RNA clearance during maternal-zygotic transition (Zhao et al., 2017), while in mice loss of YTHDF2 results in defective maternal RNA metabolism during oocyte maturation, leading to female-specific infertility (Ivanova et al., 2017). In contrast, mouse YTHDC2 is essential for proper progression through meiosis and fertility in both sexes (Bailey et al., 2017; Hsu et al., 2017; Jain et al., 2018; Wojtas et al., 2017). Thus, gene regulation by m^{6}A plays a critical role in a variety of developmental processes.

The heterodimeric m^{6}A writer complex METTL3/METTL14 co-transcriptionally (Knuckles et al., 2017; Slobodin et al., 2017) installs this mark on thousands of transcripts in the cell (Dominissini et al., 2012; Schwartz et al., 2013). While METTL3 is the active component, METTL14 facilitates substrate RNA binding (Śledź and Jinek, 2016; Wang et al., 2016a, 2016b). METTL16 is the second m^{6}A methyltransferase identified, and its known substrates include U6 snRNA and the human *MAT2A* mRNA that encodes for *S*-adenosylmethionine (SAM) synthetase (Pendleton et al., 2017). SAM is a methyl donor for methylation reactions in the cell, including those of DNA, RNA, and protein. While METTL3 prefers to methylate single-stranded RNAs (ssRNAs) in a sequence context RR<u>A</u>CH (R = A or G; H = A,C or U), METTL16 uses structured RNAs carrying a specific nonamer sequence (UAC<u>A</u>GAGAA; methylated adenosine is underlined) (Pendleton et al., 2017). Methylation of *MAT2A* mRNA within specific hairpin structures in the 3′ UTR is proposed to be used by YTHDC1 to mediate downregulation of the mRNA under high-SAM conditions (Shima et al., 2017). Apart from this enzymatic role, METTL16 is also reported to act as a splicing enhancer during low-SAM conditions when it occupies its binding site on the six *MAT2A* hairpins (hp) to promote splicing of a 3′ terminal intron that is frequently retained. This results in increased mature *MAT2A* mRNA production and acts as a feedback loop ensuring optimal production of the SAM synthetase in

 © 2018 The Authors. Published by Elsevier Inc.
This is an open access article under the CC BY license (http://creativecommons.org/licenses/by/4.0/).

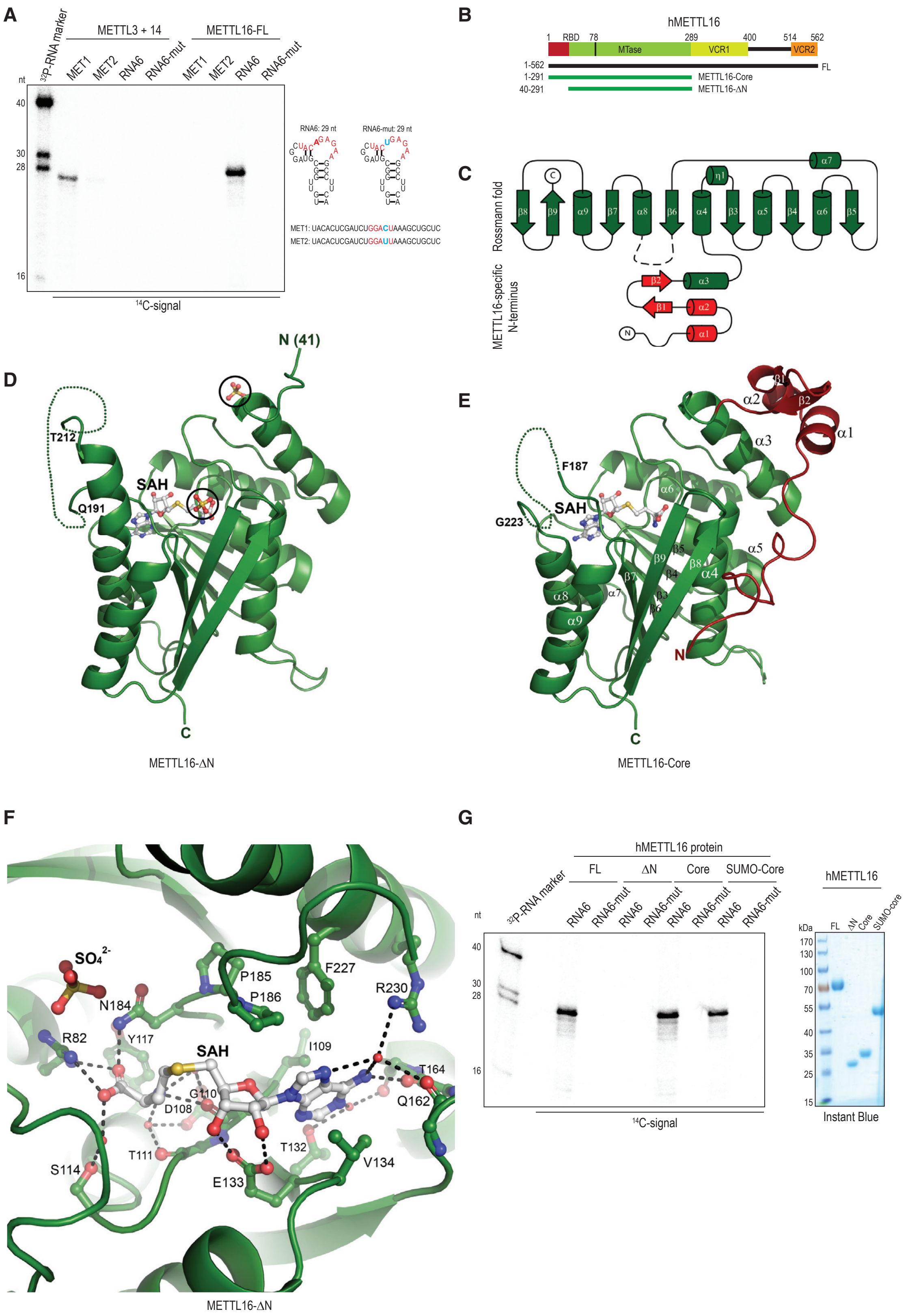

(legend on next page)

response to low SAM levels (Pendleton et al., 2017). Unlike the METTL3/METTL14 complex which mainly methylates exonic sequences (Ke et al., 2017), METTL16 was shown to have binding sites on several intronic sequences in pre-mRNAs and structured noncoding RNAs, some of which carry m^{6}A marks (Brown et al., 2016; Warda et al., 2017). How METTL16 recognizes its RNA substrates and the physiological importance of having a second m^{6}A methyltransferase is currently not known.

METTL16 is a highly conserved enzyme with orthologs found in *E.coli* (Sergiev et al., 2008) to human (Figure S1A). Here, we examine the crystal structure of the methyltransferase (MTase) domain from human METTL16 and identify key features that are essential for RNA binding and methylation activity. We define the RNA substrate requirements *in vitro* using a randomized RNA library to find that structured RNAs with a bulged adenosine are preferred. Finally, we generate a knockout *Mettl16* mouse mutant to show that the protein is essential for early embryonic development. Our studies show that METTL16 is essential for embryonic development around implantation stage and acts via regulation of the *Mat2a* mRNA which encodes the SAM synthetase.

RESULTS

Crystal Structures of the Human m^{6}A Methyltransferase METTL16

We produced the recombinant full-length (FL) human METTL3/METTL14 heterodimeric complex and FL human METTL16 (1–562 aa) in a eukaryotic expression system (Figures S1B and S1C; Star Methods). Together with the methyl donor *S*-adenosylmethionine (SAM), the enzymes were presented with either a single-stranded RNA (ssRNA, MET1) carrying the RR**A**CH consensus site or a 29 nt hairpin RNA (RNA6) derived from the human *MAT2A* mRNA, carrying the nonamer methylation site for METTL16 (UAC**A**GAGAA) (Table S1) (Pendleton et al., 2017). While the METTL3/METTL14 complex efficiently methylated the ssRNA, it did not use the hairpin RNA as a substrate (Figure 1A). On the contrary, METTL16-FL methylated only the hairpin substrate, but not the ssRNA. Both enzymes also sensed the sequence context of their respective substrates, as single nucleotide mutations within the RNA consensus sites either reduced (for METTL3/14 complex) or abolished (for METTL16) the methylation activity (Figure 1A). The METTL16-FL protein was also capable of using U6 snRNA and the full-length *MAT2A* hairpin (hp) 1 as substrates for methylation (Figure S1D). Thus, the purified m^{6}A methyltransferases are able to discriminate their respective RNA substrates *in vitro*.

To obtain structural information on METTL16, we identified stable protein domains by limited proteolysis (Figure S1E). Two constructs (core, 1–291 aa; and ΔN, 40–291 aa) encompassing the methyltransferase domain (MTase) were expressed in *E. coli* and crystallized (Star Methods) (Figures 1B and S1B). Consistent with the SAM-dependent methyltransferase activity of METTL16, both structures reveal a Rossmann fold composed of a central seven-stranded β sheet (strands β3–β9) flanked by three α helices each (helices α4–α6 and α7–α9) (Figures 1C–1E). The complexed byproduct of the SAM-dependent methylation reaction, *S*-adenosyl homocysteine (SAH), is coordinated by a hydrogen bond interaction network with the highly conserved (Figure S1A) amino acid residues R82, D108, G110, T111, S114, E133, Q162, N184, and R230 (Figures 1F, S1G, and S2A). The methyl donor SAM is presumed to fit into the same surface-exposed pocket. The catalytic residues NPPF (184–187 aa) are present in a loop positioned in close proximity to the SAH molecule (Figures 1E, 1F, and S2A). Point mutations (PP185–186AA or F187G) of these residues abolish m^{6}A RNA methyltransferase activity on a *MAT2A* hairpin substrate when tested *in vitro* (Figures 2D and S2D). All these key features define METTL16 as a SAM-dependent methyltransferase.

Three additional observations can be made from our structures. First, and most striking, is the presence of an N-terminal module (1–78 aa) in the core structure that is appended to the α4 of the Rossmann fold and that consists of three helices (α1–α3) and two short β strands (β1 and β2) (Figures 1C and 1E). This module is flexible, as shown by proteolysis, with only α3 remaining in the ΔN structure (Figures 1D and S1E). Second, the loop containing the catalytic residues NPPF (N184–F187) is part of a larger stretch of 35 amino acids that links β6 to α8, and it is disordered in both structures (dotted lines in Figures 1D and 1E). While 20 residues between Q191 and T212 are not visible in the ΔN structure, a much larger region (35 residues) between F187 and G223 lacks density in the core structure (Figures

Figure 1. Structure of Human METTL16 Reveals an N-Terminal Module Essential for Activity

(A) *In vitro* methylation assays of indicated full-length (FL) human m^{6}A methyltransferases with ^{14}C-SAM and different RNA substrates (right). Predicted structure of a short hairpin RNA (RNA6) derived from the longer *MAT2A* hairpin 1 (Pendleton et al., 2017) and its mutant (RNA6-mut) with A→U mutation of the methylated adenosine are shown. The MET1 RNA has the consensus site for methylation by the METTL3+METTL14 complex, while the MET2 RNA has a point mutation (C→U) of a conserved residue in the methylation consensus site (see Table S1). Single-stranded RNA markers (length in nucleotides, nt) are ^{32}P-end-labeled. See also Figure S1D.

(B) Domain architecture of human METTL16. RBD, RNA-binding domain (1–78 aa); MTase, methyltransferase domain; VCR, vertebrate conserved region. Boundaries of the two protein constructs crystallized in this study are indicated (in green). The ΔN version has an N-terminal deletion.

(C) Schematic view of the MTase domain. Cylinders represent α helices, and arrows represent β strands. Regions shaded in red (α1-2 and β1-2) are seen only in the METTL16-core structure and together with α3 form a separate N-terminal module.

(D) Model of the METTL16-ΔN construct (PDB 6GFK). Two-sulfate (SO_4^{2-}) ions visualized in the crystal structure are circled. A disordered loop between α8 and β6 is connected by a dotted line. SAH, S-adenosyl homocysteine.

(E) Model of the METTL16-core construct (PDB 6GFN). The additional regions at the N terminus seen in this structure are shown in red. See also Figure S1G.

(F) A zoom of the catalytic pocket in the METTL16-ΔN structure showing coordination of SAH. Catalytic residues N184, P185, P186, and F187 and position of a sulfate (SO_4^{2-}) ion are indicated. See also Figure S2A.

(G) *In vitro* methylation assays showing that METTL16-ΔN protein is inactive. The METTL16-Core protein was used as untagged or tagged (SUMO) versions. See also Figure S2C. Quality of proteins used is shown on the right. Protein molecular weight markers (in kilo Daltons, kDa) are indicated.

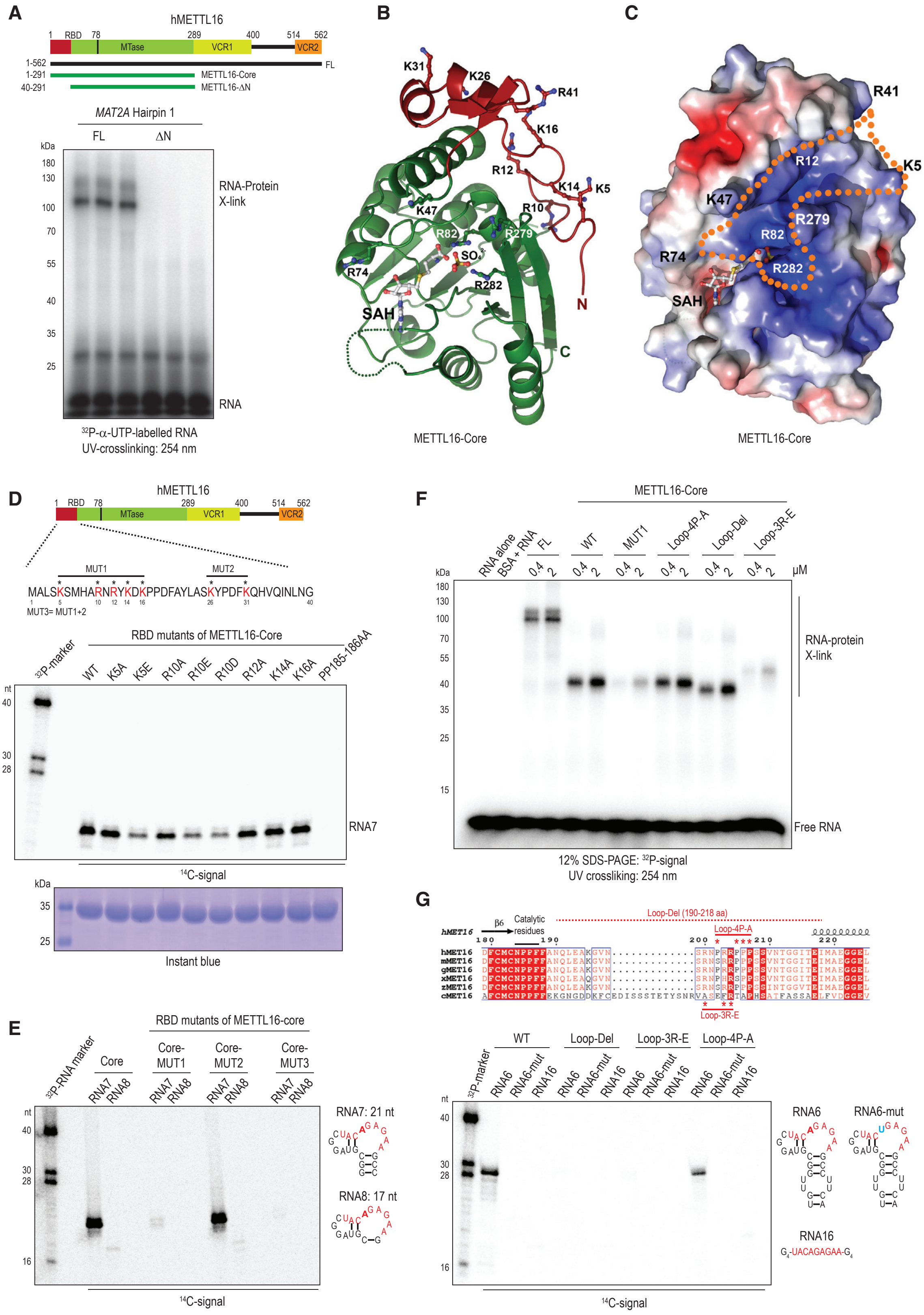

(legend on next page)

1D and 1E). This loop in *C. elegans* is even longer (48 aa) and shows poor overall sequence conservation with its vertebrate orthologs (Figure S1A). Given its strategic location, it may be involved in contacting the bound RNA substrate during catalysis. Third, the ΔN structure reveals the presence of two sulfate ions (SO_4^{2-}) (Figure 1D), one of which is next to R82 (one of the residues coordinating SAH), likely mimicking how an RNA substrate might access the catalytic pocket (Figure 1F).

To test whether these structures are representative of catalytically active versions of the METTL16 MTase domain, we incubated the recombinant proteins with ^{14}C-SAM and a 29 nt RNA (RNA6) derived from the *MAT2A* hp 1. The full-length METTL16 and the core domain versions were able to methylate this RNA (RNA6) at a specific adenosine residue (A17) within the nonamer UAC**A**GAGAA motif (Figure 1G). In contrast, a mutant hairpin (RNA6-mut) with A17U mutation was not methylated. To our surprise, even though the ΔN-truncated version (40–291 aa) has a similar conformation in terms of the Rossmann fold and catalytic residues, it was inactive (Figure 1G). A similar situation is seen even when the full-length *MAT2A* hairpin 1 is used (Figure S2C). Taken together, our two structures reveal an architecture where the MTase domain is attached to a METTL16-specific N-terminal module that is essential for activity.

The N-Terminal Module of METTL16 Is Essential for RNA Binding, while a Disordered Loop Is Required for Catalysis

Given that the METTL16-ΔN domain is inactive as an MTase, we examined whether it can bind RNA substrates using UV crosslinking experiments. Incubation of METTL16-FL with a body-labeled *MAT2A* hp 1 RNA gave an RNA-protein crosslink consistent with RNA binding, while the ΔN version did not reveal such an interaction (Figure 2A). This aligns with the observed lack of methylation activity of the ΔN protein when the same RNA substrate was used (Figure S2C). Examination of the primary sequence of the N-terminal module revealed the presence of several highly conserved positively charged residues that can potentially be involved in mediating interaction with RNAs (Figure S1A). Indeed, when mapped onto the METTL16-core structure, these reveal a positively charged cluster (K5, R10, R12, K14, K16, and R41) that forms the entrance of a wide deep-cut groove (Figures 2B and 2C). With additional contributions from the N-terminal module (K47 and R74) and those from within the Rossmann fold (R82, R279, and R282), the groove runs all the way to the catalytic pocket containing the bound SAH (Figures 2B and 2C). The residues K47 and R279 serve to constrict the space within this groove, while R74 overlooks the ridge that surrounds the SAH-binding pocket. Crucially, R82 and R282, that are centrally located close to the SAH molecule itself, coordinate one of the negatively charged sulfate ions (SO_4^{2-}) that we found in the ΔN structure (Figures 1D and 2C), potentially mimicking how an RNA substrate might position itself on the enzyme.

To directly examine the role of these N-terminal residues in RNA-binding and hence catalytic activity, we individually converted positively charged residues to neutral alanine. Interestingly, mutant METTL16-core versions carrying the single point mutations K5A, R10A, R12A, K14A, and K16A did not affect RNA methylation activity (Figures 2D and S2D). However, individual mutations into a negatively charged residue (K5E, R10E, and R10D) had a more discernible impact by reducing RNA methylation activity (Figure 2D). These individual mutations did not completely abolish activity as seen in the catalytic-dead mutant PP185-186AA, indicating that these might merely reduce RNA binding. Strikingly, a combined mutant with all five residues converted to alanine (MUT1: K5A, R10A, R12A, K14A, and K16A) completely eliminated RNA methylation activity (Figure 2E). In contrast, a combined mutation of charged residues not lining the potential RNA-binding groove (MUT2: K26A and K31A) (Figure 2B) did not affect RNA methylation activity (Figure 2E). Consistently, a further mutant (MUT3) containing all the mutations made in MUT1 and MUT2 did not show any activity. To examine RNA binding, we performed UV crosslinking experiments (Figure 2F). As expected, the full-length METTL16 and the METTL16-core version bound the 5′ end-labeled RNA. In contrast, the METTL16-core version carrying the combined MUT1 mutations showed highly reduced binding. These results provide a structural rationale for absence of RNA binding and RNA methylation activity in the METTL16-ΔN protein.

Figure 2. The N-Terminal Module of Human METTL16 Is Required for Substrate RNA Binding

(A) Domain architecture of human METTL16. UV crosslinking assay (triplicate reactions) showing RNA-protein crosslinks (X-link) between human full-length (FL) METTL16 and *MAT2A* hairpin 1 RNA. See also Figure S2C for *in vitro* methylation with the same proteins and RNA.

(B) Overview of the METTL16-core MTase domain. Key positively charged residues that create the RNA-binding groove are indicated. Note that residues K26 and K31 when mutated (MUT2) do not affect activity. SAH, S-adenosyl homocysteine; SO_4^{2-}, position of a sulfate ion as seen in METTL16-ΔN is shown. The disordered loop with catalytic residues is shown as a dotted line.

(C) Surface charge representation of the METTL16-core MTase domain showing a positively charged (blue) groove (outlined) leading from the N terminus to the catalytic pocket.

(D) Cartoon showing the N-terminal 40 amino acids of human METTL16, with the highlighted positively charged residues that were mutated (red, with asterisks). Gel shows the *in vitro* methylation assay with wild-type (WT) or indicated point mutant METTL16-core proteins. Quality of recombinant proteins used is shown below the gel. RNA7 was used as an RNA substrate (see Table S1). Single-stranded RNA markers (length in nucleotides, nt) are ^{32}P-end-labeled. See also Figure S2D.

(E) *In vitro* methylation assay with RNAs indicated and mutants carrying multiple point mutations on the N-terminal module (see D). See also Figure S2E for additional mutations within the RNA binding groove.

(F) UV crosslinking assay with METTL16 proteins indicated and ^{32}P-end-labeled RNA6. The positions of the free RNA and RNA-protein crosslinks (X-link) are shown. Control binding reactions are carried out without any protein (RNA alone) or with bovine serum albumin (BSA).

(G) Sequence alignment of METTL16 orthologs showing the catalytic residues and disordered loop region. See also Figure S1A. Deletions and mutations introduced into the loop in the context of the METTL16-core construct are indicated. *In vitro* methylation assay with indicated proteins and RNAs is shown below. Quality of proteins used is shown in Figure S2F.

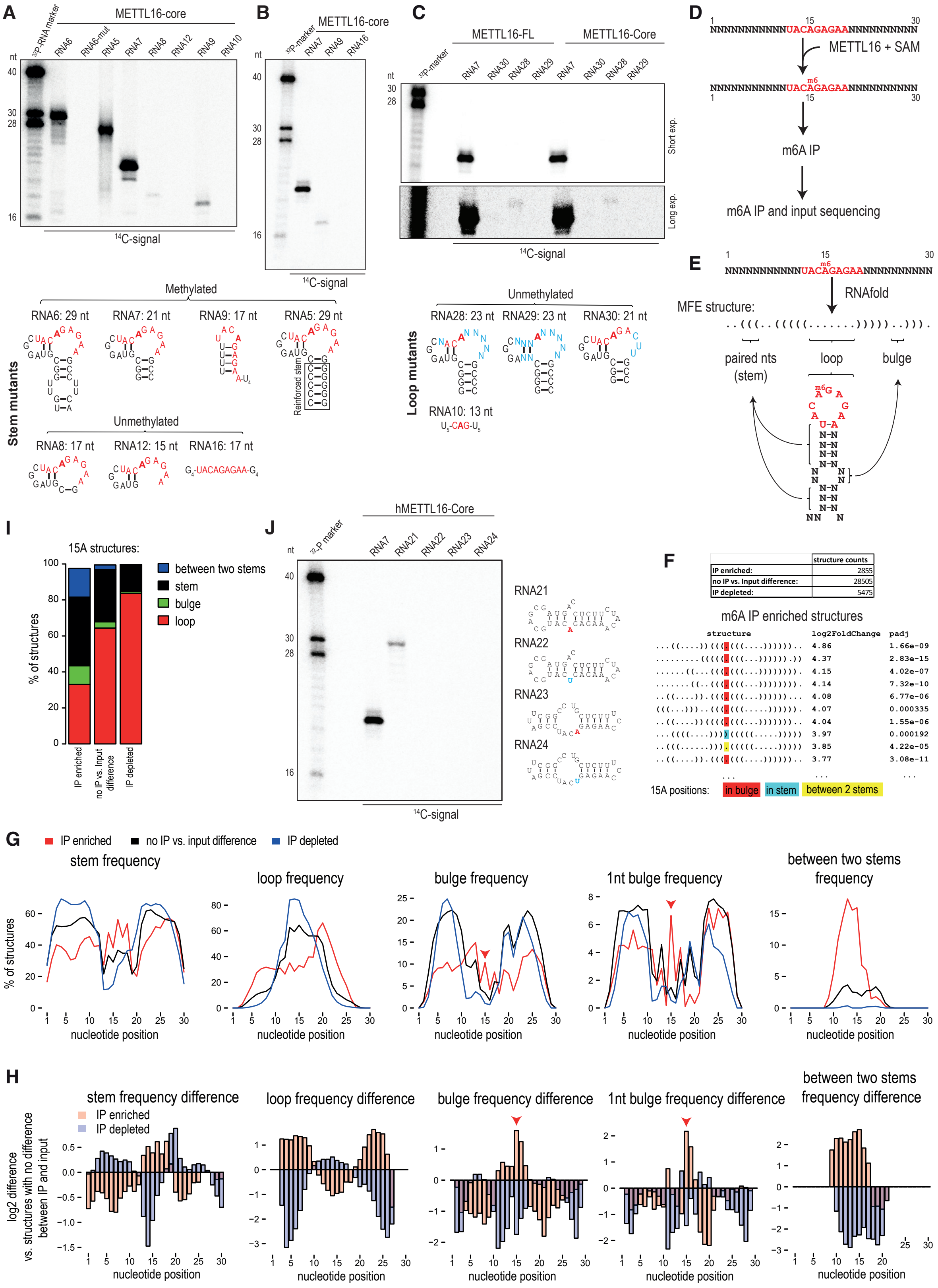

(legend on next page)

We extended the mutational analyses to the other positively charged residues lining the putative RNA-binding groove. Mutation of the residues K47 and R279 that form a claw-like constriction of the groove either reduces (in the case of K47E) or abolishes (in R279E or double mutant K47E+R279E) methylation activity (Figure S2E). Mutation of other residues R82E, R282E, and R74E also abolishes activity, confirming their involvement in construction of the putative RNA-binding groove.

Next, we probed the importance of the disordered loop containing the catalytic residues (Figure 2G). Confirming its critical role, deletion of most of the loop (190–218 aa) abolishes *in vitro* methylation. In fact, loss of methylation can be reproduced by just three point mutations (Loop-3R-E: RRR-200-203-204-EEE) converting positive charges to negative residues, while mutation of four prolines (Loop-4P-A) within the loop did not affect methylation activity (Figure 2G). Interestingly, deletion of the disordered loop did not have an adverse effect on RNA binding, as measured by UV crosslinking (Figure 2F). However, the Loop3R-E mutant displayed highly reduced RNA binding, probably due to repulsion of RNA. These results indicate that the loop, per se, is not required for RNA binding, but has a potential catalytic role by directly contacting the RNA for proper positioning within the catalytic pocket.

In conclusion, our structure-informed mutagenesis study traces an RNA-binding groove lined by positively charged residues contributed by the N-terminal module and the MTase domain itself. This facilitates RNA binding, and thus promotes RNA methylation activity. In addition, we identify a disordered loop that is essential for catalysis.

METTL16 Prefers Structured RNAs as Substrates for m^{6}A Methylation *In Vitro*

The two known methylation targets of METTL16 are structured RNAs: the U6 snRNA and *MAT2A* hp 1 (Pendleton et al., 2017; Warda et al., 2017). To identify the RNA features that can allow *in vitro* methylation by METTL16, we carried out truncations/mutations of the *MAT2A* hp1. The *MAT2A* hp RNAs with reductions in the stem region beyond three base pairs fail to get methylated (Figures 3A, S2B, S3A, and S3B) (Pendleton et al., 2017). Activity can be restored by an artificial six base pair C/G stem (RNA5), indicating the critical requirement for any stem region (Figures 3A and S1D). On the other hand, presence of the nonamer motif in a single-stranded context, when flanked by a run of Gs (RNA16), did not support methylation (Figure 3B). Interestingly, an RNA where the nonamer motif is flanked by a run of Us (RNA9) is a substrate (albeit weak) for METTL16, likely because it has the potential to form a structured feature (Figures 3A, 3B, and S3B). To test the importance of the nonamer itself, we introduced three mutations within this motif (RNA30), which abolished activity (Figure 3C). Additional mutations, in the form of randomizations of the motif sequences (RNA28 and RNA29), also abolished activity (Figure 3C). Lastly, an RNA with only the central C**A**G flanked by Us (RNA10) was inactive, reaffirming the importance of this nonamer motif for methylation (Figure 3A). Taken together, these studies indicate that the nonamer sequence in the context of a secondary structure feature is essential for METTL16 methylation activity *in vitro*.

To probe the secondary structure requirements in an unbiased manner, we carried out *in vitro* methylation reactions with recombinant full-length METTL16 and a library of randomized 30 nt single-stranded RNAs carrying a central nonamer motif (Figure 3D). Subsequently, a part was retained as input while the rest was used for immunoprecipitation (IP) of methylated RNAs with the anti-m^{6}A antibody (Figure 3D). Sequences were identified by deep sequencing and preferred secondary structures of these sequences were examined using RNAfold (STAR Methods; Table S2) (Figure 3E). We identified $\sim$2,800 predicted structures (referring to unique dot bracket notations) to be enriched in the m^{6}A-IP library (Figure 3F).

To find out whether specific structural features are important for methylation, we focused on selected secondary structural features (i.e., stems, loops, bulges, and nucleotides lying between two stems) and compared the frequencies of these features for individual nucleotide positions between the m^{6}A-enriched, non-enriched, and m^{6}A-depleted structures (Figures 3G and 3H), or directly between the sequenced oligos (Figures S3C and S3D). Analysis indicates that the nonamer sequence motif of m^{6}A-enriched structures occurs with a higher frequency in a paired stem region. However, the 15th nt adenosine that is expected to be methylated within the motif is often not paired

Figure 3. METTL16 Requires Structured RNA for m^{6}A Methylation

(A) *In vitro* methylation assay with RNAs carrying truncations of the stem region. See also Figure S3A. The predicted structures of RNAs used are shown below.

(B) *In vitro* methylation assay.

(C) *In vitro* methylation assay with RNAs carrying mutations in the nonamer consensus sequence (shown below). Short and long exposures of the gel are shown.

(D) Scheme of an *in vitro* methylation experiment using a library of randomized (N = any of the four nucleotides) RNA oligos.

(E) For each sequence, we predicted the minimum free energy (MFE) secondary structure using RNAfold (STAR Methods). A model structure is shown in dot bracket notation.

(F) The representation of individual structures (corresponding to unique dot bracket notation) was compared between m^{6}A-IP samples and input samples. Top ten IP-enriched structures are shown. The 15th position adenosine (A) that is in the consensus nonamer sequence is highlighted.

(G) Frequency of structures forming stem, loop and other selected features at individual positions is shown. The IP-enriched structures have increased frequency of 15A (red arrowhead) in a bulge and surrounded by double-stranded regions (stems), pointing to specific structural requirements of RNA substrates for METTL16.

(H) Structures enriched or depleted in m^{6}A IP were compared to those that do not show such difference (between IP and input). While the IP-enriched structures have higher proportion of 15A (red arrowhead) forming a bulge or lying between two stems, the IP-depleted structures show the opposite trend, with lack of structures with 15A in a bulge or in between two stems.

(I) The barplot shows the proportion of structural features in which the 15A was found. Note the high proportion of structures with 15A in the bulge and between two stems, among the m^{6}A IP-enriched structures. See also Figure S3.

(J) *In vitro* methylation assay with METTL16-core protein and RNAs (RNA21 and RNA23) selected from randomized library methylation experiment (D). This confirms the specific methylation of 15A which is in a 1 nt bulge (in RNA21, but not in mutant RNA22).

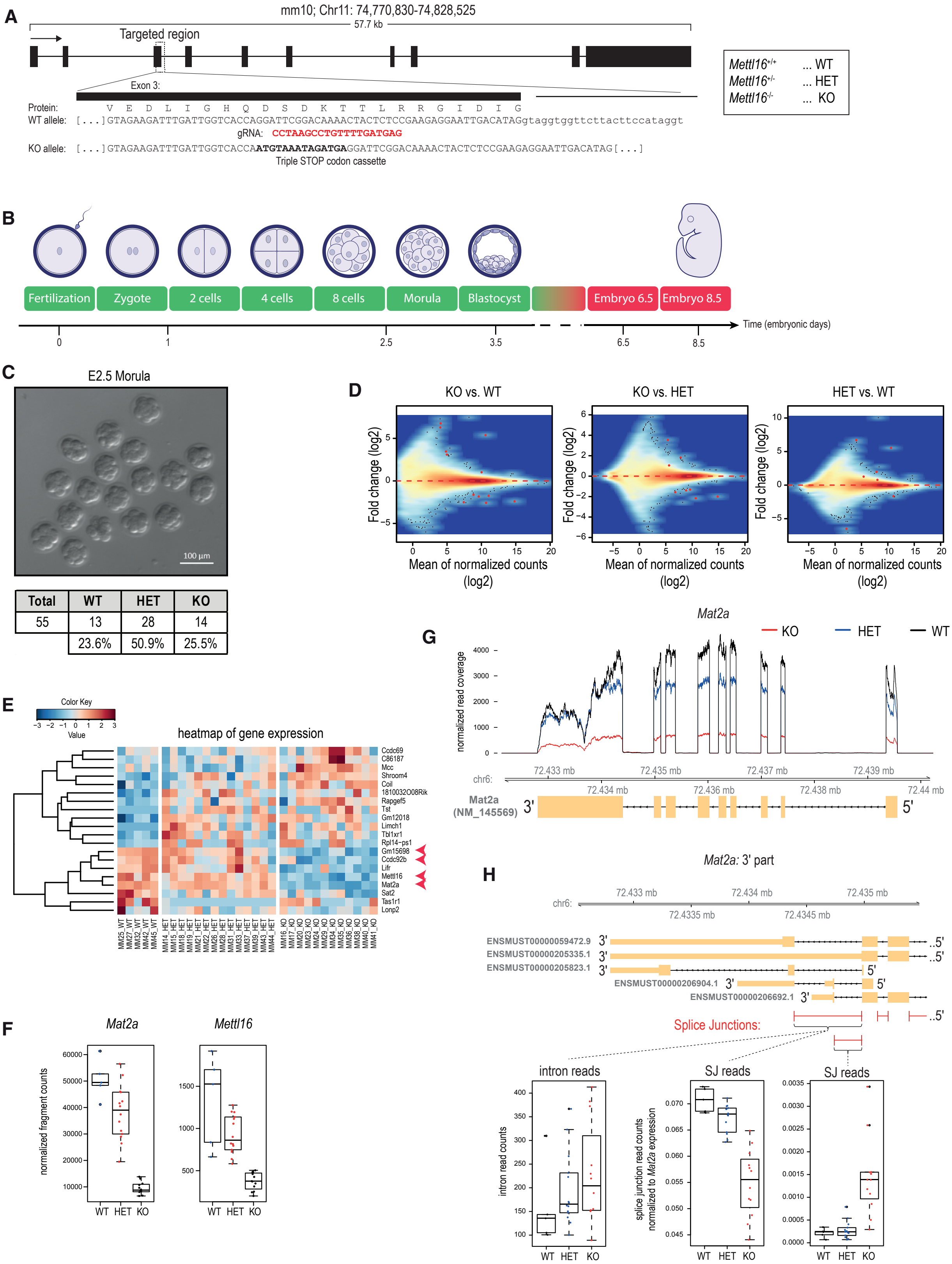

Total	WT	HET	KO
55	13	28	14
	23.6%	50.9%	25.5%

(legend on next page)

and more frequently present in a 1 nt bulge. There is also a higher frequency for the unpaired 15A to lie between two stems (Figures 3G–3I). m^{6}A IP-enriched and IP-depleted sequences show an opposing trend affirming the importance of an unpaired adenosine surrounded by local double-strands for methylation (Figure 3H). Indeed, an RNA (RNA 21) representative of the structure enriched in the m^{6}A-IP (15th position adenosine in a bulge between two stems) supports methylation by METTL16 (Figure 3J). This is specific as methylation is abolished when the 15th position A is mutated to U (RNA22) (Figure 3J). However, another RNA (RNA23) did not show any methylation activity (Figure 3J). These results show that the nonamer motif does not necessarily have to adopt a loop structure for activity but that the target adenosine must be unpaired and surrounded by stems. Taken together, we reveal that besides the sequence motif there is a structural requirement for a nonamer to serve as a substrate for METTL16 methylation.

Mouse METTL16 Regulates Embryonic *Mat2a* mRNA Levels and This Is Essential for Embryonic Development

To uncover the endogenous targets of METTL16, we created a knockout allele (*Mettl16*$^-$) by inserting a triple-stop codon cassette into exon 3 of the mouse *Mettl16* genomic locus (Figures 4A, S4A, and S4B; STAR Methods). Heterozygous (HET) *Mettl16*$^{+/-}$ mice of both sexes are viable and fertile. Intercrosses between them provided litters that were completely devoid of any homozygous (KO) *Mettl16*$^{-/-}$ animals, indicating potential embryonic lethality (Figure S4C).

After fertilization of the oocyte by the sperm, the 1-cell zygote goes through mitotic divisions to take it through a totipotent 2-cell stage, followed later by the pluripotent 16-cell morula seen at embryonic day 2.5 (E2.5) and 32- to 64-cell blastocyst at E3.5. Subsequently, the embryo becomes implanted in the uterine wall and proceeds into post-implantation development (Figure 4B). To examine the embryonic arrest in the *Mettl16* mutant, we first isolated E2.5 morula from superovulated *Mettl16*$^{+/-}$ females crossed with *Mettl16*$^{+/-}$ males (Figure 4C). Visual examination revealed no apparent differences in the embryos at this stage. Examination of E3.5 blastocysts resulted in a similar conclusion (Figure 5A). Indeed, genotyping of individual embryos confirmed the presence of all genotypes in the expected Mendelian ratios at both E2.5 and E3.5 (Figures 4C and 5A). However, examination of post-implantation embryos at E8.5 and E12.5 indicated a total absence of the knockout genotype, but E6.5 KO embryos (1.9%) were detected at sub-Mendelian ratios (Figures S4D–S4F). These results indicate that the *Mettl16*$^{-/-}$ knockout mutation allows embryonic development until blastocyst stage but causes developmental arrest around the time of implantation.

To evaluate the impact of loss of METTL16, we sequenced the transcriptomes of individual 16-cell morulas at E2.5 originating from heterozygous *Mettl16*$^{+/-}$ crosses. Embryos were genotyped based on presence or absence of specific *Mettl16* reads (STAR Methods), and gene expression levels were compared between different genotypes (Figure 4D). We find 20 genes to be differentially expressed between the different genotypes (WT, HET, and KO) (Figure 4E; Table S3). However, only four genes are consistently different in the KO embryos when compared to both WT and HET (marked with red arrowheads in Figures 4E and S5B). Examination of transcript changes in the individual embryos reveals an expected and consistent downregulation of *Mettl16* in the KO embryos (Figure 4F). Strikingly, the most significantly dysregulated transcript was *Mat2a*, which displays a 5-fold downregulation in the KO embryos. Two additional transcripts *Ccdc92b* and *Gm15698* also display significant downregulation in the KO embryos (Figure S5C).

Identification of *Mat2a* as a downregulated transcript in the *Mettl16* knockout embryos is interesting, as it is already an established target of METTL16 in human cell lines (Pendleton et al., 2017). METTL16 was proposed to promote splicing of the terminal intron, failure of which leads to intron-retention and transcript degradation (Pendleton et al., 2017). Examination of the read count distribution over the exons and introns of *Mat2a* reveals that while the exonic reads are consistently decreased in the KO, we did not observe any dramatic change in intronic reads (Figures 4G and S5D). The same was true for the two other transcripts downregulated in the KO embryos (Figure S5E). Nevertheless, a closer examination around the terminal intron of the *Mat2a* indicates a differential usage of splice junctions in the KO accompanied by a slight increase in the terminal intronic

Figure 4. Reduced *Mat2a* mRNA Levels and Embryonic Lethality around Implantation Stage in the *Mettl16* Mutant Mice

(A) Generation of a *Mettl16* knockout (KO) allele. See also Figure S4A and STAR Methods.

(B) Timeline of mouse embryogenesis. Embryonic day 2.5 (E2.5) embryos referring to 16-cell morula stage, E3.5 blastocysts, and E6.5 and E8.5 embryos were collected for genotyping. KO embryos were detected in expected Mendelian ratios till E3.5 (colored in green), but at sub-Mendelian ratios at E6.5 or none beyond (colored in red). See also Figures S4C–S4F.

(C) Genotyping of E2.5 embryos from *Mettl16*$^{+/-}$ x *Mettl16*$^{+/-}$ crosses confirmed the expected Mendelian ratios among the genotypes. Scale bar in μm is indicated.

(D) Transcriptome of individual isolated E2.5 embryos of *Mettl16*$^{-/-}$ (KO), *Mettl16*$^{+/-}$ (HET), and *Mettl16*$^{+/+}$ (WT) was sequenced and compared between the genotypes. The MA plots show a very limited number of differentially expressed genes (red dots, adjusted $p \leq 0.1$). See also Figure S5.

(E) Heatmap shows the expression of genes with significant differential expression between any two genotypes (adjusted $p \leq 0.1$). Genes differentially expressed in *Mettl16*$^{-/-}$(KO) when compared to both *Mettl16*$^{+/-}$ (HET) and *Mettl16*$^{+/+}$ (WT) are marked by red arrowhead.

(F) The boxplots show the expected downregulation of the targeted gene (*Mettl16*) in KO samples, as well as the downregulation of *Mat2a*. Transcript levels of individual samples are shown as dots. See also Figure S5C.

(G) Normalized read coverage along the *Mat2a* locus demonstrates the overall depletion in the KO. Note that the gene is on the Crick strand, so it goes from right to left.

(H) Lack of METTL16 results in aberrant splicing of the last intron. The reads spanning the splice junction (SJ) of last *Mat2a* (ENSMUST00000059472.9) intron are significantly depleted in the KO even when normalized to overall *Mat2a* transcript levels. This is accompanied by slight increase for intron reads and increased usage of alternative 3′ splice-site characteristic for the ENSMUST00000206904.1 and ENSMUST00000206692.1 variants. See also Figure S5D.

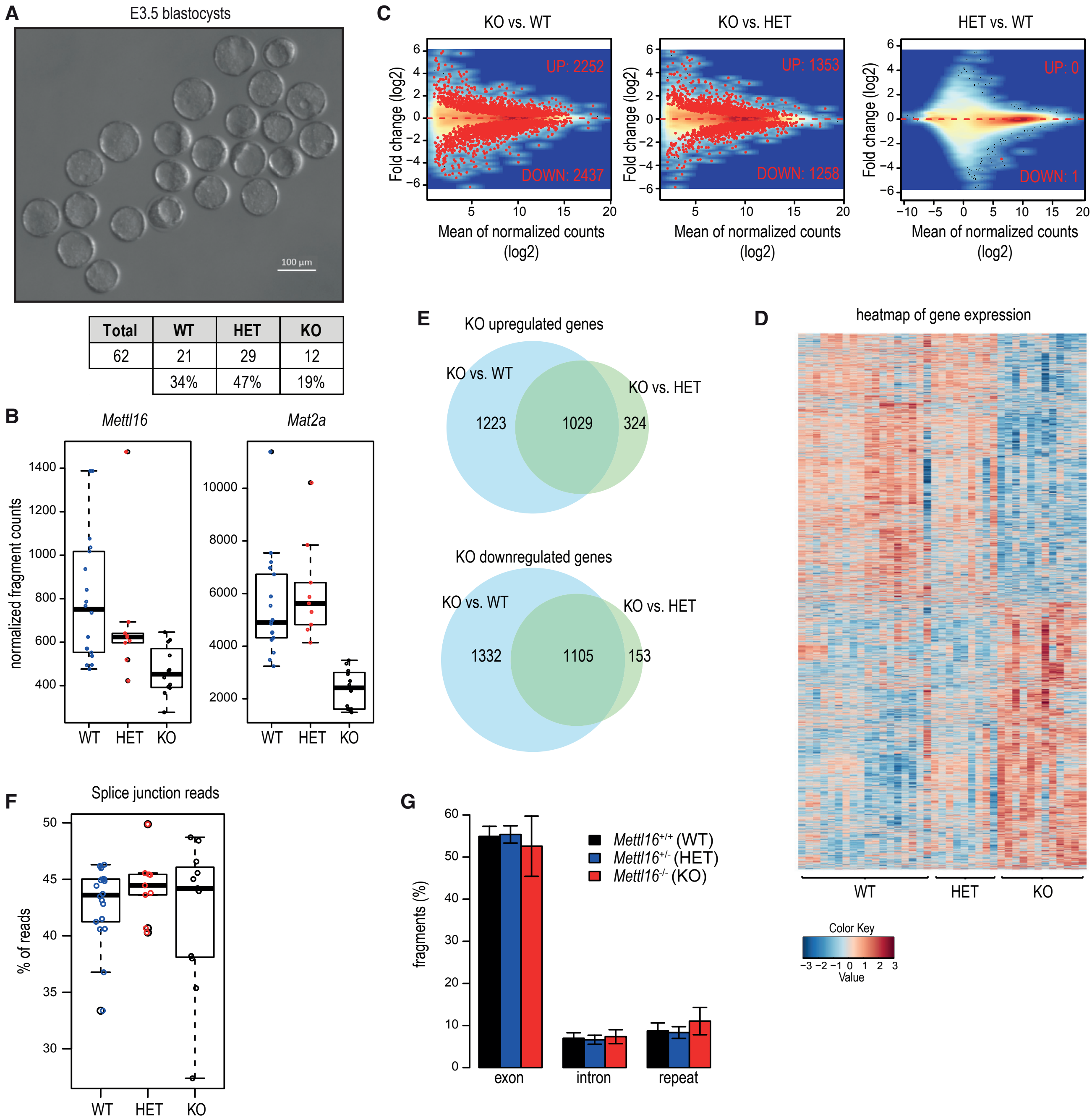

Figure 5. E3.5 *Mettl16*$^{-/-}$ Blastocysts Display Normal Morphology but Vast Transcriptome Dysregulation

(A) E3.5 *Mettl16*$^{-/-}$ KO embryos display normal morphology and their counts from *Mettl16*$^{+/-}$ x *Mettl16*$^{+/-}$ crosses correspond to expected Mendelian ratios among the genotypes. Scale bar in μm is indicated.

(B) The boxplots show the expected downregulation of the targeted gene (*Mettl16*) in KO samples, as well as the downregulation of *Mat2a*. Transcript levels of individual samples are shown as dots. See also Figure S6A.

(C) MA plots comparing the expression between the genotypes reveal that the vast number of genes are dysregulated in the *Mettl16*$^{-/-}$ KO embryos. The genes with significantly different expression are shown as red dots (adjusted $p \leq 0.1$).

(D) Heatmap shows the expression of 5,166 genes with significant differential expression between any two genotypes (adjusted $p \leq 0.1$). Note the massive dysregulation in the KO embryos. See also Figures S6B–S6D.

(E) Venn diagrams compare the lists of dysregulated genes when *Mettl16*$^{-/-}$ expression is compared to *Mettl16*$^{+/-}$ or to *Mettl16*$^{+/+}$.

(F) Comparison of proportion of reads encompassing splice junctions does not reveal a difference in splicing between individual genotypes.

(G) Global transcription from exons, introns, and repeats is not affected in *Mettl16*$^{-/-}$. Error bars refer to SD.

reads (Figure 4H). In conclusion, we demonstrate that *Mettl16* is essential for viability of early mouse embryos where it regulates the levels of *Mat2a* mRNA.

Loss of METTL16 Leads to Dramatic Alterations in the E3.5 Blastocyst Transcriptome

To examine whether the downregulation of very few transcripts in E2.5 embryos has further consequences in the E3.5 blastocysts, we collected such embryos from superovulated *Mettl16*$^{+/-}$ females crossed with *Mettl16*$^{+/-}$ males (Figure 5A). Sequencing of single embryos revealed the expected downregulation of *Mettl16* and *Mat2a* (Figure 5B). Strikingly, ~5,000 other transcripts were either upregulated or downregulated in the KO, when compared to the WT embryos, while up to half that number was altered in the KO versus HET comparison (Figures 5C, 5D, and S6D). Examination of these altered-gene lists indicates that up to 1,000 genes are either commonly up- or downregulated in the KO when compared to both WT and HET embryos (Figure 5E). A previous study identified key transcription and chromatin factors that define specific developmental stage transcriptomes (Mohammed et al., 2017). Examination of these factors in our datasets did not reveal any altered expression profile between the genotypes (Figures S6B and S6C). A Gene Ontology (GO) term analysis of the altered transcripts revealed an upregulation in splicing-related factors (Table S3), but analysis of splice junction reads did not reveal any changes in the KO embryos (Figure 5F). We also did not observe any dramatic changes in the representation of exon, intron, and repeat reads in the different libraries (Figure 5G). Taken together, even though the molecular effect of the loss of METTL16 is already seen in E2.5 embryos in the form of reduced mRNA levels of its methylation target *Mat2a*, its consequences are amplified in the E3.5 KO embryos. Here, a massive dysregulation of gene expression is observed, such that mutant embryos undergoing implantation are doomed to fail in further development.

DISCUSSION

Crystal structures now reveal how two RNA methyltransferases are built to recognize distinct RNA targets and install the same m^{6}A mark. The two methyltransferase (MTase) domains in the heterodimeric METTL3/METTL14 complex interact to create a narrow groove lined with conserved positively charged residues into which single-stranded RNAs can fit (Śledź and Jinek, 2016; Wang et al., 2016a, 2016b). This interaction facilitates stabilization of a large "interface loop" in METTL3 that contributes to the catalytic activity. Indeed, this ensures that METTL3, which binds SAM, is not active on its own, requiring at least the MTase domain of METTL14 to complete the creation of a functional catalytic complex (Śledź and Jinek, 2016; Wang et al., 2016a). In contrast, we show here that METTL16 is active as a monomer (Figures 1 and S1B), and it contains a large deep-cut groove that can accommodate structured RNAs (Figure 2). Interestingly, the METTL3/METTL14 crystal complex with the two MTase domains is inactive and requires the two N-terminal CCCH zinc finger motifs of METTL3 to recover methylation activity (Śledź and Jinek, 2016; Wang et al., 2016a), presumably because it aids in substrate RNA binding. Similarly, here we demonstrate that the N-terminal module attached to the MTase of METTL16 is essential for RNA-binding and catalysis (Figures 1 and 2). We note that the recently reported crystal structure of the human METTL16 core MTase domain (PDB 6B92) (Ruszkowska et al., 2018) shows a high degree of overlap with the one studied here (Figure S1F; Star Methods).

A structural comparison of the human METTL3/METTL14 complex (PDB 5IL2) with that of our human METTL16-core (PDB 6GFN) reveals similarity to METTL3 in the overall Rossmann fold (Figure 6A). It also shows how the disordered loop in METTL16 (Figures 1E and 2G) is very similar to the "gate loop 1" in METTL3, as both harbor the catalytic residues and are likely involved in contacting the bound RNA during enzymatic reaction. Our mutational studies indicate that the disordered loop in METTL16 is not required for RNA binding (Loop-Del in Figure 2F) but is essential for catalytic activity (Figure 2G). Thus, its role might be to contact the substrate bound via the RNA-binding groove and orient it for catalysis. This is supported by our finding that mutation of positively charged arginine (R) residues in the loop to glutamic acid (E) abolishes RNA binding (Figure 2F), perhaps via charge repulsions. We modeled a structured RNA (tRNA from PDB 2ZZM) into this groove, and it shows how an unpaired adenosine in the loop region might reach into the catalytic pocket for methylation (Figure 6B). To get better insight into the catalytic mechanism, we modeled a methyl-acceptor adenosine (from PDB 4ZCF, chain B) (Gupta et al., 2015) into the SAH binding site of METTL16-core (PDB 6GFN) (Figure 6C). Superimposition of the METTL16-core structure with that of the m^{6}A DNA MTase, EcoP1GI (PDB 4ZCF, chain B), reveals how the adenosine is favorably positioned by coordination with catalytic residues N184 and P185 for the methyl transfer from SAM (represented by SAH in Figure 6C). However, our experiments do not reveal how METTL16 might be able to recognize an adenosine within a specific nonamer sequence for m^{6}A methylation. This information will be forthcoming only when structures with bound RNA become available.

Regulation of gene expression by m^{6}A is essential at multiple steps during mouse embryonic development. The writer *Mettl3* is essential for embryonic development, with *Mettl3*-deficient embryonic stem cells (ESCs) failing to exit pluripotency despite differentiation cues (Batista et al., 2014; Geula et al., 2015; Wang et al., 2014). Now we show that the writer *Mettl16* is also essential for embryonic development around implantation stage (Figures 4 and 5). Our biochemical studies and *in vivo* transcriptome profiling reveals severe sequence and structural constraints on potential RNA targets of METTL16 (Figures 3 and 4). Although a few hundred transcripts carrying the nonamer sequence motif exist in the mouse genome, we did not find any differences in their levels in *Mettl16* knockout E2.5 embryos (Figure 4). This reinforces our finding that a combination of sequence and structural features define the target set for METTL16. The fact that *Mat2a* is the sole main target of METTL16 in pre-implantation embryos is interesting, as it encodes for the SAM synthetase, which produces SAM, the main methyl donor required for many methylation reactions (including DNA, protein, and RNA methylation) with huge regulatory potential. Before implantation, the embryonic genome undergoes massive erasure of DNA methylation marks, while during post-implantation development,

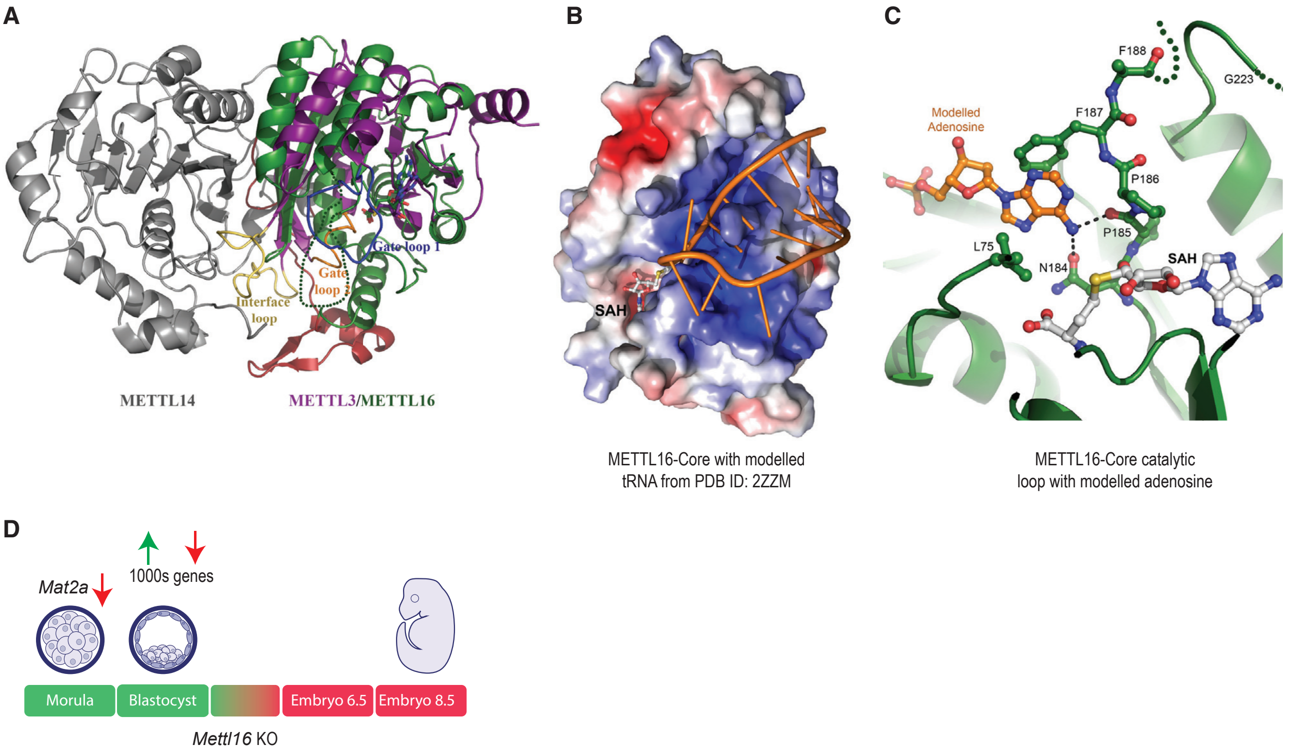

Figure 6. A Model for METTL16 Function during Early Embryonic Development

(A) Structural comparison of METTL16 core and METTL3/METTL14 complex. METTL3 (PDB 5IL2), colored purple, was superimposed on the METTL16-core-SAH (PDB 6GFN), colored in green (core) and red (N-terminal). Gate loops 1 and 2, and the interface loop of METTL3, are colored in blue, orange, and yellow, respectively. The disordered loop in our METTL16-core is shown as a dotted line.

(B) Surface charge representation of human METTL16-core domain with a modeled tRNA (from PDB: 2ZZM). See STAR Methods. The SAH bound in the catalytic pocket is shown.

(C) A methyl-acceptor adenosine (orange) was modeled into the SAH binding site of METTL16 core (PDB 6GFN) by superimposition of an m^{6}A DNA MTase, EcoP151 (PDB: 4ZCF, chain B). The sulfate binding site (as in Figure 1F) overlaps with the adenosine base moiety.

(D) A model summarizing the physiological role of METTL16 during early mouse development. The downregulation of the SAM synthetase *Mat2a* mRNA in *Mettl16* KO E2.5 morula is potentially a trigger for subsequent massive alteration in gene expression in the E3.5 blastocysts. Such mutant embryos fail to proceed further in development (indicated in red).

DNA methylation increases and is restored back to normal levels (Reik et al., 2001). Given our finding that the transcriptome in the E3.5 embryos is massively dysregulated (Figure 5), we propose that it is a snowballing effect of the initial downregulation of *Mat2a*. The low levels of *Mat2a* mRNA will mean that downstream epigenetic reprogramming events are also bound to fail. Such E3.5 mutant blastocysts are unfit for continuing in development (Figure 6D). In this context, it is interesting to point out that a homozygous *Mat2a* knockout mutation results in embryonic lethality in mice (International Mouse Phenotyping Consortium [IMPC]). Furthermore, chemical inhibition of bovine MAT2A enzyme in cultured bovine pre-implantation embryos also reduced blastocyst development (Ikeda et al., 2017).

How might METTL16 function to stabilize *Mat2a* mRNA in the mouse embryos? METTL16-mediated methylation of the hairpin structures in the 3′ UTR in *Mat2a* mRNA is used by YTHDC1 to promote its decay in high-SAM conditions (Shima et al., 2017). Thus in the absence of *Mettl16* we would have expected a stabilization of the transcript. Perhaps an explanation might come from the proposed non-canonical function of METTL16 as a splicing enhancer (Pendleton et al., 2017), where it promotes splicing to remove the 3′ terminal intron to create a stable mature *MAT2A* mRNA during low-SAM conditions. Based on this model, loss of *Mettl16* leads to reduced splicing of the terminal exon, resulting in intron-retained unstable transcripts and hence not detected in our sequencing experiments (Figures 4 and 5). Consistently, we detected higher levels of intronic reads in the *Mettl16* knockout embryos (Figure 4H). The C-terminal vertebrate conserved regions (VCRs) of METTL16 are proposed to mediate this activity (Pendleton et al., 2017). We speculate that some of the splicing factors we identified in endogenous METTL16 complexes from mouse tissues, and from transfected human cell lines, may participate in this role (Figures S5F and S5G). Future studies using a catalytic-dead METTL16 mutant mouse should help settle this issue of methylation-mediated decay versus splicing role. However, it is also possible that multiple pathways co-exist to control *Mat2a* levels.

HeLa cell extracts were originally shown to harbor an activity that adds an m^{6}A mark at position A43 in the splicing machinery component U6 snRNA (Shimba et al., 1995). This activity was

later identified to be METTL16 (Pendleton et al., 2017; Warda et al., 2017). Mutation of this methylation site in yeast U6 snRNA, which lies within a region that base pairs with the 5′ splice site of pre-mRNAs, causes lethality (Madhani et al., 1990). However, our analysis of the *Mettl16* knockout mouse embryos did not reveal any gross changes in splicing patterns across the transcriptome (Figures S5A and 5G). Thus, it is possible that methylation of U6 snRNA is not critical for splicing in higher organisms, perhaps due to the diversity of 5′ splice site sequences (Yang et al., 2013). Alternatively, complementation by another MTase activity in our mutant might account for lack of splicing defects. In conclusion, our studies place the m^{6}A writer METTL16 in a dominant position to influence early developmental decisions in the mouse embryo via regulation of SAM synthetase expression.

STAR★METHODS

Detailed methods are provided in the online version of this paper and include the following:

- KEY RESOURCES TABLE
- CONTACT FOR REAGENT AND RESOURCE SHARING
- EXPERIMENTAL MODEL AND SUBJECT DETAILS
  - Animal Work
  - *Mettl16* knockout mice
  - Genotyping
  - Mouse embryos
- METHOD DETAILS
  - Clones and constructs
  - Antibodies
  - Recombinant protein production
  - Purification of METTL3-METTL14 complex
  - Purification of METTL16
  - Limited proteolysis of hMETTL16-FL
  - Crystallization and data collection
  - Structure determination and refinement
  - *In vitro* transcription of RNA substrates for methylation assay
  - *In vitro* RNA methylation assay with METTL16
  - UV crosslinking assay
  - Cell culture and transfections
  - Isolation of human METTL16 complexes for mass spectrometry
  - Isolation of METTL16 complexes from mouse testes and spleen
  - Mass spectrometry
  - Preparation of RNA libraries
- QUANTIFICATION AND STATISTICAL ANALYSIS
  - *In vitro* methylation with METTL16 and m^{6}A-IP-RNaseq
  - Transcriptome analysis of Mettl16 mutant mouse embryos
- DATA AND SOFTWARE AVAILABILITY

SUPPLEMENTAL INFORMATION

Supplemental Information includes six figures and three tables and can be found with this article at https://doi.org/10.1016/j.molcel.2018.08.004.

ACKNOWLEDGMENTS

We thank Olivier Fazio and Leonardo Beccari for help with mouse embryo collection, Margot Riggi for scientific illustrations, Magdalena Wojtas for the METTL3/14 protein complex, and Johanna Mattay for critical reading of the manuscript. We thank the following facilities: iGE3 Genomics Platform, University of Geneva, and the EMBL Genomics core facility for sequencing; the Transgenic Mouse Facility, University of Geneva for mouse creation; the Functional Genomics Center, Zurich for proteomics analyses. M.M. is grateful for a PhD Fellowship from the Boehringer Ingelheim Fonds. This work was supported by grants to R.S.P. from the Swiss National Science Foundation: ERC Transfer Grant (GermMethylation), Project Grant (Origin-of-pi), and funding from the NCCR RNA & Disease. Work in the Pillai lab is supported by the Republic and Canton of Geneva.

AUTHOR CONTRIBUTIONS

M.M. performed all biochemical and mouse experiments with help from P.G.; K.-M.C produced recombinant proteins and performed structural analyses with A.A.M.; R.R.P. prepared the *Mettl16* knockout mouse and sequencing libraries; D.H. conducted all computational analysis; manuscript preparation and writing was by R.S.P. with input from everyone.

DECLARATION OF INTERESTS

The authors declare no competing interests.

Received: May 7, 2018
Revised: June 22, 2018
Accepted: July 30, 2018
Published: September 6, 2018

REFERENCES

Bailey, T.L., and Elkan, C. (1994). Fitting a mixture model by expectation maximization to discover motifs in biopolymers. Proc. Int. Conf. Intell. Syst. Mol. Biol. *2*, 28–36.

Bailey, A.S., Batista, P.J., Gold, R.S., Chen, Y.G., de Rooij, D.G., Chang, H.Y., and Fuller, M.T. (2017). The conserved RNA helicase YTHDC2 regulates the transition from proliferation to differentiation in the germline. eLife *6*, e26116.

Batista, P.J., Molinie, B., Wang, J., Qu, K., Zhang, J., Li, L., Bouley, D.M., Lujan, E., Haddad, B., Daneshvar, K., et al. (2014). m(6)A RNA modification controls cell fate transition in mammalian embryonic stem cells. Cell Stem Cell *15*, 707–719.

Bieniossek, C., Imasaki, T., Takagi, Y., and Berger, I. (2012). MultiBac: expanding the research toolbox for multiprotein complexes. Trends Biochem. Sci. *37*, 49–57.

Bricogne, G., Blanc, E., Brandl, M., Flensburg, C., Keller, P., Paciorek, W., Roversi, P., Sharff, A., Smart, O.S., Vonrhein, C., et al. (2016). BUSTER (Global Phasing Ltd.).

Brown, J.A., Kinzig, C.G., DeGregorio, S.J., and Steitz, J.A. (2016). Methyltransferase-like protein 16 binds the 3′-terminal triple helix of MALAT1 long noncoding RNA. Proc. Natl. Acad. Sci. USA *113*, 14013–14018.

Chen, V.B., Arendall, W.B., 3rd, Headd, J.J., Keedy, D.A., Immormino, R.M., Kapral, G.J., Murray, L.W., Richardson, J.S., and Richardson, D.C. (2010). MolProbity: all-atom structure validation for macromolecular crystallography. Acta Crystallogr. D Biol. Crystallogr. *66*, 12–21.

Chen, E.Y., Tan, C.M., Kou, Y., Duan, Q., Wang, Z., Meirelles, G.V., Clark, N.R., and Ma'ayan, A. (2013). Enrichr: interactive and collaborative HTML5 gene list enrichment analysis tool. BMC Bioinformatics *14*, 128.

Dobin, A., Davis, C.A., Schlesinger, F., Drenkow, J., Zaleski, C., Jha, S., Batut, P., Chaisson, M., and Gingeras, T.R. (2013). STAR: ultrafast universal RNA-seq aligner. Bioinformatics *29*, 15–21.

Dolinsky, T.J., Nielsen, J.E., McCammon, J.A., and Baker, N.A. (2004). PDB2PQR: an automated pipeline for the setup of Poisson-Boltzmann electrostatics calculations. Nucleic Acids Res. *32*, W665-7.

Dominissini, D., Moshitch-Moshkovitz, S., Schwartz, S., Salmon-Divon, M., Ungar, L., Osenberg, S., Cesarkas, K., Jacob-Hirsch, J., Amariglio, N., Kupiec, M., et al. (2012). Topology of the human and mouse m6A RNA methylomes revealed by m6A-seq. Nature *485*, 201–206.

Emsley, P., Lohkamp, B., Scott, W.G., and Cowtan, K. (2010). Features and development of Coot. Acta Crystallogr. D Biol. Crystallogr. *66*, 486–501.

Flot, D., Mairs, T., Giraud, T., Guijarro, M., Lesourd, M., Rey, V., van Brussel, D., Morawe, C., Borel, C., Hignette, O., et al. (2010). The ID23-2 structural biology microfocus beamline at the ESRF. J. Synchrotron Radiat. *17*, 107–118.

Fu, Y., Dominissini, D., Rechavi, G., and He, C. (2014). Gene expression regulation mediated through reversible m^6A RNA methylation. Nat. Rev. Genet. *15*, 293–306.

Geula, S., Moshitch-Moshkovitz, S., Dominissini, D., Mansour, A.A., Kol, N., Salmon-Divon, M., Hershkovitz, V., Peer, E., Mor, N., Manor, Y.S., et al. (2015). Stem cells. m6A mRNA methylation facilitates resolution of naïve pluripotency toward differentiation. Science *347*, 1002–1006.

Goto-Ito, S., Ito, T., Kuratani, M., Bessho, Y., and Yokoyama, S. (2009). Tertiary structure checkpoint at anticodon loop modification in tRNA functional maturation. Nat. Struct. Mol. Biol. *16*, 1109–1115.

Gupta, Y.K., Chan, S.H., Xu, S.Y., and Aggarwal, A.K. (2015). Structural basis of asymmetric DNA methylation and ATP-triggered long-range diffusion by EcoP15I. Nat. Commun. *6*, 7363.

Hahne, F., and Ivanek, R. (2016). Visualizing genomic data using Gviz and Bioconductor. Methods Mol. Biol. *1418*, 335–351.

Hartley, S.W., and Mullikin, J.C. (2016). Detection and visualization of differential splicing in RNA-Seq data with JunctionSeq. Nucleic Acids Res. *44*, e127.

Haussmann, I.U., Bodi, Z., Sanchez-Moran, E., Mongan, N.P., Archer, N., Fray, R.G., and Soller, M. (2016). m^6A potentiates Sxl alternative pre-mRNA splicing for robust Drosophila sex determination. Nature *540*, 301–304.

Hsu, P.J., Zhu, Y., Ma, H., Guo, Y., Shi, X., Liu, Y., Qi, M., Lu, Z., Shi, H., Wang, J., et al. (2017). Ythdc2 is an N^6-methyladenosine binding protein that regulates mammalian spermatogenesis. Cell Res. *27*, 1115–1127.

Huber, W., Carey, V.J., Gentleman, R., Anders, S., Carlson, M., Carvalho, B.S., Bravo, H.C., Davis, S., Gatto, L., Girke, T., et al. (2015). Orchestrating high-throughput genomic analysis with Bioconductor. Nat. Methods *12*, 115–121.

Ikeda, S., Kawahara-Miki, R., Iwata, H., Sugimoto, M., and Kume, S. (2017). Role of methionine adenosyltransferase 2A in bovine preimplantation development and its associated genomic regions. Sci. Rep. *7*, 3800.

Ivanova, I., Much, C., Di Giacomo, M., Azzi, C., Morgan, M., Moreira, P.N., Monahan, J., Carrieri, C., Enright, A.J., and O'Carroll, D. (2017). The RNA m(6)A reader YTHDF2 is essential for the post-transcriptional regulation of the maternal transcriptome and oocyte competence. Mol. Cell *67*, 1059–1067.

Jain, D., Puno, M.R., Meydan, C., Lailler, N., Mason, C.E., Lima, C.D., Anderson, K.V., and Keeney, S. (2018). *ketu* mutant mice uncover an essential meiotic function for the ancient RNA helicase YTHDC2. eLife *7*, e30919.

Kabsch, W. (2010). Xds. Acta Crystallogr. D Biol. Crystallogr. *66*, 125–132.

Kasowitz, S.D., Ma, J., Anderson, S.J., Leu, N.A., Xu, Y., Gregory, B.D., Schultz, R.M., and Wang, P.J. (2018). Nuclear m6A reader YTHDC1 regulates alternative polyadenylation and splicing during mouse oocyte development. PLoS Genet. *14*, e1007412.

Ke, S., Alemu, E.A., Mertens, C., Gantman, E.C., Fak, J.J., Mele, A., Haripal, B., Zucker-Scharff, I., Moore, M.J., Park, C.Y., et al. (2015). A majority of m6A residues are in the last exons, allowing the potential for 3′ UTR regulation. Genes Dev. *29*, 2037–2053.

Ke, S., Pandya-Jones, A., Saito, Y., Fak, J.J., Vågbø, C.B., Geula, S., Hanna, J.H., Black, D.L., Darnell, J.E., Jr., and Darnell, R.B. (2017). m^6A mRNA modifications are deposited in nascent pre-mRNA and are not required for splicing but do specify cytoplasmic turnover. Genes Dev. *31*, 990–1006.

Knuckles, P., Carl, S.H., Musheev, M., Niehrs, C., Wenger, A., and Bühler, M. (2017). RNA fate determination through cotranscriptional adenosine methylation and microprocessor binding. Nat. Struct. Mol. Biol. *24*, 561–569.

Kuleshov, M.V., Jones, M.R., Rouillard, A.D., Fernandez, N.F., Duan, Q., Wang, Z., Koplev, S., Jenkins, S.L., Jagodnik, K.M., Lachmann, A., et al. (2016). Enrichr: a comprehensive gene set enrichment analysis web server 2016 update. Nucleic Acids Res. *44* (W1), W90–W97.

Lence, T., Akhtar, J., Bayer, M., Schmid, K., Spindler, L., Ho, C.H., Kreim, N., Andrade-Navarro, M.A., Poeck, B., Helm, M., and Roignant, J.Y. (2016). m^6A modulates neuronal functions and sex determination in Drosophila. Nature *540*, 242–247.

Liao, Y., Smyth, G.K., and Shi, W. (2014). featureCounts: an efficient general purpose program for assigning sequence reads to genomic features. Bioinformatics *30*, 923–930.

Liu, N., Dai, Q., Zheng, G., He, C., Parisien, M., and Pan, T. (2015). N(6)-methyladenosine-dependent RNA structural switches regulate RNA-protein interactions. Nature *518*, 560–564.

Lorenz, R., Bernhart, S.H., Höner Zu Siederdissen, C., Tafer, H., Flamm, C., Stadler, P.F., and Hofacker, I.L. (2011). ViennaRNA Package 2.0. Algorithms Mol. Biol. *6*, 26.

Love, M.I., Huber, W., and Anders, S. (2014). Moderated estimation of fold change and dispersion for RNA-seq data with DESeq2. Genome Biol. *15*, 550.

Madhani, H.D., Bordonné, R., and Guthrie, C. (1990). Multiple roles for U6 snRNA in the splicing pathway. Genes Dev. *4* (12B), 2264–2277.

McCarthy, A.A., Barrett, R., Beteva, A., Caserotto, H., Dobias, F., Felisaz, F., Giraud, T., Guijarro, M., Janocha, R., Khadrouche, A., et al. (2018). ID30B—a versatile beamline for macromolecular crystallography experiments at the ESRF. J. Synchrotron Radiat. *25*, 1249–1260.

McCoy, A.J., Grosse-Kunstleve, R.W., Adams, P.D., Winn, M.D., Storoni, L.C., and Read, R.J. (2007). Phaser crystallographic software. J. Appl. Cryst. *40*, 658–674.

Mohammed, H., Hernando-Herraez, I., Savino, A., Scialdone, A., Macaulay, I., Mulas, C., Chandra, T., Voet, T., Dean, W., Nichols, J., et al. (2017). Single-cell landscape of transcriptional heterogeneity and cell fate decisions during mouse early gastrulation. Cell Rep. *20*, 1215–1228.

Morin, A., Eisenbraun, B., Key, J., Sanschagrin, P.C., Timony, M.A., Ottaviano, M., and Sliz, P. (2013). Collaboration gets the most out of software. eLife *2*, e01456.

Patil, D.P., Pickering, B.F., and Jaffrey, S.R. (2018). Reading m^6A in the transcriptome: m^6A-binding proteins. Trends Cell Biol. *28*, 113–127.

Patro, R., Duggal, G., Love, M.I., Irizarry, R.A., and Kingsford, C. (2017). Salmon provides fast and bias-aware quantification of transcript expression. Nat. Methods *14*, 417–419.

Pendleton, K.E., Chen, B., Liu, K., Hunter, O.V., Xie, Y., Tu, B.P., and Conrad, N.K. (2017). The U6 snRNA m(6)A methyltransferase METTL16 regulates SAM synthetase intron retention. Cell *169*, 824–835.

Picelli, S., Faridani, O.R., Björklund, A.K., Winberg, G., Sagasser, S., and Sandberg, R. (2014). Full-length RNA-seq from single cells using Smart-seq2. Nat. Protoc. *9*, 171–181.

R Core Team (2017). R: A Language and Environment for Statistical Computing (Vienna, Austria: R Foundation for Statistical Computing).

Reik, W., Dean, W., and Walter, J. (2001). Epigenetic reprogramming in mammalian development. Science *293*, 1089–1093.

Ruszkowska, A., Ruszkowski, M., Dauter, Z., and Brown, J.A. (2018). Structural insights into the RNA methyltransferase domain of METTL16. Sci. Rep. *8*, 5311.

Schwartz, S., Agarwala, S.D., Mumbach, M.R., Jovanovic, M., Mertins, P., Shishkin, A., Tabach, Y., Mikkelsen, T.S., Satija, R., Ruvkun, G., et al. (2013). High-resolution mapping reveals a conserved, widespread, dynamic mRNA methylation program in yeast meiosis. Cell *155*, 1409–1421.

Sergiev, P.V., Serebryakova, M.V., Bogdanov, A.A., and Dontsova, O.A. (2008). The ybiN gene of Escherichia coli encodes adenine-N6 methyltransferase

specific for modification of A1618 of 23 S ribosomal RNA, a methylated residue located close to the ribosomal exit tunnel. J. Mol. Biol. *375*, 291–300.

Shima, H., Matsumoto, M., Ishigami, Y., Ebina, M., Muto, A., Sato, Y., Kumagai, S., Ochiai, K., Suzuki, T., and Igarashi, K. (2017). S-Adenosylmethionine synthesis is regulated by selective N^6-adenosine methylation and mRNA degradation involving METTL16 and YTHDC1. Cell Rep. *21*, 3354–3363.

Shimba, S., Bokar, J.A., Rottman, F., and Reddy, R. (1995). Accurate and efficient N-6-adenosine methylation in spliceosomal U6 small nuclear RNA by HeLa cell extract in vitro. Nucleic Acids Res. *23*, 2421–2426.

Śledź, P., and Jinek, M. (2016). Structural insights into the molecular mechanism of the m(6)A writer complex. eLife *5*, e18434.

Slobodin, B., Han, R., Calderone, V., Vrielink, J.A., Loayza-Puch, F., Elkon, R., and Agami, R. (2017). Transcription impacts the efficiency of mRNA translation via co-transcriptional N6-adenosine methylation. Cell *169*, 326–337.

Soneson, C., Love, M.I., and Robinson, M.D. (2015). Differential analyses for RNA-seq: transcript-level estimates improve gene-level inferences. F1000Res. *4*, 1521.

Tickle, I.J., Flensburg, C., Keller, P., Paciorek, W., Sharff, A., Vonrhein, C., and Bricogne, G. (2017). STARANISO (Global Phasing Ltd.).

Vonrhein, C., Flensburg, C., Keller, P., Sharff, A., Smart, O., Paciorek, W., Womack, T., and Bricogne, G. (2011). Data processing and analysis with the autoPROC toolbox. Acta Crystallogr. D Biol. Crystallogr. *67*, 293–302.

Wang, Y., Li, Y., Toth, J.I., Petroski, M.D., Zhang, Z., and Zhao, J.C. (2014). N6-methyladenosine modification destabilizes developmental regulators in embryonic stem cells. Nat. Cell Biol. *16*, 191–198.

Wang, P., Doxtader, K.A., and Nam, Y. (2016a). Structural basis for cooperative function of Mettl3 and Mettl14 methyltransferases. Mol. Cell *63*, 306–317.

Wang, X., Feng, J., Xue, Y., Guan, Z., Zhang, D., Liu, Z., Gong, Z., Wang, Q., Huang, J., Tang, C., et al. (2016b). Structural basis of N(6)-adenosine methylation by the METTL3-METTL14 complex. Nature *534*, 575–578.

Warda, A.S., Kretschmer, J., Hackert, P., Lenz, C., Urlaub, H., Höbartner, C., Sloan, K.E., and Bohnsack, M.T. (2017). Human METTL16 is a N^6-methyladenosine (m^6A) methyltransferase that targets pre-mRNAs and various non-coding RNAs. EMBO Rep. *18*, 2004–2014.

Wojtas, M.N., Pandey, R.R., Mendel, M., Homolka, D., Sachidanandam, R., and Pillai, R.S. (2017). Regulation of m6A transcripts by the $3' \rightarrow 5'$ RNA helicase YTHDC2 is essential for a successful meiotic program in the mammalian germline. Mol. Cell *68*, 374–387.

Yang, F., Wang, X.Y., Zhang, Z.M., Pu, J., Fan, Y.J., Zhou, J., Query, C.C., and Xu, Y.Z. (2013). Splicing proofreading at 5′ splice sites by ATPase Prp28p. Nucleic Acids Res. *41*, 4660–4670.

Zhao, B.S., Wang, X., Beadell, A.V., Lu, Z., Shi, H., Kuuspalu, A., Ho, R.K., and He, C. (2017). m^6A-dependent maternal mRNA clearance facilitates zebrafish maternal-to-zygotic transition. Nature *542*, 475–478.

STAR★METHODS

KEY RESOURCES TABLE

REAGENT or RESOURCE	SOURCE	IDENTIFIER
Antibodies		
Polyclonal rabbit anti-m^{6}A	Synaptic Systems	Cat. no. 202003; RRID:AB_2279214
Polyclonal rabbit anti-METT10D	Abcam	Cat. no. ab186012
Mouse IgG control antibody	Santa Cruz	Cat. no. sc-2025; RRID:AB_737182
Bacterial and Virus Strains		
DH10EMBacY bacterial strain	(Bieniossek et al., 2012)	N/A
Biological Samples		
PMSG	MSD Animal Health	Folligon
HCG	MSD Animal Health	Chorulon
Chemicals, Peptides, and Recombinant Proteins		
Sodium deoxycholate	Sigma	30968
Complete EDTA-free protease inhibitor	Roche	11 873 580 001
^{14}C-S-ADENOSYL-L-METHIONINE	Perkin Elmer	NEC363010UC
N^6-methyl adenosine	Sigma-Aldrich	M2780
Anti-HA Affinity Matrix	Roche	Cat. no. 11815016001; RRID:AB_390914
Critical Commercial Assays		
NEBNext Multiplex Small RNA Library Prep Set for Illumina	NEB	E7300
MinElute Gel Extraction Kit	QIAGEN	28604
MEGAshortscript T7 Transcription Kit	Life technologies	Cat. no. AM1354
Dynabeads Protein A	Life Technologies	10002D
Deposited Data		
Deep sequencing datasets	This study	GEO accession: GSE116329
All raw gel data are deposited at Mendeley Data.	This study	https://doi.org/10.17632/ny82j2ngt5.1
Structure: METTL16-core, crystal form 1	This study	PDB ID: 6GFN
Diffraction images: METTL16-core, form 1	This study	DOI:10.15785/SBGRID/578
Structure: METTL16-core, crystal form 2	This study	PDB ID: 6GT5
Diffraction images: METTL16-core, form 2	This study	DOI:10.15785/SBGRID/579
Structure: METTL16-DN	This study	PDB ID: 6GFK
Diffraction images: METTL16-DN	This study	DOI:10.15785/SBGRID/577
Experimental Models: Cell Lines		
Sf21 insect cells for protein production	Eukaryotic Expression Facility, EMBL Grenoble, France	N/A
High Five insect cells for protein production	Eukaryotic Expression Facility, EMBL Grenoble, France	N/A
Experimental Models: Organisms/Strains		
Mouse: *Mettl16 knock-out*	This study	Available from Lead Contact
Oligonucleotides		
DNA and RNA oligos		See Table S1
Recombinant DNA		
pACEBac2	Bieniossek et al., 2012	N/A
Human *Mettl16* cDNA	This study	NP_076991; NM_024086

(*Continued on next page*)

Continued

REAGENT or RESOURCE	SOURCE	IDENTIFIER
Software and Algorithms		
Cutadapt		http://journal.embnet.org/index.php/embnetjournal/article/view/200
ENRICHR	Chen et al., 2013; Kuleshov et al., 2016	http://amp.pharm.mssm.edu/Enrichr/
MEME - Motif discovery tool	Bailey and Elkan, 1994	
R	R Core Team, 2017	https://www.r-project.org
DESeq2	Love et al., 2014	N/A
Bioconductor	Huber et al., 2015	https://www.bioconductor.org/
Gviz	Hahne and Ivanek, 2016	N/A
STAR	Dobin et al., 2013	N/A
Salmon	Patro et al., 2017	N/A
tximport	Soneson et al., 2015	N/A
featureCounts	Liao et al., 2014	N/A
JunctionSeq	Hartley and Mullikin, 2016	N/A
RNAfold	Lorenz et al., 2011	N/A
Phaser	McCoy et al., 2007	http://www.phaser.cimr.cam.ac.uk/index.php/Phaser_Crystallographic_Software
XDS suite	Kabsch, 2010	http://xds.mpimf-heidelberg.mpg.de
autoPROC	Vonrhein et al., 2011	http://www.globalphasing.com/autoproc/
STARANISO	Tickle et al., 2017	http://staraniso.globalphasing.org/cgi-bin/staraniso.cgi
Coot	Emsley et al., 2010	http://www2.mrc-lmb.cam.ac.uk/personal/pemsley/coot
BUSTER	Bricogne et al., 2016	http://www.globalphasing.com/buster
MOLPROBITY	Chen et al., 2010	http://molprobity.biochem.duke.edu
PyMOL	Molecular Graphics System, Version 1.8.6 Schrodinger, LLC	https://pymol.org/2/
PDB2PQR	Dolinsky et al., 2004	http://nbcr-222.ucsd.edu/pdb2pqr_2.0.0/
SBgrid	Morin et al., 2013	https://sbgrid.org/
Other		
Chelating Sepharose Fast Flow beads	GE Healthcare	17-0575-01
StrepTrap HP	GE Healthcare	28-9075-46
Superdex S75 10/300 GL	GE Healthcare	17-5174-01
Superdex 200 10/300 GL	GE Healthcare	17-5175-01
MethaPhor agarose	Lonza	50180

CONTACT FOR REAGENT AND RESOURCE SHARING

Further information and requests for resources and reagents should be directed to and will be fulfilled by the Lead Contact, Ramesh S. Pillai (ramesh.pillai@unige.ch).

EXPERIMENTAL MODEL AND SUBJECT DETAILS

Animal Work

Mutant mice were generated at the Transgenic Mouse Facility of University of Geneva. The mice were bred in the Animal Facility of Sciences III, University of Geneva. The use of animals in research at the University of Geneva is regulated by the Animal Welfare Federal Law (LPA 2005), the Animal Welfare Ordinance (OPAn 2008) and the Animal Experimentation Ordinance (OEXA 2010). The Swiss legislation respects the Directive 2010/63/EU of the European Union. Any project involving animals has to be approved by the Direction Générale de la Santé and the official ethics committee of the Canton of Geneva, performing a harm-benefit analysis of the project. Animals are treated with respect based on the 3Rs principle in the animal care facility of the University of Geneva. We use the lowest number of animals needed to conduct our specific research project. Discomfort, distress, pain and injury is limited to what is

indispensable and anesthesia and analgesia is provided when necessary. Daily care and maintenance are ensured by fully trained and certified staff. This particular work was approved by the Canton of Geneva (GE/6/18).

Mettl16 knockout mice

The *Mettl16* gene locus is located on mouse chromosome 11 and consists of 10 exons (Figure S4A). We targeted the endogenous *Mettl16* locus in mouse embryos of the B6D2F1/J hybrid line (also called B6D2; The Jackson Laboratory, stock no. 100006). It is a cross between C57BL/6J (B6) and DBA/2J (D2), and heterozygous for all B6 and D2 alleles. Single-cell mouse embryos were injected with a guide RNA (gRNA) that directs the DNA endonuclease Cas9, and a 170 nt single-stranded DNA (ssDNA) repair template (IDT). The ssDNA carries a triple-stop codon flanked by a 81 nt 5′ homology arm and a 75 nt 3′ homology arm. Founder mice were identified by genotyping PCR (Figure S4B) and crossed with wild-type C57BL/6JRj (Janvier) partners to obtain germline transmission. We obtained two lines: line #2112 where the homology recombination template was inserted, resulting in a triple-stop codon cassette (sequence: ATGTAAATAGATGA) in exon 3, and line #2175 where a 7 bp deletion led to removal of a splicing donor site in intron 4. It is expected that creation of premature termination codons in both lines should result in removal of the transcripts via nonsense-mediated decay (NMD). Heterozygous *Mettl16*$^{+/-}$ mice of both sexes were viable and fertile, while homozygous mutants were not recovered in born litters (Figure S4C). Indeed, our analysis indicates that homozygous *Mettl16*$^{-/-}$ mutation results in embryonic lethality around implantation (Figures 4, 5, and S4D–S4F). Both the generated lines showed the embryonic lethality phenotype. We used the line #2112 (with the triple-stop codon cassette) for sequence analysis of early embryos.

Preparation of gRNAs: A cloning-free method was used to prepare DNA template for *in vitro* transcription of the chimeric crRNA-tracrRNA, termed single guide RNA (sgRNA or gRNA). Briefly, a common reverse primer (CRISPR sgR primer) and a gene specific forward primer (CRISPR F primer) with T7 promoter sequence was used to PCR amplify the single-stranded sgDNA template. Primer sequences are provided in Table S1.

Forward (F) primer design template:

5′-GAAATTAATACGACTCACTATAGGNNNNNNNNNNNNNNNNNNNNGTTTTAGAGCTAGAAATAGC-3′

N represent the gene-specific sequence.

The following components were mixed to prepare the PCR reaction: 20 μl 5X Phusion HF buffer, 67 μl ddH2O, 2 μl 10 mM dNTPs, 5 μl of 10 μM CRISPR F primer, 5 μl of 10 μM CRISPR sgR primer, and 1 μl Phusion DNA polymerase. The PCR reaction was set as follows: 98°C for 30 s, 35 cycles of [98°C for 10 s, 60°C for 30 s and 72°C for 15 s], 72°C for 10 min, and finally at 4°C to hold the reaction. The PCR product (~110bp) was agarose gel-purified using mini-elute gel extraction kit (QIAGEN, cat. no. 28604). The purified DNA was used to produce gRNA by *in vitro* transcription via the T7 promoter. *In vitro* transcription was carried out with the MEGAshortscript T7 Transcription Kit (Life technologies; cat no. AM1354) for 4 hours at 37°C. Reactions were treated with DNase I to remove template DNA, phenol-chloroform extracted and precipitated with ethanol. Quality of the generated gRNA was verified by 1.2% agarose gel electrophoresis.

Denaturing formaldehyde-agarose gel electrophoresis: Quality of generated gRNAs were verified by 1.2% agarose-formaldehyde gel electrophoresis. Agarose gel was prepared by mixing 0.6 g agarose, 36.5 mL H_2O, 5 mL of 10x MOPS buffer (0.2 M MOPS, 80 mM sodium acetate, 10 mM EDTA) and 8.5 mL of 37% formaldehyde. Approximately, 4 μg of RNA was dissolved in the 4xRNA loading buffer (50% formamide, 6.5% formaldehyde, MOPS buffer 1x, bromophenol blue 0.2%, ethidium bromide 50 μg/ml) and heated to 65°C for 10 min. RNA was loaded into the gel and run at 70V for approximately 90 minutes. Gel was imaged in the E-Box VX5 (Vilber Lourmat, France) imaging station.

Preparation of injection mix: We mixed 12.5 ng/μl of the gRNA with 12.5 ng/μl of the 170 nt ssDNA repair template (IDT), and 25 ng/μl of Cas9 mRNA (ThermoFischer Scientifique; A29378), in injection buffer (10 mM Tris pH 7.5, 1 mM EDTA, pH 8.0). Prepare aliquots of 20 μL and store at −80°C.

Sequence of ssDNA repair template used: The triple-stop codon sequence is highlighted (bold, italic).

ssDNA (negative-strand sequence)

AGTTGAGAATGCAAAACCTATGGAAGTAAGAACCACCTACCTATGTCAATTCCTCTTCGGAGAGTAGTTTTGTCCGAATCC***TCATCTATTTACAT***TGGTGACCAATCAAATCTTCTACCCAGTGAATATAGTTAAGTCTCAAGGGGACTGTGGGAATTAGTCTCTCCAAA

Injection of mouse embryos of the hybrid background B6D2F1/J (black coat color) was carried out at the Transgenic Mouse Core Facility, University Medical Centre (CMU), University of Geneva. The B6D2F1/J hybrid line (also called B6D2; The Jackson Laboratory, stock no. 100006) is a cross between C57BL/6J (B6) and DBA/2J (D2), and heterozygous for all B6 and D2 alleles. The NMRI (Naval Medical Research Institute) mice, which have a white coat color were used as foster mothers.

Genotyping

Ear punches of the weaned animals (21 days-old) were digested in 100 μl of buffer containing 10 mM NaOH, 0.1 mM EDTA for 120 min at 95°C. After centrifugation at 3000 rpm for 10 min, 50 μl of supernatant was transferred to a new tube containing 50 μl of TE buffer (20mM Tris-HCl, pH 8.0 and 0.1 mM EDTA). An aliquot of 2 μl of the digestion mix was used for PCR.

Primers to detect bands (Figure S4B) corresponding to the wild-type (344 bp, WT), the triple-stop codon knock-in (358 bp, 2112) and 7 bp deletion (337 bp, 2175) alleles were MMoligo109 and MMoligo110 (Table S1). Identity of the bands were confirmed by Sanger sequencing.

Reaction mix for 25 μl PCR reactions: 1 × Taq buffer (without $MgCl_2$, ThermoFisher cat. no. B38), 2 mM $MgCl_2$, 0.5 μl dNTPs mix (stock 10 mM), 0.5 μl primer mix (stock 10 nM each), 2.0 μl tail DNA (100-200 ng), 0.5 μl Taq Pol (EMBL Protein Expression Facility, Heidelberg), water to make 25 μl final volume. Reactions were run using the following conditions (94°C, 20 s; 60°C, 30 s; 72°C, 30 s) for 35 cycles. Reactions were examined by 2.5% agarose gel electrophoresis (Figure S4B).

Mouse embryos

Heterozygous *Mettl16*$^{+/-}$ adult (8 weeks-old) females were superovulated by hormone injections for E2.5 and E3.5 embryo collections. Briefly, one intraperitoneal (IP) injection of five International Units (IU) per mouse (volume, 0.1ml) of pregnant mare serum gonadotropin (PMSG; Folligon, MSD Animal Health) was given two days before crossing with males (at day −2). A second IP injection of 5 IU/mouse (volume, 0.1ml) of human chorionic gonadotropin (HCG; Chorulon, MSD Animal Health) at day 0 was administered to the females. The females were mated with *Mettl16*$^{+/-}$ males immediately after the injections and checked for plugs the day-after (E0.5). The females were sacrificed 2 or 3 days later (embryonic days E2.5 or E3.5) to collect embryos at 16-cell morula and ≤ 64-cell blastocyst stages, respectively.

For single-embryo transcriptome sequencing, the isolated E2.5 and E3.5 embryos were visually examined for viability and cell number, and transferred separately into single tubes of 0.2 mL thin-walled 8-tube PCR strips (Thermo, AB-0451). The tubes contained 2 μL of the following mix: 0.4% Triton X-100 (vol/vol) in H2O + 2U/μl SUPERase• In RNase Inhibitor (20 U/μL; Thermo, AM2694). Embryos were stored at −80°C prior to processing for Smart-seq2 library preparation (Picelli et al., 2014).

For genotyping E2.5, E3.5 embryos, these were collected as above from superovulated heterozygous *Mettl16*$^{+/-}$ females and placed individually into single tubes of 0.2 mL thin-walled 8-tube PCR strips with 10 μl of lysis buffer [GoTaq G2 DNA Polymerase buffer (Promega, M7841), 200 μg/ml Proteinase K]. Embryos were lysed for 1h at 55°C, and then Proteinase K was inactivated by heating to 96°C for 10 min. 5 μl of the mix was used for PCR. Reaction mix for 20 μl: 5 x GoTaq G2 DNA Polymerase Buffer, 200 μM dNTP mix, 250 μM primers, 0.25 μl GoTaq G2 DNA Polymerase, 5 μl DNA. Reactions for oligo pair MMoligo109 + MMoligo110 were run using the following conditions (94°C, 20 s; 60°C, 30 s; 72°C, 30 s) for 35 cycles. Reactions were examined by 2% agarose gel electrophoresis.

For genotyping E6.5, E8.5 and E12.5 embryos, these were collected from heterozygous *Mettl16*$^{+/-}$ females without superovulation. *Mettl16*$^{+/-}$ females were mated with *Mettl16*$^{+/-}$ males and plugs were checked on the day-after (E0.5). Plugged animals were separated. The females were sacrificed 6, 8 or 12 days later, in the late afternoon (between 4 pm to 7 pm). After dissection, embryos were placed in 50 μl of RNA*later* Stabilization Solution (ThermoFisher, AM7020) and kept at −80°C until isolation. RNA and DNA were extracted simultaneously using DNeasy Blood and Tissue Kit (QIAGEN, 69504) and RNeasy Plus Micro Kit (QIAGEN, 74034). RNA was stored at −80°C. 2 μl of DNA was used for genotyping. Reaction mix for 20 μl: 5 x GoTaq G2 DNA Polymerase Buffer, 200 μM dNTP mix, 250 μM primers, 0.25 μl GoTaq G2 DNA Polymerase, 2 μl DNA. Reactions for oligo pair MMoligo109 + MMoligo110 were run using the following conditions (94°C, 20 s; 60°C, 30 s; 72°C, 30 s) for 35 cycles. Reactions were examined by 2% agarose gel electrophoresis. For embryos for which agarose gel electrophoresis was not conclusive, the PCR was repeated and reaction products were cloned into pCR 2.1 vector using The Original TA Cloning Kit (ThermoFisher, 45-0046). Positive clones were selected and sequenced by Sanger sequencing.

METHOD DETAILS

Clones and constructs

Constructs for mammalian cell expression

Coding sequence for full-length (FL) human METTL16 (hMETTL16; 562 aa; Accession number NP_076991) was amplified from human HeLa cell total RNA by reverse transcription-PCR (RT-PCR). A mammalian expression vector (pCI-neo vector backbone) allowing production of 3xFLAG-HA tagged proteins from a cytomegalovirus (CMV) promoter was used. Sequence of the tag: ATG GACTACAAAGACCATGACGGTGATTATAAAGATCATGATATCGATTACAAGGATGACGATGACAAGggcggcagcggcTACCCATATG ATGTTCCAGATTACGCT.

Constructs for insect cell expression

For the production of FL proteins, we used Baculovirus-mediated expression in insect cells. The full-length (1-562 aa) human METTL16 (hMETTL16) was cloned into the pACEBac2-SUMO acceptor vector (Bieniossek et al., 2012) for expression as an N-terminal 6xHis-Strep-SUMO-TEV fusion in the insect cells. For co-expression of human METTL3 and METTL4, full-length coding sequence for human METTL3 (1-580 aa) was cloned into the NheI and SphI restriction sites of the modified acceptor vector pACEBac2 to express the recombinant proteins with N-terminal 6xHis-SUMO-StrepIII-TEV fusions. The full-length coding sequence for untagged hMETTL14 (1-456) was cloned into the donor vector pIDK between KpnI/XhoI restriction sites. The proteins were co-expressed by taking advantage of MultiBac system (Bieniossek et al., 2012) which allows the generation of multi-gene constructs via Cre-lox recombination. The acceptor and donor vectors were combined in Cre-mediated reaction in total volume of 20 μl where 2 μg of each vector was mixed with 2 μl of 10x Cre buffer and 1 μl of Cre recombinase (NEB, cat no. M0298S). The reaction was incubated at 37°C for 1 h. After that, 5 μl of Cre reaction was transformed to 100 μl of competent TOP10 cells and plated on LB agar with appropriate antibiotics. The clones were verified by restriction digestion of the isolated plasmid, as well as by PCR.

Constructs for bacterial expression

Constructs covering only the core methyltransferase domain of hMETTL16 (1-291 aa) or its point mutant/deletion versions were cloned into the bacterial expression vector (pETM-11-SUMO vector; EMBL Protein Expression and Purification Core Facility) as 6xHis-Strep-SUMO-TEV fusions. The following constructs were prepared:

METTL16-ΔN: 40-291 aa, N-terminal deletion version similar to that used in PDB ID: 2H00 [Structural Genomics Consortium (SGC)].

METTL16-core: 1-291 aa.

METTL16-core mutants

1. Single amino acid changes: K5A, R10A, R12A, K14A, K16A, K5E, K10E, K10D, K47E, R74E, R82E, F187G, R279E, R282E.
2. MUT1: five residues (K5, R10, R12, K14, and K16) mutated to As.
3. MUT2: two residues (K26 and K31) mutated to As.
4. MUT3: combination of MUT1 and MUT2 sites mutated to As.
5. PP185-186AA: two residues (P185 and P186) mutated to As.
6. Loop-4P-A: four residues (P202, P205, P206, P207) mutated to As.
7. Loop-3R-E: three residues (R200, R203, R204) mutated to Es.
8. Loop-del: deletion of disordered loop 190-218 aa and replaced with a linker GGGSGGGS.
9. Double mutations in the binding groove: two residues (K47 and R279) mutated to Es.

Antibodies

The polyclonal rabbit anti-m^6A (Synaptic Systems; 202003), antibody for detecting mouse METTL16- polyclonal rabbit anti-METT10D (abcam, ab186012) and normal mouse IgG (Santa Cruz, sc-2025) antibodies were purchased. Anti-HA affinity matrix (Roche; cat. no. 11815016001) and Pierce HA Epitope Tag Antibody (ThermoFisher, cat.no. #26181) were used for immunoprecipitations.

Recombinant protein production

Production of full-length recombinant proteins was carried out in insect cell lines using the baculovirus expression system. The ovary-derived cell lines used are: High Five (Hi5) insect cell line originating from the cabbage looper (*Trichoplusia ni*) and the Sf9 cells derived from the fall army worm *Spodoptera frugiperda*. Briefly, recombinant full-length hMETTL16 coding sequence was cloned into pACEBac2-Sumo acceptor vector (His-Strep-Sumo tag) (Bieniossek et al., 2012). Plasmids were transformed into DH10EMBacY competent cells for recombination with the baculovirus genomic DNA (bacmid). The bacmid DNA was extracted and transfected with FuGENE HD (Promega, cat. no. E231A) into the *Sf*9 insect cells for virus production. The supernatant (V_0) containing the recombinant baculovirus was collected after 72 to 96 hours post-transfection. To expand the virus pool, 6.0 mL of the V_0 virus stock was added into 25 mL of *Sf*9 (0.5×10^6/mL) cells. The resulting cell culture supernatant (V_1) was collected 24 h post-proliferation arrest. For large-scale expression of the protein, Hi5 cells were infected with virus (V_1) and cells were harvested 72 h post-proliferation arrest.

For bacterial expression, plasmids were transformed into the *E. coli* BL21(DE3) strain and expression was initiated by addition of 0.7 mM Isopropyl β-D-1-thiogalactopyranoside (IPTG) when the culture density reached 0.6 (OD_{600}). The proteins were then expressed overnight at 20°C following induction.

Purification of METTL3-METTL14 complex

Insect cells co-expressing hMETTL3 and hMETTL14 were resuspended in the lysis buffer (50 mM Tris-HCl pH 8.0, 300 mM NaCl, 40 mM Imidazole, 5% glycerol, 0.1% Triton X-100, 5 mM 2-mercaptoethanol, proteinase inhibitor (Roche, Complete EDTA-free) and Benzonase (Millipore), sonicated with MISONIX Sonicator S-4000 and the lysate was centrifuged at 20,000 rpm for 30 min at 4°C. The clarified supernatant was incubated at 4°C for 2h with the Ni^{2+} chelating Sepharose FF beads (GE Health; cat. no. 17057501). The beads were washed with buffer W300 (50 mM Tris-HCl pH 8.0, 500 mM NaCl, 50 mM Imidazole, 0.1% Triton X-100, 5 mM 2-mercaptoethanol) and W500 (50 mM Tris-HCl pH 8.0, 500 mM NaCl, 40 mM Imidazole, 0.2% Triton X-100, 5 mM 2-mercaptoethanol). Finally, His-tag proteins bound to the beads were eluted with the elution buffer (50 mM Tris-HCl pH 8.0, 300 mM NaCl, 300 mM Imidazole, 0.1% Triton X-100, 5 mM 2-mercaptoethanol). The tag (His-Strep-Sumo) was cleaved overnight with TEV in the dialysis buffer (50 mM Tris-HCl pH 8.0, 250 mM NaCl, 5 mM 2-mercaptoethanol). After cleavage, second Ni-column purification was performed and supernatant containing the cleaved protein was collected. Proteins were further purified over the ion exchange column (HiTrap™ Q Sepharose HP, 1ml, GE healthcare, cat. no. 17-1153-01). Fractions containing the recombinant proteins were further purified by gel filtration chromatography using Superdex S200 10/300GL equilibrated with gel-filtration buffer containing: 50 mM Tris-HCl pH 8.0, 200 mM NaCl, 5 mM 2-mercaptoethanol (GE Healthcare, cat. no. 17-5175-01). The fractions eluting at 11 mL of elution volume were checked by SDS-PAGE analysis (Figure S1C) and pure hMETTL3-hMETTL14 protein complexes were concentrated and flash frozen in liquid nitrogen after addition of 10% glycerol.

Purification of METTL16

The insect cells or bacterial cells were collected by centrifugation and lysed by sonication [25 mM Tris-HCl, pH 8.0, 400 mM NaCl, 5% Glycerol, 0.5% Tween-20, 5 mM 2-mercaptoethanol, 20 mM Imidazole and protease inhibitor (Roche complete EDTA-free)]. After incubation for two hours with Ni-NTA beads, the fusion protein was eluted with Imidazole (250 mM), and the His-SUMO tag was

cut by the TEV protease (10 μg of protease per 1 mg of fusion protein; EMBL Protein expression and purification facility). The cleaved tag was removed by a second purification on Ni-NTA beads. The protein was further purified by gel filtration chromatography (Superdex S75 or Superdex 200, GE Healthcare) in the buffer (25 mM HEPES, pH 7.2, 150 mM NaCl, 2 mM DTT). The elution volumes of both full-length METTL16 and METTL16-core and METTL16-ΔN during gel-filtration chromatography are consistent with the proteins being a monomer (Figure S1B). The pure fractions were verified by SDS-PAGE (Figure S1E), and used for crystallization and biochemical assays. One of the METTL16-core mutants (Loop-3R-E) showed aberrant migration in the denaturing gel, but its identity was confirmed by mass spectrometry and shows normal elution profiles during gel-filtration chromatography (Figure S2F).

Limited proteolysis of hMETTL16-FL

For limited proteolysis, we used a 1:1000 ratio of protease:protein (if the protease is freshly prepared, use 1:500 ratio). Take 100 μL of METTL16-FL (concentration 1 μg/μl) protein solution and mix with 2 μL the protease Trypsin (concentration is 50 ng/μl). This makes a total of 102 μL reaction mix. Incubate at 25°C and remove aliquots of 25 μL at time-points 0, 5, 30 and 60 minutes. Aliquots are immediately mixed with gel loading dye, boiled at 95°C, and stored at −20°C. Reactions are then resolved via SDS-PAGE (Figure S1E). Peptide boundaries of proteolysis fragments were identified by mass spectrometry at the Proteomics Core Facility, EMBL, Heidelberg.

Crystallization and data collection

Optimal crystallization conditions for full-length human METTL16 (1-562 aa) and the human METTL16-core (1-291 aa) proteins were sought by robot screening at the High Throughput Crystallization Facility at EMBL Grenoble, France. Only the METTL16-core gave crystals in this screen. Once conditions were identified, crystals were manually produced: 2 μL protein solution at 13 mg/ml was manually mixed with 2 μL reservoir solution using the hanging drop method at room temperature. The reservoir conditions used were either 0.2 M di-sodium tartrate, 20% (w/v) PEG 3350 or 0.1 M Bis-Tris propane, pH 6.5, 0.2 M potassium-sodium tartrate, 20% (w/v) PEG 3350. We additionally crystallized the human METTL16-ΔN (40-291 aa) version using conditions previously described in PDB ID: 2H00 [Structural Genomics Consortium (SGC)]. The crystals were then flash-frozen at 100K after transferring them to identical crystallization conditions containing 20% glycerol. Diffraction data were collected on ID23-2 (Flot et al., 2010) and ID30B (McCarthy et al., 2018) at the European Synchrotron Radiation Facility (Grenoble, France), and integrated using the XDS suite (Kabsch, 2010). The diffraction data from hMETTL16-core (1-291 aa) crystals were highly anisotropic, with diffraction limits of ~2.8 Å and 2.4 Å along the best direction for crystal form 1 and 2 respectively, but only ~3.6 Å in the weakly diffracting directions. Therefore, data were processed using STARANISO (Tickle et al., 2017), as implemented in autoPROC (Vonrhein et al., 2011), which applies non-elliptical anisotropic limits based on a locally averaged mean $I/\sigma(I)$ cut-off, performs a Bayesian estimation of structure amplitudes, and applies an anisotropic correction to the data. Detailed crystallographic statistics are provided in Table 1.

Structure determination and refinement

The hMETTL16-core (1-291 aa) structure was solved by molecular replacement using the METTL16-ΔN, N-terminal deletion structure (PDB ID: 2h00) as a search model with Phaser (McCoy et al., 2007). Several rounds of manual building with Coot (Emsley et al., 2010), and structure refinement with BUSTER (Bricogne et al., 2016) were carried out for all structures. MOLPROBITY (Chen et al., 2010) was used for model validation and all the crystallographic information is summarized in Table 1. The atomic coordinates and structure factors have been deposited in the Protein Data Bank with the accession codes: 6GFN (METTL16-core, crystal form 1), 6GT5 (METTL16-core, crystal form 2) and 6GFK (METTL16-ΔN). For modeling a bound RNA into the METTL16-core structure we used a tRNA from PDB ID: 2ZZM (Goto-Ito et al., 2009). Structural figures were prepared with PyMOL (Schrödinger, LLC). The electrostatic potential was calculated using PDB2PQR (Dolinsky et al., 2004) and displayed in PyMOL using the APBS plugin. For modeling of adenosine into the METTL16-core structure we used a 20-mer DNA from the complex structure of the MTase EcoP15I (PDB: 4ZCF, chain B)(Gupta et al., 2015).

While this study was in preparation, Ruszkowska et al. reported the crystal structure (PDB ID: 6B92) of METTL16 core MTase domain (Ruszkowska et al., 2018). A comparison with our structure (PBD ID: 6GFN) reveals a very high degree of overlap (rmsd = ~0.38 Å for superimposition of 187 C_α atoms) (Figure S1F). Nevertheless, there are some differences. First, the N terminus in our structure is longer by four amino acids. Second, there are differences in labile loops between α4-β3 (96-99 disordered in ours); α7-β6 (poor density in ours); and β6-α8 (the long catalytic loop). The catalytic NPPF residues (in both our METTL16-core structures, Table 1) are more similar to their apo form (PDB ID: 6B91) than their SAH-bound form (PDB ID: 6B92) (Ruszkowska et al., 2018). They also have eight additional residues on α8, but these are not helical as in our METTL16-ΔN structure (PBDB ID: 6GFK) (Figure 1D).

In vitro transcription of RNA substrates for methylation assay

Templates for *in vitro* transcription (of full-length human *MAT2A* mRNA hairpin 1 and human U6 snRNA RNA) (Pendleton et al., 2017) were amplified in a PCR reaction to prepare a single-stranded DNA template with T7 promoter sequence. The T7 promoter sequence 5′- TAATACGACTCACTATAGGG −3′ was introduced at the 5′ end of forward primer followed by a specific sequence. The reverse primer had a 20 nt overlap with the forward primer allowing for efficient base pairing. The primers used for template preparation are given in Table S1. The following components were mixed to prepare the PCR reaction: 20 μl 5X Phusion HF buffer, 67 μl ddH2O, 2 μl 10 mM dNTPs, 5 μl of 10 μM Forward primer, 5 μl of 10 μM Reverse primer, and 1 μl Phusion DNA polymerase. The PCR reaction

Table 1. Data Collection and Refinement Statistics

Protein PDB Code	ΔN MTase 6GFK	MTase (form 1, SAH) 6GFN	MTase (form 2, apo) 6GT5
Wavelength (Å)	0.9763	0.8731	0.8731
Resolution range (Å)	46–2.3 (2.38–2.3)	82–2.86 (3.2–2.86)	80–2.5 (2.8–2.5)
Space group	$P3_121$	$I4_122$	$P4_12_12$
Unit cell (Å)	133.8, 133.8, 78.7<br>90, 90, 120	93.4, 93.4, 180.7<br>90, 90, 90	89.6, 89.6, 179.1<br>90, 90, 90
Unique reflections	36,074 (3,538)	5,724 (286)	12,391 (619)
Completeness (%)			
Spherical	99.4 (99.6)	60.9 (10.5)	45.9 (6.8)
Ellipsoidal	N/A	93.1 (78.7)	93.3 (79.9)
Mean < I/σI >	10.3 (1.4)	8.0 (1.8)	4.5 (1.6)
R_{pim} (%)	3.7 (55.0)	7.3 (50.3)	,12.4 (50.2)
CC*	0.994 (0.996)	0.996 (0.59)	0.967 (0.647)
R_{work} (%)	19.4 (21.2)	18.1 (22.1)	18.2 (24.0)
R_{free} (%)	22.8 (23.5)	21.6 (30.5)	23.4 (30.5)
Number of non-H Atoms			
Macromolecules	5,307	2,024	4,011
Water	100	11	25
SAH/ion	103	26	–
R_{msd} (bonds, Å)	0.009	0.01	0.009
R_{msd} (angles, °)	1.06	1.11	1.10
Ramachandran Plot (%)			
Favored	97.3	94.4	93.2
Allowed	2.7	4.4	6.0

Statistics for the highest resolution shell are shown in parentheses.

conditions were set as follows: 98°C for 30 s, 35 cycles of [98°C for 10 s, 60°C for 30 s and 72°C for 15 s], 72°C for 10 min, and finally at 4°C to hold the reaction. The PCR product (~110bp) was agarose gel-purified using mini-elute gel extraction kit (QIAGEN, cat. no. 28604). The purified DNA was used to produce RNA by *in vitro* transcription reaction via the T7 promoter. *In vitro* transcription was carried out with the MEGAshortscript T7 Transcription Kit (Life technologies; cat no. AM1354) for 4 hours at 37°C. Reactions were treated with DNase I to remove template DNA, phenol-chloroform extracted and precipitated with ethanol. *MAT2A* hairpin RNA was 82 nt long, while the U6 snRNA was 83 nt long. Quality of the generated RNA was verified by 1.2% agarose gel electrophoresis.

In vitro RNA methylation assay with METTL16

Some methylation assays were carried out with *in vitro* transcribed RNAs (*MAT2A* mRNA hairpin 1 or U6 snRNA), while the majority were with chemically synthesized RNA oligos (Microsynth, Switzerland) (Table S1). Recombinant human METTL16 proteins (FL, core, ΔN and mutant versions) or a heterodimer of human METTL3/METTL14 were used.

Prior to the experiment, the RNAs were refolded by heating 100 μM RNA solution in 10 mM NaCl in a thermoblock to 70°C for 5 min. and slowly cooling down to room temperature, while keeping the tubes in a heat block. All methylation reactions were performed in a 50 mM Tris-HCl, pH 7.5, 100 mM KCl, 5 mM $MgCl_2$, 2 mM DTT buffer with 10 μM of refolded single-stranded RNA, 5 μg of recombinant protein, 1 μl of RiboLock RNase Inhibitor (ThermoFisher, cat. no. EO0381) and 0.1 μCi of ^{14}C-SAM (Perkin Elmer, NEC363010UC) in a total volume of 50 μl. Unless otherwise indicated, all reactions were performed overnight at 37°C. For reactions with RNA oligos designed based on m^6A-IP-RNaseq experiment (Figure 3J), these were performed overnight at 22°C. RNA was subsequently isolated using phenol/chloroform extraction protocol. RNA pellets were resuspended in 2x RNA loading buffer (90% formamide, 0.02% SDS, 1 mM EDTA, 0.02% bromophenol blue, 0.02% xylene cyanol), heated for 5 min. at 70°C, cooled down to the room temperature and resolved in a 15% Urea-PAGE gel.

The 15% Urea-PAGE gel was prepared by mixing 12.6 g of urea, 3 mL of 10x TBE (1 M Tris base, 1 M boric acid, 0.02 M EDTA), 11.25 mL of 40% acrylamide (19:1) and 6.75 mL of H_2O. To catalyze gel polymerization, 240 μl of APS and 24 μl of TEMED were added. Gel was left for 40 min. at room temperature to polymerize. Wells were flushed with 1XTBE to remove urea deposits and gel was pre-run in 1X TBE at 20 W for 25 min to warm the gel. After the pre-run, ssRNA marker labeled with ^{32}P-γ-ATP and composed of four single-stranded RNA oligos (RP_RNA_19: 40 nt, RP_RNA_1: 30 nt, RP_RNA_3: 28 nt, RP_RNA_18: 16 nt; Table S1) was loaded into the gel, together with RNA samples from the *in vitro* methylation assay. Gel was run at 12 W for 1 h 30 min. Then, dried

in a gel dryer (Bio-Rad, model 583) with a gradual heating and cooling program, 80°C for 3 h. Dried gel was exposed to a phosphor screen BAS (GE Healthcare) for 24 h. The phosphor screen was scanned in a Typhoon FLA 9500 laser scanner (GE Healthcare) at 700V and 100 μm pixel size using control software (1.1 version) for Typhoon FLA 9500. Scans were analyzed using ImageQuant TL 8.1 software (GE Healthcare).

The quality of RNAs used for methylation assays were verified by Methylene Blue staining. In some experiments, after the methylation reaction products were resolved by urea-PAGE, the gel was stained with Methylene Blue, imaged to verify integrity of RNAs present in the reaction (Figure S3A) and then dried for exposure to the phosphor storage screen to detect radioactivity signals (Figure 3A).

UV crosslinking assay

Preparation of labeled RNA: RNA6 (100 pmol) was 5′-end labeled with [γ-^{32}P]ATP and T4 Polynucleotide Kinase (NEB, M0201) for 1 h at 37°C. Labeled RNA was resolved on 15% Urea-PAGE gel and exposed with phosphor screen BAS (GE Healthcare) for 5 minutes. RNA band corresponding to the size of 29 nt was cut from the gel. The RNA was eluted from gel by overnight incubation in 300 mM NaCl at room temperature and with shaking (750 rpm). RNA was extracted by phenol-chloroform and resuspended in 20 μl of H_2O. See Table S1 for RNA sequence.

METTL16 proteins (final concentration 0.4 μM and 2 μM) were mixed with 1 μl of labeled RNA6 in RNA binding buffer (10 mM Tris-HCl, pH 8.0, 50 mM NaCl, 1 mM DTT, 1 mM SAH) in a final volume of 20 μl and incubated for 2 h on ice. After incubation, reaction mix was deposited inside the cap of the Eppendorf tube, and placed on ice such that the cap touches the ice. Tubes were placed around 4 cm from UV lamp (254 nm) and irradiated for 5 min. (UV Stratalinker 2400, Stratagene). After UV irradiation, samples were boiled for 5 min in SDS loading buffer and resolved by 12% SDS-polyacrylamide gel electrophoresis.

Gel was dried in a gel dryer (Bio-Rad, model 583) with a gradual heating and cooling program, 80°C for 3 h. Dried gel was transferred to the cassette and exposed with a phosphor screen BAS (GE Healthcare) for 24 h. After exposure, phosphor screen was scanned in a Typhoon FLA 9500 laser scanner (GE Healthcare) at 700V and 50 μm pixel size. Control software for Typhoon FLA 9500 was at 1.1 version. Scans were analyzed using ImageQuant TL 8.1 software (GE Healthcare).

Cell culture and transfections

Human embryonic kidney cell line 293 (HEK293) transformed with the SV40 large T antigen (HEK293T) were grown in Dulbecco's modified Eagle Medium (DMEM; Invitrogen, cat. No. 21969-035) supplemented with 10% fetal bovine serum (ThermoFisher; cat. no. 10270106), 1% Penicilline/Streptomycin (ThermoFisher; cat. No. 15140122), 1% 200 mM Glutamine (ThermoFisher; cat. no. 15140122), later referred to as DMEM complete medium (DMEM CM), and maintained in an environment with 5% CO_2 at 37°C. For transfection, cells growing in a 75 cm^2 flask were washed with warm (37°C) 1X PBS and incubated with 1 mL of Trypsin-EDTA 0.05% (ThermoFisher; cat. no. 25300-054) for 1-2 min to promote removal of cells from the growth surface. Subsequently, 10 mL warm DMEM media was added and cells were resuspended by pipetting. Cells were counted using Bürker-Türk and appropriate cell numbers were seeded in cell culture vessels.

Approximately, 4 mL of HEK293T cells were seeded in the 10 cm dish (Falcon, cat. no. 353003) and cultured as described above. When 40 – 50% confluence was reached, cells were transfected with FLAG-HA-METTL16 plasmid: 10 μg of plasmids was diluted in 500 μL of 150 mM NaCl. Simultaneously, 26 μg of linear polyethylenimine, MW 25000 (PEI, Polysciences Inc., cat. no. 23966) was diluted in 500 μL of 150 mM NaCl. Solutions were mixed together, vortexed vigorously for 15 s and incubated for 15 min. at room temperature. Then mix was added to the HEK293T cells in the DMEM CM. After 24 h, medium was changed for the fresh DMEM CM. Cells were grown for 72h in total.

Isolation of human METTL16 complexes for mass spectrometry

Cells in 10 cm dishes were washed 3x with ice cold PBS and 1 mL of lysis buffer [20 mM Tris pH 7.4, 150 mM NaCl, 0.5% Triton X-100, 0.1% sodium deoxycholate, 1 mM EDTA, 0.5 mM DTT, protease inhibitor (Complete Protease Inhibitor Cocktail Tablet, Roche, cat. no. 5056489001)] was added to the cells. Cells were removed from their growth surface using a cell scraper (Costar; cat. no. 3010) and transferred to 1.5 mL eppendorf tubes. Cell lysate was passed 5-times through a 26 G needle (B. Braun Medical Inc., #466-5457) and kept on ice for 15 min. The total cell lysate was spun at 12,000 x g for 10 min at 4°C. After centrifugation, supernatant was transferred to a fresh tube and spun again (12,000 x g, 10 min., 4°C). The cleared lysate was transferred to a fresh tube. While 50 μl of lysate was transferred to a fresh tube and flash-frozen in liquid nitrogen to use as an input, 950 μl was incubated for 4 h at 4°C with 20 μL of Anti-HA Affinity Matrix (Roche, cat. no. 11815016001). After, beads were collected by gentle centrifugation (500 x g for 1 min at 4°C) and eluate was discarded. Beads were washed 5 times with wash buffer (50 mM Tris pH 7.4, 150 mM NaCl, 0.1% Triton X-100, 1 mM EDTA) Then, beads were transferred to fresh 1.5 mL eppendorf tubes and 40 μL of 2x Laemmli buffer (4% SDS, 20% glycerol, 120 mM Tris-HCl pH 6.8, 10% β-mercaptoethanol, 0.02% bromophenol blue) was added. Beads were boiled at 95°C for 5 minutes and stored at −20°C. Proteins were identified by mass spectrometry at the Functional Genomics Center Zurich (ETH Zurich) (Figure S5F). Database searches were performed using the Mascot (SwissProt, human) search program. Applied settings: 1% protein false detection rate (FDR), min. 2 peptides per protein, 0.1% peptide FDR.

Isolation of METTL16 complexes from mouse testes and spleen

An aliquot of 80 μl of Dynabeads Protein A slurry (ThermoFisher, 10001D) was transferred to a fresh Eppendorf tube and washed three times with 1 mL of 20 mM sodium phosphate with 0.02% Tween20. Then, 20 μg of METTL16 antibody (abcam, ab186012) or 20 μg of mouse IgG control antibody (Santa Cruz, cat.no. sc-2025) in 500 μl of 20 mM sodium phosphate with 0.02% Tween20 was added to the beads and incubated overnight at 4°C with rotation.

Next day, two adult (P60) mouse testes and one spleen were cut into pieces using scalpel blade and placed into separate 1.5 mL eppendorf tubes. 500 μL of ice cold lysis buffer [20 mM Tris pH 7.4, 150 mM NaCl, 0.5% Triton X-100, 0.5% sodium deoxycholate, 1 mM DTT, 1 mM EDTA and protease inhibitor (Roche) was added to the tubes. Organs were dounced 15-times using a plastic pestle and left on ice for 10 min. Then, tubes were spun at 12000 x g at 4°C for 10 minutes. Supernatant was transferred to a fresh tube and centrifugation was repeated to clarify the lysate further. Supernatants were transferred to a fresh tube and diluted 2x with dilution buffer dilution buffer [20 mM Tris pH 7.4, 150 mM NaCl, 1 mM EDTA, protease inhibitor] to decrease sodium deoxycholate and Triton X-100 concentration to 0.25%. An aliquot of 50 μl of lysate was transferred to a fresh tube and flash-frozen in liquid nitrogen to use as an input, while rest was transferred to antibody-bound Dynabeads prepared above, and incubated at 4°C for 4 h with rotation.

After 4 h, the supernatant was removed and beads were washed with wash buffer (20 mM Tris pH 7.4, 150 mM NaCl, 0.2% Triton X-100, 1 mM EDTA, 0.5 mM DTT). Washing was repeated four more times, after which beads were transferred to the fresh 1.5 mL eppendorf tubes and 40 μL of 2x Laemmli buffer (4% SDS, 20% glycerol, 120 mM Tris-HCl pH 6.8, 10% β-mercaptoethanol, 0.02% bromophenol blue) was added. Beads were boiled at 95°C for 5 minutes and stored at −20°C. Proteins in the samples were identified at the Functional Genomics Center Zurich (ETH Zurich) with the shotgun mass spectrometry analysis (Figure S5G). Database searches were performed using the Mascot (SwissProt, human) search program. Applied settings: 1% protein false detection rate (FDR), min. 1 peptides per protein, 0.1% peptide FDR.

Mass spectrometry

Mass spectrometry to confirm purified recombinant proteins were carried out at the Proteomics Core Facility, EMBL, Heidelberg. Identification of components within an immunoprecipitated complex was carried out at the Functional Genomics Center Zurich (ETH Zurich) using the shotgun mass spectrometry analysis. Database searches were performed using the Mascot (SwissProt, all species) search program. Applied settings if not stated differently are 1% protein false detection rate (FDR), min. 2 peptides per protein, 0.1% peptide FDR.

Preparation of RNA libraries

In vitro *methylation with METTL16 and m^{6}A-IP-RNaseq*

Libraries of randomized 30 nt RNA sequences were chemically synthesized (Microsynth, CH). The sequences had a constant central 9-mer sequence flanked by randomized (represented by N) sequences (MM-RNA-14: N_{11}-UAC**A**GAGAA-N_{10}). The 9-mer sequence originates from the hairpin 1 of the human *MAT2A* mRNA and carries the m^{6}A methylation site for METTL16 (Pendleton et al., 2017). RNA solutions (100 μM) with 50 mM NaCl were denatured at 80°C for 1 min, and refolded by allowing to cool to room-temperature. *In vitro* methylation reactions containing 15 μL (100 μM) of the above RNA library were carried out in 100 mL reactions (50 mM Tris-HCl, pH 7.5, 100 mM KCl, 5 mM $MgCl_2$, 20U of Riboblock RNase inhibitor, 0.64 mM SAM) with 20 μg of hMETTL16. Reactions were carried out in duplicates and incubated at 37°C, overnight. Reactions were then removed and frozen at −20°C prior to further processing.

A small portion (10%) was left aside to be used as input sample, while the remainder was subjected to immunoprecipitation. The m^{6}A immunoprecipitation was performed as described (Ke et al., 2015). Briefly, 100 μL of Dynabeads Protein A (Life Technologies; 10002D) were washed once in PXL buffer (1 × PBS, 0.1% SDS, 0.5% sodium deoxycholate, 0.5% NP-40) followed by pre-treatment with BSA (final concentration 1μg/ μL) in 200 μL PXL buffer for 45 minutes at room-temperature (RT). BSA pre-treated beads was then conjugated with m^{6}A rabbit polyclonal antibody (5 μg; Synaptic Systems, catalog no. 202003) in 200 μL PXL buffer supplemented with 4 μL of RNasin RNase inhibitor (Promega; N2611) for one hour at RT on a rotating wheel. Dynabeads were further washed twice with PXL buffer and finally beads were resuspended in 400 μL of PXL buffer and 5 μL of RNasin. The *in vitro* methylation reaction prepared above was added to the beads and incubated at 4°C for 2 hours on a rotating wheel. After two hours incubation, the beads were washed twice by ice-cold Nelson low-salt buffer (15 mM Tris at pH 7.5, 5 mM EDTA), once by ice-cold Nelson high-salt buffer (15 mM Tris at pH 7.5, 5 mM EDTA, 2.5 mM EGTA, 1% Triton X-100, 1% sodium deoxycholate, 0.1% SDS, 1 M NaCl), once by ice-cold Nelson stringent wash buffer (15 mM Tris at pH 7.5, 5 mM EDTA, 2.5 mM EGTA, 1% Triton X-100, 1% sodium deoxycholate, 0.1% SDS, 120 mM NaCl, 25 mM KCl), and last by ice-cold NT-2 buffer (50 mM Tris at pH 7.4, 150 mM NaCl, 1 mM MgCl2, 0.05% NP-40). Antibody-bound RNAs were eluted by incubating the beads with 0.5 mg/mL *N*^{6}-methyl adenosine (Sigma-Aldrich; M2780) in NT2 buffer for one hour at 4°C. The eluted RNAs were precipitated with ethanol and glycogen and dissolved in RNase-free water.

The input and IP RNAs were first 3′ end dephosphorylated with T4 PNK (NEB; M0201S, 10 U/μL) in the absence of ATP at 37°C for 45 minutes (40 μL reaction: 35.5 μL RNA, 4 μL 10X T4 PNK buffer, 0.5 μL of T4 PNK) followed by phosphorylation of 5′ end (50 μL reaction: 40 μL dephosphoryated RNA, 6.5 μL water, 1 μL RNasin, 0.5 μL 100 mM ATP, 1 μL 10X T4 PNK buffer 1 μL T4 PNK) at 37°C for 45 minutes. RNAs were phenol chloroform-extracted, ethanol precipitated and resupended in 6 μL of RNase-free water. The input RNA fragments and the immunopurified RNAs after the phosphorylation step were directly used for library preparation (barcoded at 3′ end) using NEBNext® Multiplex Small RNA Library Prep Set for Illumina® (NEB; catalog No. E7560S) following

manufacturer's instructions. The synthesized cDNA libraries were resolved on 3% high-resolution MethaPhor agarose (Lonza; cat. No. 50180) gels in 1X TAE buffer at 70 V. Fragments in the size-range of ~150-250 bp were gel-extracted with the use of MinElute Gel Extraction Kit (QIAGEN; cat No. 28604). Multiple libraries with different barcodes (at 3′ end) were mixed in equimolar ratios and sequenced with the HiSeq Illumina Platform (EMBL GeneCore facility, Heidelberg). The maximum sequencing length was 50 nt. The list of sequencing libraries generated are provided in Table S2.

Mouse single-embryo library preparation

Polyadenylated transcripts in single embryos (E2.5 morula or E3.5 blastocysts) were amplified using the Smart-seq2 protocol (Picelli et al., 2014). The protocol generates libraries that lack strand specificity. Multiple libraries with different barcodes (at the 3′ end) were mixed in equimolar ratios and paired-end sequencing reads were obtained with the HiSeq Illumina Platform (EMBL GeneCore facility, Heidelberg). The maximum sequencing length was 80 nt. The list of sequencing libraries generated are provided in Table S2.

QUANTIFICATION AND STATISTICAL ANALYSIS

In vitro methylation with METTL16 and m^{6}A-IP-RNaseq

Reads were sorted into individual libraries based on the barcodes and the 3′ adaptor sequences were removed using cutadapt 1.9.1 (http://journal.embnet.org/index.php/embnetjournal/article/view/200). Only reads of final length of 30 nucleotides with correctly sequenced TACAGAGAA consensus motif at position 12-20 and without any Ns were kept for further analysis using R 3.4.3 (R Core Team, 2017) and Bioconductor (Huber et al., 2015). To search for possible preference of human METTL16 for specific structured RNA features, we analyzed the predicted secondary structures of the sequenced oligos and compare their representation in between the m^{6}A-IP and input libraries. For each sequence we obtained the minimum free energy (MFE) secondary structure using RNAfold (Lorenz et al., 2011). We used DESeq2 1.18.1 bioconductor package (Love et al., 2014) to obtain the lists of structures significantly enriched or depleted in IP (immunoprecipitation) libraries when compared to input libraries (adjusted p value < 0.1). Top enriched structures were plotted in dot bracket notation (DBN) (Figures 3E and 3F). To search for preferred features in IP-enriched structures, for every structure and each position based on DBN, we checked whether it is part of the stem, is in a loop, is in a bulge or if it is in between two stems. Then we compared the proportion of the structures having nucleotide at specific position in a stem, loop, etc, in between IP-enriched structures, IP-depleted structures and structures with no difference in their abundance between m^{6}A-IP and input (Figure 3G). In IP-enriched structures we observed a clear preference of A at position 15 (in the motif UAC<u>A</u>GAGAA), which is methylated by METTL16, to be in a single nucleotide bulge or to lie in a region surrounded by stem structures (Figure 3G). To see the differences in IP-enriched and depleted structures, we also plotted the log2 difference of the frequencies for IP-enriched (or depleted) structures when related to the structures not differentially represented between m^{6}A IP and input (Figure 3H). While the IP-enriched structures had higher proportion of 15A in a single nucleotide bulge or lying between two stems, the IP-depleted structures showed the opposite trend, with less proportion of structures with 15A in a bulge or in between two stems. For calculations of these log2 ratios of the frequencies, the frequencies lower than 0.5% were considered to be 0.5. Proportion of individual structures in which 15A can be found is summarized in a barplot (Figure 3I). We also directly compared the frequencies of oligos with individual positions in a stem, loop etc. in between m^{6}A-IP libraries and input libraries (Figure S3C) and their log2 ratios, separately for both replicas (Figure S3D). In m^{6}A-IP libraries we observed increased proportion of oligos where 15A is in single nucleotide bulge or in between two stems.

To check whether there is specific sequence preference outside the TAC<u>A</u>GAGAA consensus motif, we compared the nucleotide frequencies at individual oligo positions between IP and input samples and plotted their log2 ratios (Figure S3E). In the IP-oligos we observed general higher frequencies of G and C. We used MEME - Motif discovery tool 4.11.2 (Bailey and Elkan, 1994) to search for any sequence motif in the IP enriched left 11-mers and right 10-mers surrounding the TAC<u>A</u>GAGAA (Figure S3F).

Transcriptome analysis of Mettl16 mutant mouse embryos

Paired-end reads were sorted into individual libraries based on the barcodes and aligned to NCBI RefSeq transcripts (build mm10) using Salmon v0.7.2 (Patro et al., 2017). The genotype of the mouse embryos giving rise to the individual samples was assessed based on the presence of the reads derived from WT *Mettl16* allele (containing CACCAGGATTCGGACAAAACTA or TAGTTTTGTCC GAATCCTGGTG sequence, since libraries are non-strand-specific) and from *Mettl16* KO allele (containing TCACCAATGTAAATA GATGAGG or CCTCATCTATTTACATTGGTGA sequence). For E2.5 there were 5 *Mettl16*$^{+/+}$ (WT), 14 *Mettl16*$^{+/-}$ (HET) and 12 *Mettl16*$^{-/-}$ (KO) samples. For E3.5 we got 18 *Mettl16*$^{+/+}$ (WT), 9 *Mettl16*$^{+/-}$ (HET) and 12 *Mettl16*$^{-/-}$ (KO) samples

The transcript estimates were imported into DESeq2 1.18.1 (Love et al., 2014) and summarized to gene levels using tximport 1.2.0 (Soneson et al., 2015). The DESeq2 was used to obtain lists of differentially expressed genes with statistical significance (adjusted p value $\leq$ 0.1). The MA plots were plotted using graphics::smoothScatter function and the individual genes with significantly different expression were highlighted (Figures 4D and 5C).

For the E2.5 dataset, twenty genes were found to be differentially expressed between some of the genotypes (Figure 4E; Table S3) and their expression was visualized by heatmap using the made4::heatplot function (Figure 4E). Only four of the genes had significantly different expression in *Mettl16*$^{-/-}$ versus *Mettl16*$^{+/-}$ and also in *Mettl16*$^{-/-}$ versus *Mettl16*$^{+/+}$ comparison (Figure S5B). Boxplots of normalized counts were plotted for those genes, with individual samples plotted as dots using graphics:stripchart function (Figures 4F and S5C). To visualize the coverage of individual genomic loci, the sequenced reads were aligned to reference mm10

genome using STAR (Dobin et al., 2013) and the normalized coverage was calculated using GenomicRanges::coverage function. Mean coverages were plotted for individual genotypes using Gviz 1.22.3 (Hahne and Ivanek, 2016) together with the transcript annotation obtained either from NCBI RefSeq track from UCSC or from GENCODE M17 (Figures 4G and S5E). Gviz was also used to plot the coverage of individual exons or introns. To compare the amount of reads coming from individual introns of *Mat2a*, featureCounts (Liao et al., 2014) was used to obtain the counts for individual genomic exons and introns which were then normalized by DESeq2. Boxplots of the counts were plotted for individual introns of *Mat2a* normalized to library sizes or to overall *Mat2a* counts (Figure S5D). The intron coordinates used were shortened by 10 nucleotides from both sides so that the intron counts were not affected by exonic reads partially protruding into the introns. The JunctionSeq 1.8.0 (Hartley and Mullikin, 2016) was used to search for differential usage of splice junctions among the genotypes. Only few splice junctions were significantly (adjusted p value $\leq$ 0.01) differentially used between *Mettl16*$^{-/-}$ versus *Mettl16*$^{+/-}$ and also in *Mettl16*$^{-/-}$ versus *Mettl16*$^{+/+}$ comparison (Table S3). Whereas the reads spanning the splice junction of last *Mat2a* (NM_145569 = ENSMUST00000059472.9) intron were depleted in *Mettl16*$^{-/-}$ when normalized to overall *Mat2a* transcript levels, alternative splice junction (common for ENSMUST00000206904.1 and ENSMUST00000206692.1) was elevated (Figure 4H) as shown by boxplots. This was accompanied by overall increase of last *Mat2a* intron counts in *Mettl16*$^{-/-}$ which is however not significant.

For the E3.5 dataset, 5166 genes were found to be differentially expressed between some of the genotypes (Figure 5D; Table S3) and their expression was visualized by heatmap using the made4::heatplot function (Figure 5D). Most of the genes which were found to be dysreguled in *Mettl16*$^{-/-}$ versus *Mettl16*$^{+/-}$ were also differentially expressed between *Mettl16*$^{-/-}$ and *Mettl16*$^{+/+}$ (Figure 5E). Enriched gene ontology biological processes for upregulated and downregulated genes were identified by ENRICHR (Chen et al., 2013; Kuleshov et al., 2016) and are summarized in Table S3. Boxplots of normalized counts were plotted for *Mettl16*, *Mat2a* and top dysregulated genes with individual samples plotted as dots using graphics:stripchart function (Figures 5B and S6D). FeatureCounts was used to obtain summarized counts for introns, exons and repeats which did not show any differences between the genotypes neither in E2.5 nor in E3.5 (Figures S5A and 5G) and also to obtain the counts for individual genomic exons and introns. Boxplot was used to compare number of reads arising from last intron of *Mat2a* and splice junction reads crossing the last intron (Figure S6A) whose counts were obtained from JunctionSeq analysis. All the splice junctions which were significantly (adjusted p value $\leq$ 0.01) differentially used between *Mettl16*$^{-/-}$ versus *Mettl16*$^{+/-}$ and also in *Mettl16*$^{-/-}$ versus *Mettl16*$^{+/+}$ comparison are summarized in Table S3. Overall counts of uniquely mapping reads crossing the splice junctions were obtained from SJ.out.tab files generated by STAR and their proportion was compared between individual samples (Figure 5F). To find out whether genes specific to any developmental stage are misregulated in the mutant, we checked the expression of the key transcription and chromatin factors characteristic for different stages (Table S1 of Mohammed et al., 2017). Heatmap of log2 (normalized counts +1) expression was plotted using gplots::heatmap.2 from individual samples (Figure S6B). Boxplots were used to compare the average expression change between *Mettl16*$^{-/-}$ versus *Mettl16*$^{+/-}$ and *Mettl16*$^{-/-}$ versus *Mettl16*$^{+/+}$, with individual genes plotted as dots (Figure S6C).

DATA AND SOFTWARE AVAILABILITY

Deep sequencing data generated in this study are deposited with Gene Expression Omnibus under the accession number GSE116329. Crystallographic data are deposited with Protein Data Bank under PDB accessions: 6GFN, 6GT5 and 6GFK. Other raw data associated with this study are deposited with Mendeley Data under the accession https://doi.org/10.17632/ny82j2ngt5.1. The *Mettl16* knockout mutant mouse generated in this study will be available from the Lead Contact.

Molecular Cell, Volume *71*

Supplemental Information

Methylation of Structured RNA by the m^6A Writer METTL16 Is Essential for Mouse Embryonic Development

Mateusz Mendel, Kuan-Ming Chen, David Homolka, Pascal Gos, Radha Raman Pandey, Andrew A. McCarthy, and Ramesh S. Pillai

INVENTORY OF SUPPLEMENTAL INFORMATION

One PDF with

1) Supplemental Figures (S1-6)

2) Supplemental figure legends (S1-6)

3) Supplemental Tables (S1-2) and legends

Supplemental Figures

Figure S1. Full-length human METTL16 exists as monomers. Related to Figure 1.

Figure S2. Mutational analysis of the human METTL16-core methyltransferase domain to define the RNA-binding groove. Related to Figures 1 and 2.

Figure S3. In vitro methylation with human METTL16-FL and a randomized RNA library reveals structural and sequence requirements for m^6A RNA methylation. Related to Figure 3.

Figure S4. Embryonic lethality in *Mettl16* knockout mice around implantation stage. Related to Figure 4.

Figure S5. Lack of METTL16 has very specific and limited effect on the transcriptome of E2.5 embryos. Related to Figure 4.

Figure S6. Dramatically altered transcriptome of E3.5 *Mettl16* knockout embryos. Related to Figure 5.

Supplemental Tables

Table S1. DNA primers and RNA oligonucleotides used in this study. Related to STAR Methods and Figure 1,2 and 3.

Table S2. List of all deep-sequencing libraries created in this study. Related to STAR Methods and Figure 4 and 5.

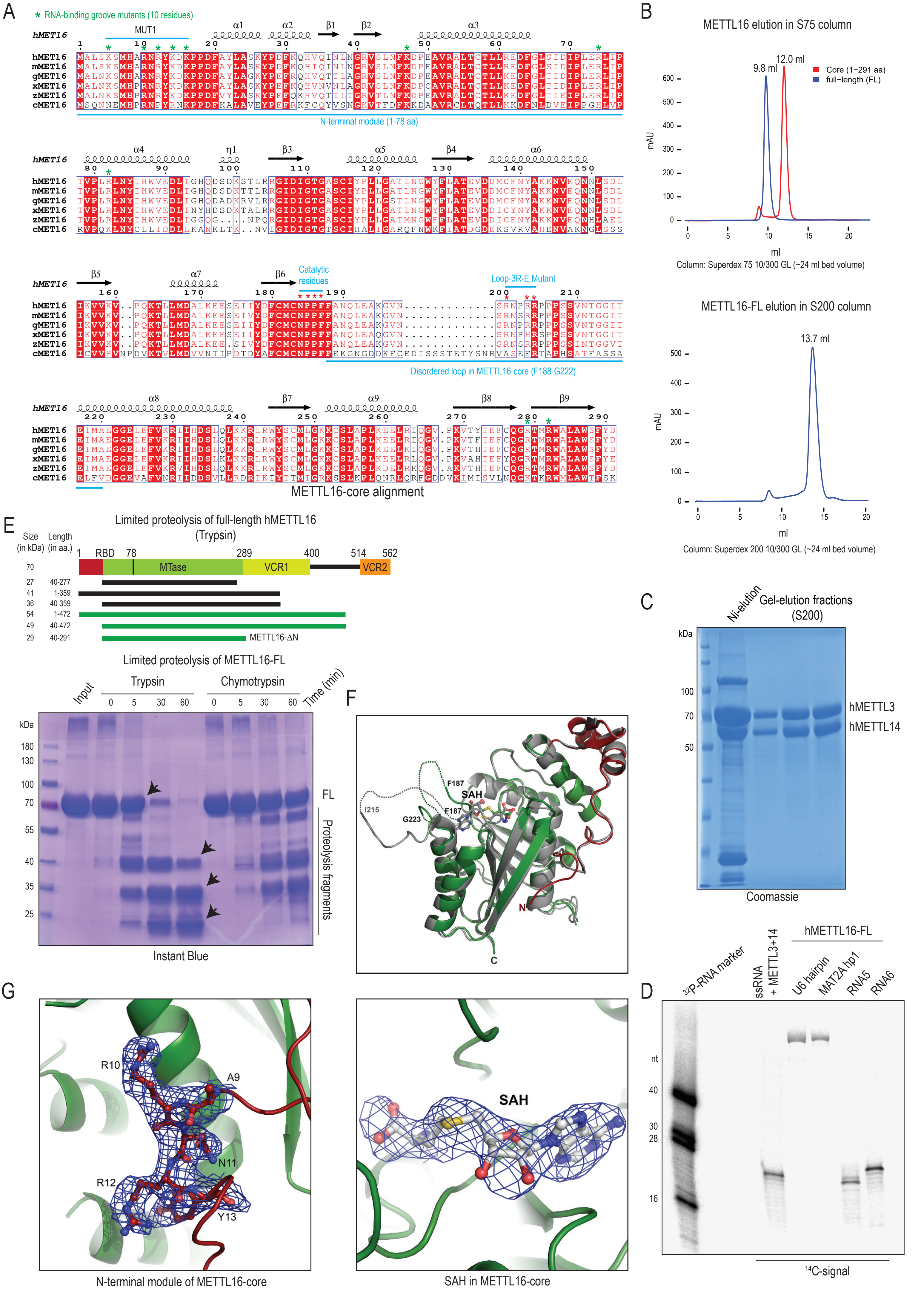
A
* RNA-binding groove mutants (10 residues)
MUT1
hMET16
mMET16
gMET16
xMET16
zMET16
cMET16
N-terminal module (1-78 aa)
Catalytic residues
Loop-3R-E Mutant
Disordered loop in METTL16-core (F188-G222)
METTL16-core alignment
B
METTL16 elution in S75 column
9.8 ml
12.0 ml
Core (1−291 aa)
full−length (FL)
mAU
ml
Column: Superdex 75 10/300 GL (~24 ml bed volume)
METTL16-FL elution in S200 column
13.7 ml
Column: Superdex 200 10/300 GL (~24 ml bed volume)
C
Ni-elution
Gel-elution fractions (S200)
kDa
hMETTL3
hMETTL14
Coomassie
D
32P-RNA marker
ssRNA + METTL3+14
hMETTL16-FL
U6 hairpin
MAT2A hp1
RNA5
RNA6
nt
14C-signal
E
Limited proteolysis of full-length hMETTL16 (Trypsin)
Size (in kDa)
Length (in aa.)
RBD
MTase
VCR1
VCR2
METTL16-ΔN
Limited proteolysis of METTL16-FL
Input
Trypsin
Chymotrypsin
Time (min)
FL
Proteolysis fragments
Instant Blue
F
F187
SAH
I215
G223
N
C
G
R10
A9
N11
R12
Y13
N-terminal module of METTL16-core
SAH in METTL16-core

Figure-S1

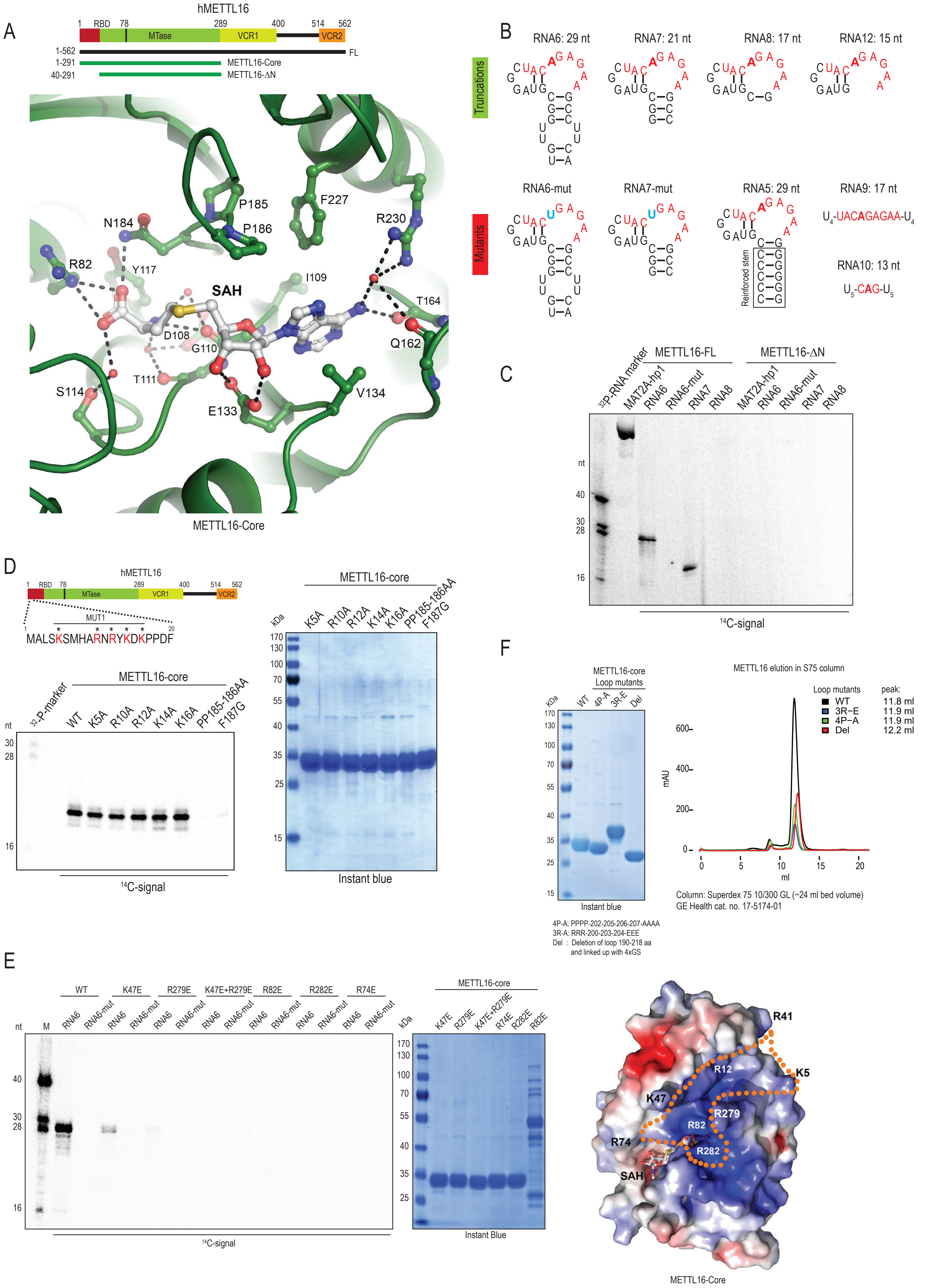

A
hMETTL16
RBD
MTase
VCR1
VCR2
FL
METTL16-Core
METTL16-ΔN
SAH
R82
N184
P185
P186
F227
R230
Y117
I109
D108
G110
T111
T164
Q162
S114
E133
V134
METTL16-Core
B
RNA6: 29 nt
RNA7: 21 nt
RNA8: 17 nt
RNA12: 15 nt
Truncations
RNA6-mut
RNA7-mut
RNA5: 29 nt
RNA9: 17 nt
RNA10: 13 nt
Mutants
Reinforced stem
C
METTL16-FL
METTL16-ΔN
32P-RNA marker
MAT2A-hp1
RNA6
RNA6-mut
RNA7
RNA8
nt
14C-signal
D
hMETTL16
MUT1
MALSKSMHARNRYKDKPPDF
METTL16-core
32-P-marker
WT
K5A
R10A
R12A
K14A
K16A
PP185-186AA
F187G
14C-signal
METTL16-core
kDa
Instant blue
E
WT
K47E
R279E
K47E+R279E
R82E
R282E
R74E
M
RNA6
RNA6-mut
14C-signal
METTL16-core
Instant Blue
R41
R12
K5
K47
R279
R82
R74
R282
SAH
METTL16-Core
F
METTL16-core
Loop mutants
4P-A
3R-E
Del
Instant blue
4P-A: PPPP-202-205-206-207-AAAA
3R-A: RRR-200-203-204-EEE
Del : Deletion of loop 190-218 aa and linked up with 4xGS
METTL16 elution in S75 column
Loop mutants
peak:
WT 11.8 ml
3R-E 11.9 ml
4P-A 11.9 ml
Del 12.2 ml
mAU
ml
Column: Superdex 75 10/300 GL (~24 ml bed volume)
GE Health cat. no. 17-5174-01

Figure-S2

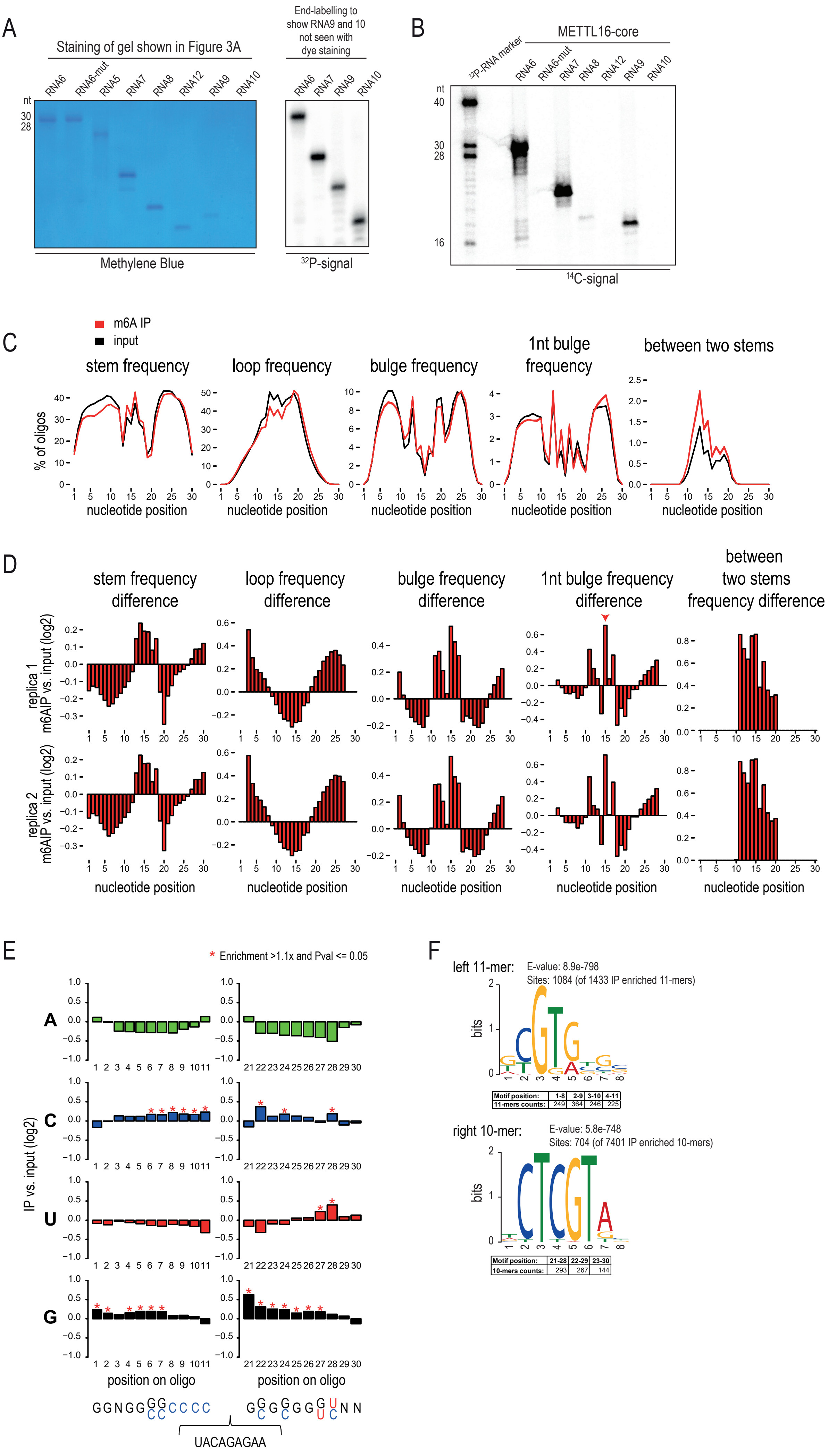
A
Staining of gel shown in Figure 3A
RNA6 RNA6-mut RNA5 RNA7 RNA8 RNA12 RNA9 RNA10
nt
30
28
Methylene Blue
End-labelling to show RNA9 and 10 not seen with dye staining
RNA6 RNA7 RNA9 RNA10
32P-signal
B
METTL16-core
32P-RNA marker RNA6 RNA6-mut RNA7 RNA8 RNA12 RNA9 RNA10
nt
40
30
28
16
14C-signal
C
m6A IP
input
stem frequency
loop frequency
bulge frequency
1nt bulge frequency
between two stems
% of oligos
nucleotide position
D
stem frequency difference
loop frequency difference
bulge frequency difference
1nt bulge frequency difference
between two stems frequency difference
replica 1 m6AIP vs. input (log2)
replica 2 m6AIP vs. input (log2)
nucleotide position
E
* Enrichment >1.1x and Pval <= 0.05
A
C
U
G
IP vs. input (log2)
position on oligo
GGNGG GG CC CCCC
GG C G C GG G U U C NN
UACAGAGAA
F
left 11-mer:
E-value: 8.9e-798
Sites: 1084 (of 1433 IP enriched 11-mers)
bits
Motif position: 1-8 2-9 3-10 4-11
11-mers counts: 249 364 246 225
right 10-mer:
E-value: 5.8e-748
Sites: 704 (of 7401 IP enriched 10-mers)
bits
Motif position: 21-28 22-29 23-30
10-mers counts: 293 267 144

Figure-S3

A

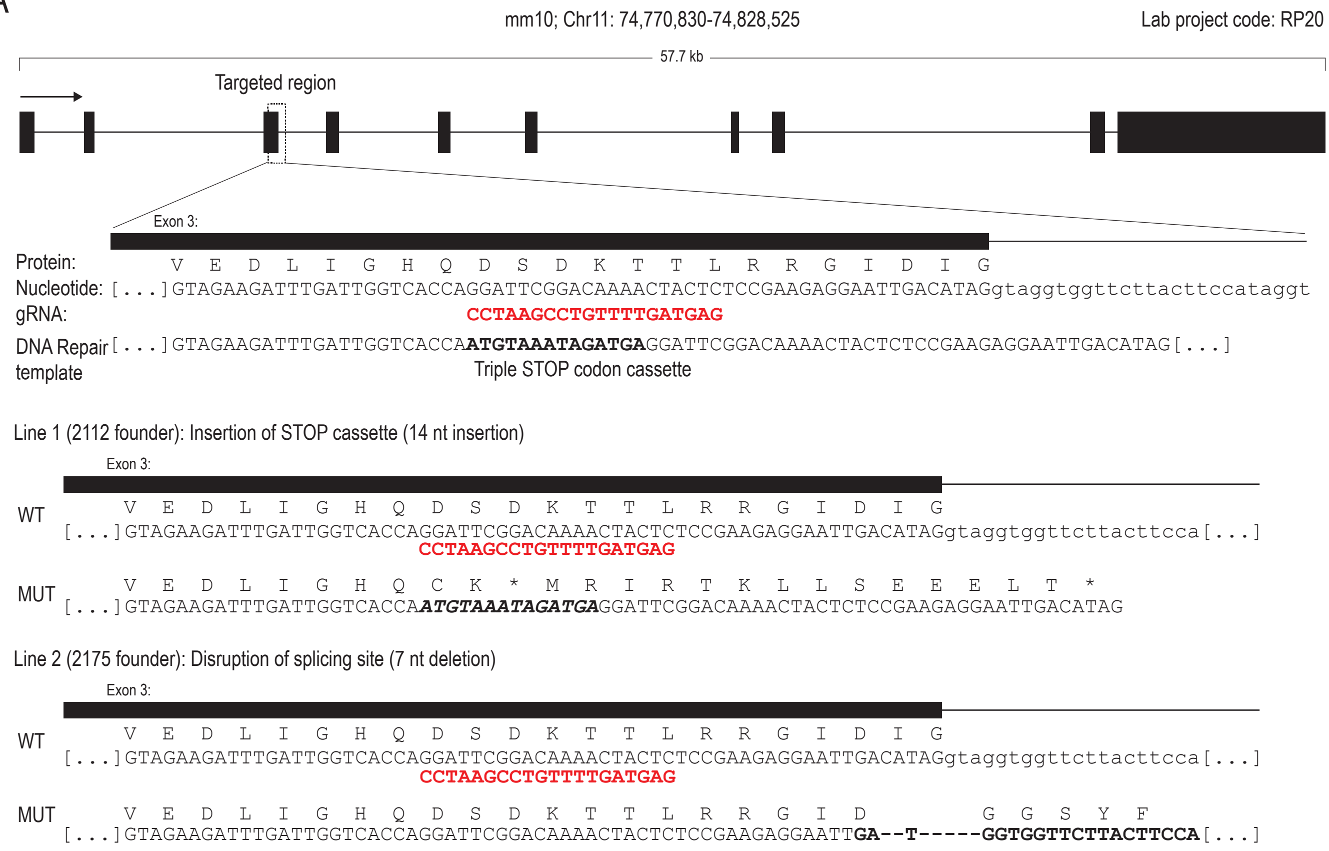

B

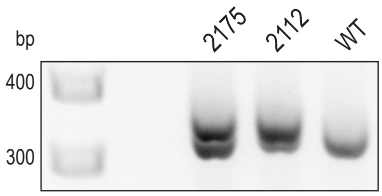

C

Genotype of animals in litters at weaning age (P21) from *Mettl16+/-* x *Mettl16+/-* crosses

	Total	KO	HET	WT	Male	Female
**Number**	**156**	0	114	42	73	83
**Percentage**	**-**	0.0%	73.1%	26.9%	46.8%	53.2%

D

Genotype of E6.5 embryos from *Mettl16+/-* x *Mettl16+/-* crosses

	Placentas	Embryos	WT	HET	KO	Unknown
**Number**	68	52	16	32	1	3
**Percentage**	-	76.5%	30.8%	61.5%	1.9%	5.8%

E

Genotype of E8.5 embryos from *Mettl16+/-* x *Mettl16+/-* crosses

	Placentas	Embryos	WT	HET	KO
**Number**	24	14	1	13	0
**Percentage**	-	58%	7%	93%	

F

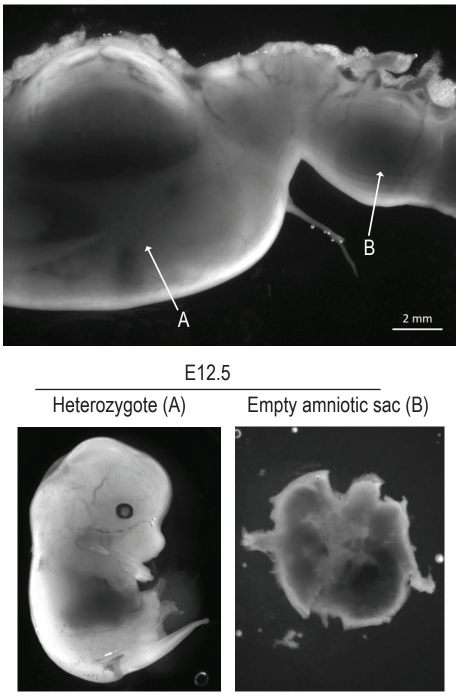

Figure-S4

A

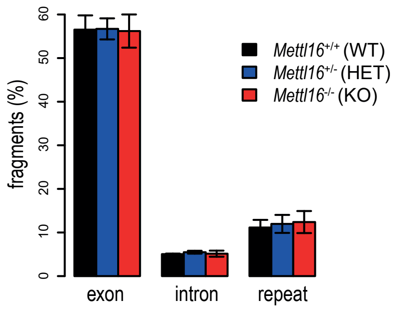

B

	log2 fold change			adjusted p-value		
gene	KO vs WT	KO vs HET	HET vs WT	KO vs WT	KO vs HET	HET vs WT
Ccdc92b	-1.63	-1.39	-0.24	4.79E-02	6.43E-03	1.00E+00
Mettl16	-1.83	-1.32	-0.52	4.31E-11	3.13E-10	1.00E+00
Mat2a	-2.41	-2.00	-0.41	2.69E-31	5.03E-40	1.00E+00
Gm15698	-2.58	-2.16	-0.41	6.40E-07	1.65E-09	1.00E+00

C

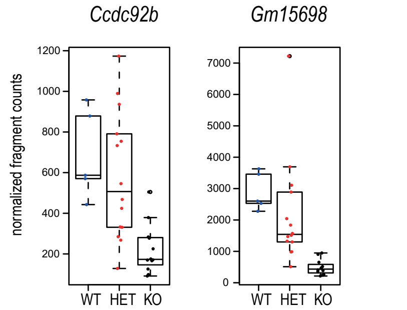

D

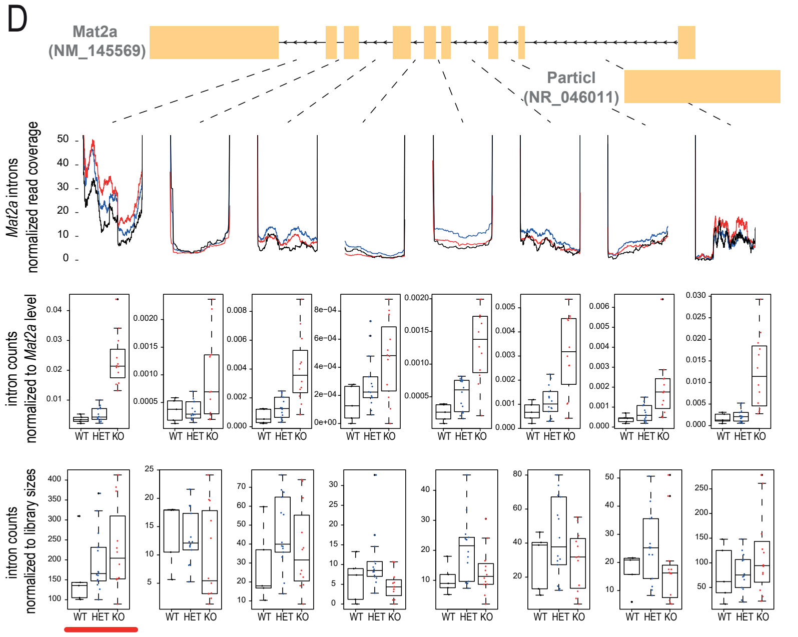

E

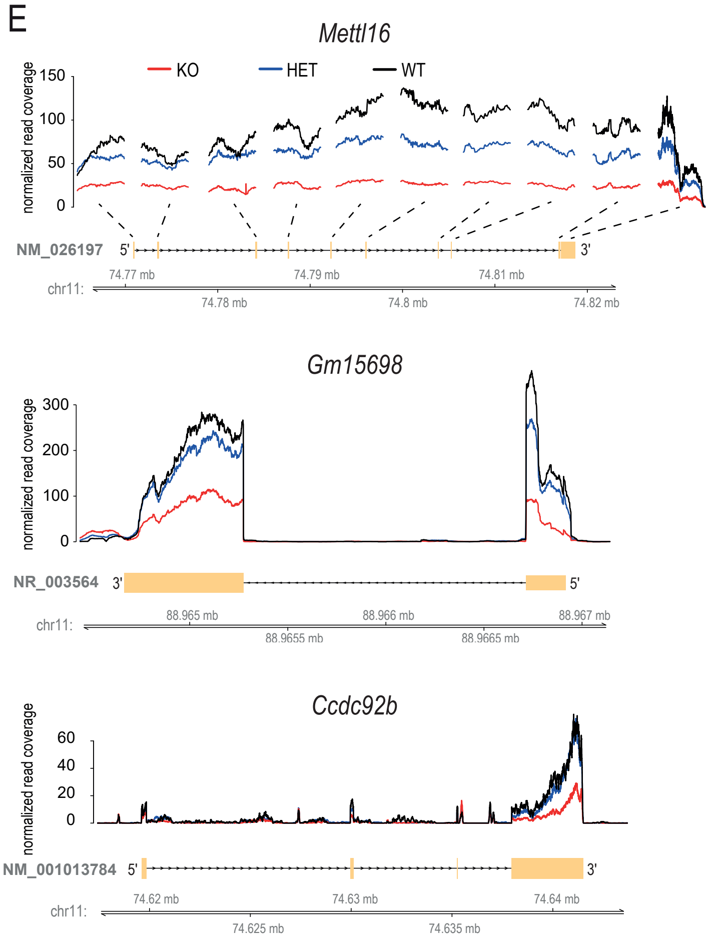

F

METTL16 Complex from HEK293 cells

No.	Name	ID	MW [kDa]	Exclusive Unique Peptide Count: METTL16 Rep. 1	METTL16 Rep. 2	METTL16 Rep. 3	GFP Rep. 1	GFP Rep. 2
1	Kanadaptin	SLC4A1AP	89	26	24	18	4	0
2	U6 small nuclear RNA (adenine-(43)-N(6))-methyltransferase	METTL16	64	74	84	77	13	5
3	Lupus La protein	SSB	47	9	7	4	0	2
4	DNA repair protein RAD50	RAD50	154	5	3	6	0	2
5	Splicing factor 3B subunit 2	SF3B2	100	19	17	15	5	2
6	Deoxynucleoside triphosphate triphosphohydrolase SAMHD1	SAMHD1	72	4	9	7	0	3
7	Splicing factor 3B subunit 1	SF3B1	146	34	21	33	9	6
8	Kinesin-like protein KIF18B	KIF18B	94	4	6	6	1	2
9	Structural maintenance of chromosomes protein 4	SMC4	147	4	3	4	0	0
10	Zinc finger CCCH domain-containing protein 8	ZC3H8	34	2	5	4	0	2
11	WD repeat-containing protein 82	WDR82	35	4	2	5	2	0
12	Splicing factor 3B subunit 3	SF3B3	136	23	8	22	6	6
13	Furin	FURIN	87	3	5	5	0	3
14	Probable 28S rRNA (cytosine(4447)-C(5))-methyltransferase	NOP2	89	2	7	3	2	0
15	Galectin-1	LGALS1	15	2	3	6	2	0

G

METTL16 Complex from mouse tissues

No.	Name	ID	MW [kDa]	Exclusive Unique Peptide Count: Testis	Testis	Spleen	IgG CTRL	IgG CTRL
1	Splicing factor 3B subunit 1	SF3B1	146	28	33	51	0	0
2	Splicing factor 3B subunit 3	SF3B3	136	21	25	33	0	0
3	Protein PRRC2A	PRRC2A	229	24	32	13	0	0
4	Kinesin-like protein KIF13B	KIF13B	203	24	23	7	0	0
5	Probable ATP-dependent RNA helicase DDX5	DDX5	69	22	20	33	0	0
6	Cleavage and polyadenylation specificity factor subunit 1	CPSF1	161	28	19	22	0	0
7	pre-mRNA 3' end processing protein WDR33	WDR33	145	24	19	22	0	0
8	Splicing factor 3B subunit 2	SF3B2	100	20	17	28	0	0
9	Splicing factor 3A subunit 1	SF3A1	89	10	19	30	0	0
10	Protein scribble homolog	SCRIB	174	19	21	5	0	0
11	Gem-associated protein 5	GEMIN5	167	21	13	18	0	0
12	U6 small nuclear RNA (adenine-(43)-N(6))-methyltransferas	METTL16	62	17	17	14	0	0
13	Serine/arginine repetitive matrix protein 2	SRRM2	295	12	28	16	0	0
14	Cleavage and polyadenylation specificity factor subunit 2	CPSF2	88	21	14	16	0	0
15	Heterogeneous nuclear ribonucleoprotein M	HNRNPM	78	19	14	15	0	0

Figure-S5

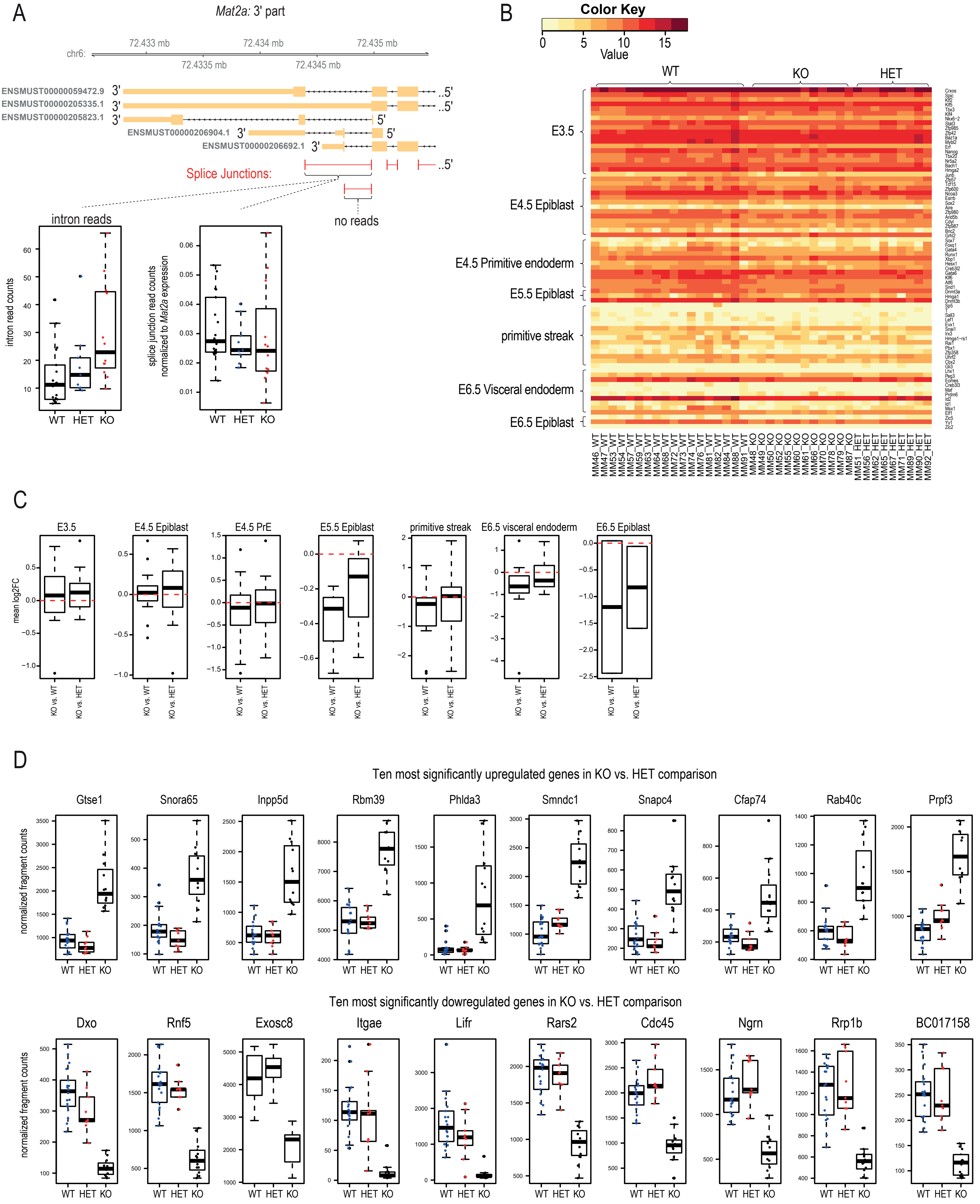
A
Mat2a: 3' part
chr6:
72.433 mb
72.434 mb
72.435 mb
72.4335 mb
72.4345 mb
ENSMUST00000059472.9
ENSMUST00000205335.1
ENSMUST00000205823.1
ENSMUST00000206904.1
ENSMUST00000206692.1
Splice Junctions:
no reads
intron reads
intron read counts
splice junction read counts normalized to Mat2a expression
WT HET KO
B
Color Key
Value
WT
KO
HET
E3.5
E4.5 Epiblast
E4.5 Primitive endoderm
E5.5 Epiblast
primitive streak
E6.5 Visceral endoderm
E6.5 Epiblast
C
E3.5
E4.5 Epiblast
E4.5 PrE
E5.5 Epiblast
primitive streak
E6.5 visceral endoderm
E6.5 Epiblast
mean log2FC
KO vs. WT
KO vs. HET
D
Ten most significantly upregulated genes in KO vs. HET comparison
Gtse1
Snora65
Inpp5d
Rbm39
Phlda3
Smndc1
Snapc4
Cfap74
Rab40c
Prpf3
normalized fragment counts
Ten most significantly dowregulated genes in KO vs. HET comparison
Dxo
Rnf5
Exosc8
Itgae
Lifr
Rars2
Cdc45
Ngrn
Rrp1b
BC017158
normalized fragment counts

Figure-S6

Table S1. DNA and RNA oligonucleotides used in this study.
Related to STAR Methods and Figure 1,2 and 3.

**DNA oligos**		
**Name**	**Sequence**	**Comments**
CRISPR F primer	GAAATTAATACGACTCACTATAGGGAGTAGTTTTGTCCGAATCCG TTTTAGAGCTAGAAATAGC	RRoligo770
CRISPR sgR primer	CAAAATCTCGATCTTTATCGTTCAATTTTATTCCGATCAGGCAAT AGTTGAACTTTTTCACCGTGGCTCAGCCACGAAAA	
MMoligo109	ATTCCCACAGTCCCCTTGAG	Genotyping PCR
MMoligo110	CAATGCCCAACCAACACAGA	Genotyping PCR
XXXXX	AGTTGAGAATGCAAAACCTATGGAAGTAAGAACCACCTACCTATG TCAATTCCTCTTCGGAGAGTAGTTTTGTCCGAATCC***TCATCTATT TACAT***TGGTGACCAATCAAATCTTCTACCCAGTGAATATAGTTAA GTCTCAAGGGGACTGTGGGAATTAGTCTCTCCAAA	ssDNA repair template
MMoligo81	TAATACGACTCACTATAGGGTGTTAGCCTTTTTTCCCCAGACTTG TTGGCGTAGGCTACA	MAT2A hp1 template forward
MMoligo82	GGAAGGAGGGCCCTTTCCCTCAGAGCTTGAAGGCTTCTCTGTAGC CTACGCCAACAAGTC	MAT2A hp1 template reverse
MMoligo83	TAATACGACTCACTATAGGGAAAATTGGAACGATACAGAGAAGAT TAGCATGGCCCCTGC	U6 snRNA template forward
MMoligo84	AAAATATGGAACGCTTCACGAATTTGCGTGTCATCCTTGCGCAGG GGCCATGCTAATCTT	U6 snRNA template reverse
**RNA oligos**		
**Lab Name**	**Sequence**	**Name in this study**
MMRNA6	UGUUGGCGUAGGCUACAGAGAAGCCUUCA	RNA6 ; 29nt
MMRNA11	UGUUGGCGUAGGCUACUGAGAAGCCUUCA	RNA6-mut ; 29nt
MMRNA7	GGCGUAGGCUACAGAGAAGCC	RNA7 ; 21nt
MMRNA8	CGUAGGCUACAGAGAAG	RNA8 ; 17nt
MMRNA5	CCCCCCCGUAGGCUACAGAGAAGGGGGGG	RNA5 ; 29nt
MMRNA12	GUAGGCUACAGAGAA	RNA12 ; 15nt
MMRNA9	UUUUUACAGAGAAUUUU	RNA9 ; 17nt
MMRNA10	UUUUUCAGUUUUU	RNA10 ;13nt
MMRNA16	GGGGUACAGAGAAGGGG	RNA16 ; 17nt
MMRNA14	N11-UACAGAGAA-N10	Library;30nt; N= randomized
MMRNA21	CAGUAGCGACGUACAGAGAAACAUCUUCUC	RNA21 ; 30nt
MMRNA22	CAGUAGCGACGUACUGAGAAACAUCUUCUC	RNA22 ; 30nt
MMRNA23	CCGGCUUAGCCUACAGAGAACCUUUCUCGU	RNA23 ; 30nt
MMRNA24	CCGGCUUAGCCUACUGAGAACCUUUCUCGU	RNA24 ; 30nt
MMRNA28	GGGCGUAGGCNACANNNNNGCCC	RNA28 ; 23 nt ; N= randomized
MMRNA29	GGGCNNAGGCNNNANNNNNGCCC	RNA29 ; 23 nt ; N= randomized
MMRNA30	GGCGUAGGCUACAGACUUGCC	RNA30 ; 21 nt
MET1 RNA	UACACUCGAUCUGGACUAAAGCUGCUC	METTL3/14 substrate
MET2 RNA	UACACUCGAUCUGGAUUAAAGCUGCUC	METTL3/14 substrate(mut)
RP_RNA_1	UGACAUGAACACAGGUGCUCAGAUAGCUUU	RNA marker- 30nt
RP_RNA_3	UGACAUGAACACAGGUGCUCAGAUAGCU	RNA marker-28nt
RP_RNA_18	AGCACCGUAAAGACGC	RNA marker-16nt
RP_RNA_19	GCGUCUUUACGGUGCUUAAAACAAAACAAAACAAAACAAA	RNA marker-40nt

Supplemental Table S2. List of all deep-sequencing libraries created in this study. Related to STAR Methods

experiment	sample	*Mettl16* genotype	reads
RNASeq of E2.5 embryo	MM14	HET	34679836
RNASeq of E2.5 embryo	MM15	HET	28734242
RNASeq of E2.5 embryo	MM16	KO	34160424
RNASeq of E2.5 embryo	MM17	KO	36085991
RNASeq of E2.5 embryo	MM18	HET	33601058
RNASeq of E2.5 embryo	MM19	HET	32744958
RNASeq of E2.5 embryo	MM20	KO	36987647
RNASeq of E2.5 embryo	MM21	HET	33169466
RNASeq of E2.5 embryo	MM22	HET	32156604
RNASeq of E2.5 embryo	MM23	KO	32000383
RNASeq of E2.5 embryo	MM24	KO	30556292
RNASeq of E2.5 embryo	MM25	WT	23023349
RNASeq of E2.5 embryo	MM26	HET	33038807
RNASeq of E2.5 embryo	MM27	WT	31126991
RNASeq of E2.5 embryo	MM28	HET	32684662
RNASeq of E2.5 embryo	MM29	KO	29378767
RNASeq of E2.5 embryo	MM31	HET	35120157
RNASeq of E2.5 embryo	MM32	WT	32219943
RNASeq of E2.5 embryo	MM33	HET	32009135
RNASeq of E2.5 embryo	MM34	KO	32613795
RNASeq of E2.5 embryo	MM35	KO	29911795
RNASeq of E2.5 embryo	MM36	KO	35371390
RNASeq of E2.5 embryo	MM37	HET	34461243
RNASeq of E2.5 embryo	MM38	KO	34035525
RNASeq of E2.5 embryo	MM39	HET	33553354
RNASeq of E2.5 embryo	MM40	KO	31275672
RNASeq of E2.5 embryo	MM41	KO	32321617
RNASeq of E2.5 embryo	MM42	WT	34131480
RNASeq of E2.5 embryo	MM43	HET	33347148
RNASeq of E2.5 embryo	MM44	HET	35295970
RNASeq of E2.5 embryo	MM45	WT	25935853

experiment	sample	*Mettl16* genotype	reads
RNASeq of E3.5 embryo	MM46	WT	27418677
RNASeq of E3.5 embryo	MM47	WT	27497901
RNASeq of E3.5 embryo	MM48	KO	30804681
RNASeq of E3.5 embryo	MM49	KO	31884501
RNASeq of E3.5 embryo	MM50	KO	34965952
RNASeq of E3.5 embryo	MM51	HET	33312807
RNASeq of E3.5 embryo	MM52	KO	28476667
RNASeq of E3.5 embryo	MM53	WT	31001004
RNASeq of E3.5 embryo	MM54	WT	26071380
RNASeq of E3.5 embryo	MM55	KO	31392715
RNASeq of E3.5 embryo	MM56	HET	26518367
RNASeq of E3.5 embryo	MM57	WT	36455849
RNASeq of E3.5 embryo	MM59	WT	35482923
RNASeq of E3.5 embryo	MM60	KO	31141823
RNASeq of E3.5 embryo	MM61	KO	30971406
RNASeq of E3.5 embryo	MM62	HET	31630583
RNASeq of E3.5 embryo	MM63	WT	42925667
RNASeq of E3.5 embryo	MM64	WT	46540640
RNASeq of E3.5 embryo	MM65	HET	47186347
RNASeq of E3.5 embryo	MM66	KO	46106111
RNASeq of E3.5 embryo	MM67	HET	42360160
RNASeq of E3.5 embryo	MM68	WT	44228364
RNASeq of E3.5 embryo	MM70	KO	34314476
RNASeq of E3.5 embryo	MM71	HET	38949449
RNASeq of E3.5 embryo	MM72	WT	38861981
RNASeq of E3.5 embryo	MM73	WT	51611166
RNASeq of E3.5 embryo	MM74	WT	50932521
RNASeq of E3.5 embryo	MM76	WT	45598521
RNASeq of E3.5 embryo	MM78	KO	27609275
RNASeq of E3.5 embryo	MM79	KO	38122632
RNASeq of E3.5 embryo	MM81	WT	43648518
RNASeq of E3.5 embryo	MM82	WT	37246976
RNASeq of E3.5 embryo	MM83	NA	39136258
RNASeq of E3.5 embryo	MM84	WT	36941697
RNASeq of E3.5 embryo	MM87	KO	24036687
RNASeq of E3.5 embryo	MM88	WT	103815156
RNASeq of E3.5 embryo	MM89	HET	34466973
RNASeq of E3.5 embryo	MM90	HET	62009799
RNASeq of E3.5 embryo	MM91	WT	39750111
RNASeq of E3.5 embryo	MM92	HET	39921773

experiment	sample	description	reads	filtered reads
METTL16 in vitro IP and RNASeq	RR582	Input sample 11	42755991	21312366
METTL16 in vitro IP and RNASeq	RR583	Input sample 12	37993827	18869613
METTL16 in vitro IP and RNASeq	RR586	m6A IP sample 11	42519953	23104554
METTL16 in vitro IP and RNASeq	RR587	m6A IP sample 12	36825565	1203361

SUPPLEMENTAL FIGURE LEGENDS

Figure S1. Full-length human METTL16 exists as monomers. Related to Figure 1.

(A) Protein sequence alignment of the methyltransferase domain of METTL16 proteins. MET16, METTL16; h, human (mammal); m, mouse (mammal); g, *Gallus* (bird); x, *Xenopus* (amphibian); z, zebrafish (fish); c, *C.elegans* (nematode). The secondary structure features present in the human METTL16-core (PDB ID: 6GFN) are indicated above: α helices, β-strands and η-3_{10} helix. Residues (marked with green asterisks) in the putative RNA-binding groove where mutated and shown to affect methylation activity and/or RNA-binding (tested for MUT1 only, in Figure 2F). Deletion of the disordered loop or other mutated residues (red asterisks) also abolish activity. (B) Gel-filtration chromatography profile for indicated proteins on two different Superdex columns (S75 and S200). The elution profile of the proteins are consistent with them being a monomer. The full-length (FL) METTL16 was produced in insect cells, while the METTL16-core was expressed in *E.coli*. (C) Purification of full-length human METTL3/METTL14 complex. See STAR Methods. (D) In vitro methylation assay with ^{14}C-SAM, METTL16-FL or the METTL3+METTL14 complex, and indicated RNAs. The single-stranded RNA (ssRNA) used for METTL3+14 reaction is MET1RNA, while full-length human U6 snRNA and full-length human *MAT2A* hairpin (hp) 1 were in vitro transcribed (STAR methods), and others were purchased (Table S1). Single-stranded RNA markers (size in nucleotides, nt) are ^{32}P-end-labelled. (E) Limited proteolysis of METTL16-FL with indicated proteases. An Instant Blue-stained SDS-PAGE with aliquots of the time-course reaction, with incubation times in minutes (min) and protein markers (in kDa) is shown. Protein boundaries of the fragments were identified by mass spectrometry from bands (indicated by arrows) in the gel. (F) Comparison of our crystal structure of the human METTL16 core (Green/Magenta; PDB ID: 6GFN) with the one recently published by Ruszkowska et al. (Grey; PDB ID: 6B92). A comparison reveals a very high degree of overlap between two structures with only minor differences (see STAR Methods). (G) Electron density for key regions identified in the METTL16-core structure. Shown on left, a 2Fo-Fc map contoured at 1.2 σ and coloured in blue for the METTL16 N-terminal region, A9 to Y13 (PDB: 6GTS). Shown on right, a 2Fo-Fc omit map contoured at 2 σ and coloured in blue for the METTL16 core SAH-binding site (PDB: 6GFN).

Figure S2. Mutational analysis of the human METTL16-core methyltransferase domain to define the RNA-binding groove. Related to Figures 1 and 2.

(A) Cartoon indicating protein domains of human METTL16. Boundaries of the two constructs crystallized in this study are shown (in green). A zoom of the catalytic domain in human METTL16-core domain showing the bound S-adenosyl-homocysteine (SAH) (PDB ID: 6GFK). Residues coordinating the SAH and the catalytic residues N184, P185, P186 are highlighted. (B) Predicted structures of various synthetic RNAs used in in vitro methylation assays. They are all derived from the human *MAT2A* hairpin (hp) 1 and the consensus methylation motif of METTL16 is highlighted (red). The adenosine (A) that is methylated is indicated in bold and this is mutated to a uridine (U) in some of the RNAs. RNA5 has a reinforced stem with artificial G:C pairs. (C) In vitro methylation assay with ^{14}C-SAM, METTL16 proteins and indicated RNAs. *MAT2A* hp1 was in vitro transcribed, while the others were purchased (Table S1). Single-stranded RNA markers (size in nucleotides, nt) are ^{32}P-end-labelled. Note that the METTL16-ΔN protein is inactive. (D) Cartoon showing the N-terminal 20 amino acids of human METTL16, with the positively charged residues that were individually mutated being highlighted (red with asterisks). In vitro methylation assay with wildtype (WT) or mutant METTL16-core versions indicated. Individual mutations of N-terminal positively charged residues to neutral alanine do not affect activity. See also Figure 2D. Catalytic-dead

mutations PP185-186AA and F187G result in absence of any activity. Quality of recombinant proteins used is shown (on the right). (E) In vitro methylation assay with human METTL16-core MTase proteins carrying mutations within the putative RNA-binding groove. The groove is outlined on the surface charge representation of the METTL16-core, on the right (this is same as shown in Figure 2C). Quality of the proteins used is shown. Note that almost all mutations abolish methylation activity. The RNA used are indicated (Table S1). (F) SDS-PAGE gel of the METTL16-core with mutations in the disordered loop. One of the proteins (Loop-3R-E) displays a retarded migration. Gel-filtration profile of the protein shows that its elution profile is same as the one seen for the wildtype protein or other mutants. See also Figure 2G.

Figure S3. In vitro methylation with human METTL16-FL and a randomized RNA library reveals structural and sequence requirements for m^{6}A RNA methylation. Related to Figure 3.

(A) Methylene blue dye staining of the same gel as shown in Figure 3A to reveal RNAs present in the reactions after in vitro methylation assays. After staining with the dye, gels were dried and exposed for detection of radioactivity, and is presented in Figure 3A. This shows the presence of RNAs of the expected sizes in all the lanes. RNA9 and 10 (uridine-rich sequences) are poorly stained with the dye. On the right, a few of the RNAs used were ^{32}P-end-labelled to reveal the integrity of the RNAs used (including RNA9 and RNA10). (B) A repetition of similar experiment as shown in Figure 3A, showing that dramatic truncations to the stem region of the *MAT2A* hairpin RNA abolishes activity. Note that RNA5 was not included in this experiment. (C) A randomized RNA library carrying the nonamer consensus sequence was incubated with METTL16-FL and m^{6}A-containing RNAs were enriched by immunoprecipitation (IP) and sequenced. See Figure 3D. Frequency of RNA oligos forming stem, loop and other selected features at individual positions is compared between m^{6}A-IP and input oligos. Methylated oligos have higher frequency of 15A (which is the adenosine predicted to be methylated in the nonamer consensus motif) in a bulge and surrounded by stems. See also Figure 3G. (D) Log$_2$ differences of oligo frequencies between m^{6}A-IP and input oligos indicate the 15A bulge and its positioning in between stem structures is important for being recognized and methylated by METTL16. Two replicate experiments show the same pattern. (E) Comparison of the nucleotide frequencies at individual oligo positions surrounding the consensus motif reveals higher G and C occurrence in methylated oligos. (F) Sequence motifs that were identified in the m^{6}A-IP-enriched 11-mers or 10-mers surrounding the consensus motif.

Figure S4. Embryonic lethality in *Mettl16* knockout mice around implantation stage. Related to Figure 4.

(A) Strategy for insertion of a triple-stop codon cassette into exon 3 of mouse *Mettl16* genomic locus using a guide RNA (gRNA) that targets the Cas9 DNA endonuclease. Homologous recombination (HR) introduces the DNA repair template with the triple-stop codon cassette into exon 3, disrupting the coding sequence, creating a knockout (KO) allele. Two independent lines were obtained and both showed identical embryonic lethality phenotype. Line#1 was used in this study for sequence analysis of embryos. (B) Genotyping PCR with mouse tail DNA from indicated mutant lines and wildtype. See STAR methods for PCR conditions. (C) Genotype of animals recovered in litters at the weaning stage (P21, post-natal day 21) from a cross of *Mettl16*$^{+/-}$ heterozygous (HET) parents. No homozygous *Mettl16*$^{-/-}$ knockout (KO) animals were present, indicating early lethality. (D) Genotyping of embryos collected at E6.5 from *Mettl16*$^{+/-}$ females crossed with *Mettl16*$^{+/-}$ males. Note that only one KO embryo was recovered. (E) Genotyping of embryos collected at E8.5 from *Mettl16*$^{+/-}$ females crossed with *Mettl16*$^{+/-}$ males. These studies reveal that loss of mouse *Mettl16* results in embryonic lethality around implantation stage. See also Figure 4B. (F) Examination of E12.5 embryos by genotyping. No KO embryos were detected, indicating early lethality. Note that only one litter was examined at E12.5. Scale bar in millimetre (mm) is shown.

Figure S5. Lack of METTL16 has very specific and limited effect on the transcriptome of E2.5 embryos. Related to Figure 4.

(A) Lack of METTL16 does not affect the global transcription from exons, introns and repeats. Error bars refer to standard deviation. (B) Only the transcripts of four genes have significantly differential abundance between in *Mettl16*$^{-/-}$ (KO) and *Mettl16*$^{+/-}$ (HET) and also between *Mettl16*$^{-/-}$ (KO) and *Mettl16*$^{+/+}$ (WT). (C) Boxplot of two genes significantly downregulated in *Mettl16*$^{-/-}$ (KO). Both of the genes lie on chromosome 11, same as the targeted *Mettl16*. (D) Normalized read coverage of *Mat2a* introns is shown together with the overall read counts for each intron. The intron read counts were normalized either just to library sizes (bottom row) or to *Mat2a* levels (middle row). One of the boxplots (marked with a red line) is reproduced in Figure 4H. (E) Normalized read coverage of the loci with three of the genes (except *Mat2a*) found to be significantly differentially expressed in *Mettl16*$^{-/-}$(KO). Only exon coverage is shown for *Mettl16*. Note that both *Gm15698* and *Ccdc92b* lie on chromosome 11 as well as *Mettl16* and their differential expression might be just the consequence of different chromosome 11 DNA sequence resulting from the mixed genetic background of parental strain which was crossed to pure B6 mouse (see STAR Methods). (F) Mass spectrometry analysis of 3xFLAG-HA-hMETTL16 immunopurified from transfected HEK293T cells. 3xFLAG-HA-GFP was used as a negative control. Presented protein hits are the top 15 most enriched proteins in the hMETTL16 IP when compared to the negative control (3xFLAG-HA-GFP). HA-tag purification was performed (STAR METHODS). Exclusive Unique Peptide Count shown. Protein threshold: 1% FDR; min. peptides: 2; Peptide threshold: 0.1% FDR. (G) Mass spectrometry analysis of endogenous mMETTL16 immunopurified from adult mouse testes and spleen. Presented protein hits are the top 15 most enriched proteins in the mMETTL16 IP when compared to the negative control. Multiple splicing factor 3B and 3A subunits are among the top candidates. Purification was done using anti-METTL16 antibody (abcam, ab186012) without RNase-treatment, negative control was beads bound with normal mouse IgG (Santa Cruz, sc-2025). Exclusive Unique Peptide Count shown. Protein threshold: 1% FDR; min. peptides: 2; Peptide threshold: 0.1% FDR.

Figure S6. Dramatically altered transcriptome of E3.5 *Mettl16* knockout embryos. Related to Figure 5.

(A) Counts of the reads spanning the splice junction (SJ) of last *Mat2a* (ENSMUST00000059472.9) intron are plotted together with read counts originating in the intron. Boxplots are shown where counts of individual samples are plotted as dots. (B) The heatmap shows the expression of transcription factors and chromatin modifiers enriched for distinct embryonic lineages [as defined by (Mohammed et al., 2017)] in individual samples. Expression of these key factors is unaltered in the *Mettl16* KO embryos. (C) Boxplots display the average log2 fold-changes (FC) between *Mettl16* mutant (KO) and control (WT or HET) mice for the groups of lineage-specific genes. Dots depict the individual genes. None of the group of lineage-specific genes seems to be differentially expressed between *Mettl16*$^{-/-}$ and *Mettl16*$^{+/-}$ or *Mettl16*$^{-/-}$ and *Mettl16*$^{+/+}$. (D) Boxplots compare the expression of the top 10 downregulated or upregulated genes in the *Mettl16*$^{-/-}$ KO blastocysts. Transcript levels of individual samples are shown as dots.

SUPPLEMENTAL TABLE LEGENDS

Table S1. DNA primers and RNA oligonucleotides used in this study. Related to STAR Methods and Figures 1-5.

Table S2. List of all deep-sequencing libraries created in this study. Related to STAR Methods and Figures 3-5.

Data is available from GEO under accession no. GSE116329.
